# Neutrophils promote endothelial cell activation in pediatric *Mycoplasma pneumoniae* pneumonia

**DOI:** 10.1172/jci.insight.203856

**Published:** 2026-09-22

**Authors:** Xia Huang, Yifan Zhu, Yun Guo, Tian Lv, Haiyan Gu, Yingying Luo, Dan Li, Hang Sun, Deyu Zhao, Feng Liu

**Affiliations:** 1 Department of Respiratory Medicine, Children’s Hospital of Nanjing Medical University, Nanjing, China.; 2 Department of Respiratory Medicine & Clinical Allergy Center, Affiliated Children’s Hospital of Jiangnan University (Wuxi Children’s Hospital), Wuxi, China.

**Keywords:** Immunology, Infectious disease, Endothelial cells, Neutrophils, Thrombosis

## Abstract

*Mycoplasma pneumoniae* pneumonia (MPP) can cause serious extrapulmonary complications, including life-threatening thrombosis. This study aimed to elucidate the roles of neutrophils and neutrophil extracellular traps (NETs) in vascular endothelial cell (EC) activation in pediatric MPP-associated thrombosis. We analyzed the relationship between neutrophils and thrombosis in children with MPP and used mouse models of neutrophilia (*Csf3* plasmid injection), neutropenia (*Csf3* deficient*, Csf3^–/–^*), and defective NETs formation (*Pad4* deficient, *Pad4^–/–^*). The effects of neutrophils and NETs on EC activation were further examined in vivo, in vitro, and in human samples. Elevated neutrophil count was observed in patients with thrombosis and functioned as a potential diagnostic marker as well as a risk factor for MPP-associated thrombosis. EC activation was enhanced in MPP mice with neutrophilia but attenuated in neutropenic or *Pad4^–/–^* mice. NETs activated ECs through TLR2 and JAK/STAT3 signaling, and inhibition of NETs formation (Cl-amidine), TLR2 (C29), and JAK (upadacitinib) each attenuated this response. Strong correlations among neutrophils, NETs, EC activation, and thrombosis were observed in pediatric patients. These findings suggest that neutrophils promoted thrombosis in MPP via NETs-mediated EC activation involving TLR2 and JAK/STAT3 signaling. This study provides mechanistic insights into the inflammatory-thrombotic processes in MPP-associated thrombosis and offers a rationale for further investigation of neutrophils, NETs, TLR2, and JAK/STAT3 signaling in this context.

## Abstract 2

Neutrophils promoted thrombosis in *Mycoplasma pneumoniae* pneumonia via neutrophil extracellular traps-mediated endothelial activation involving TLR2 and JAK–STAT3 signaling

## Graphical abstract

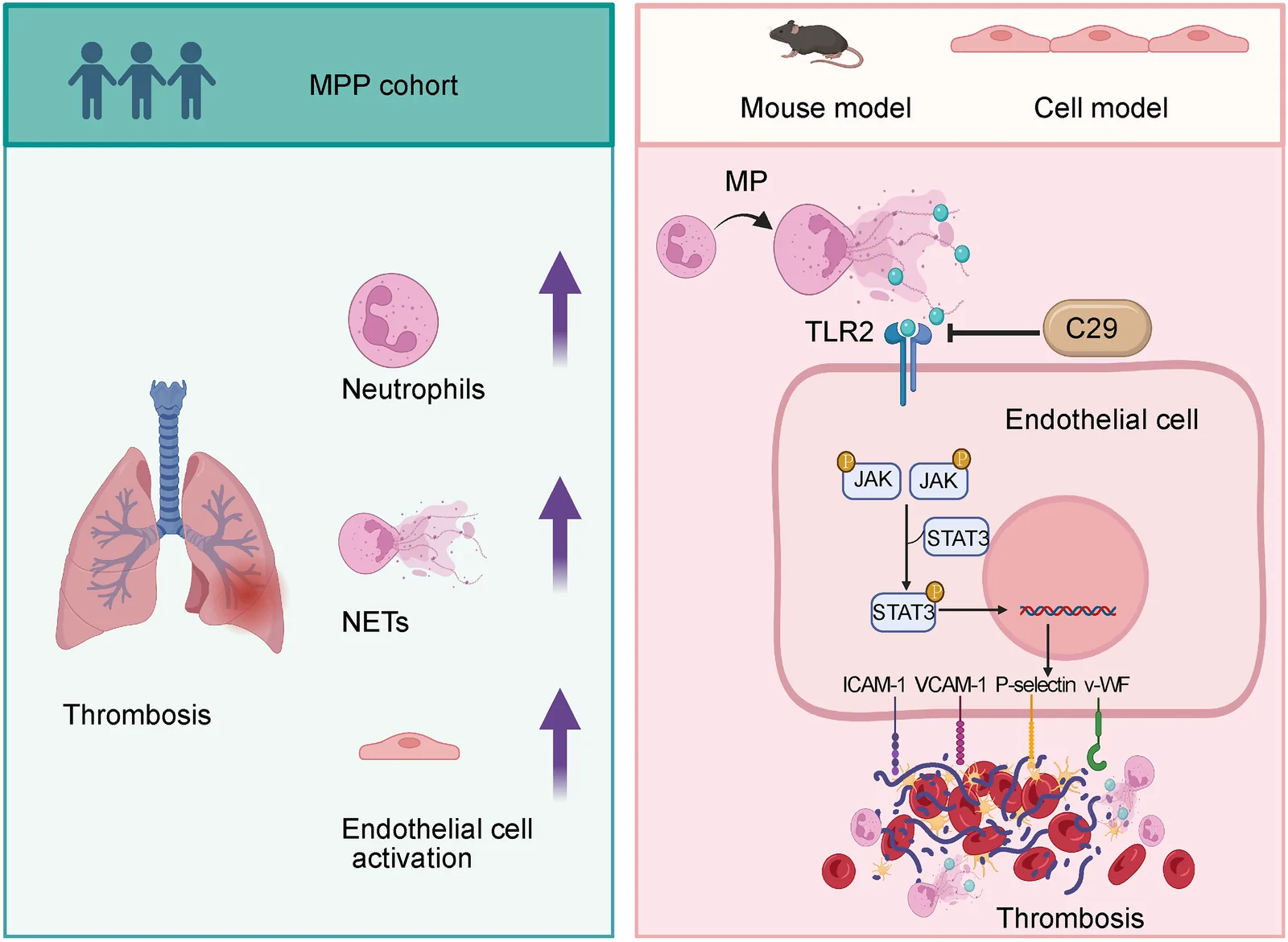

## Introduction

*Mycoplasma pneumoniae* (MP) is one of the most significant pathogens causing community-acquired pneumonia in children. Globally, MP infections occur in regional outbreaks every 3–7 years. Following the easing of COVID-19 pandemic restrictions, there has been a global increase in respiratory infections in children attributable to MP (1–3). Traditionally, MP infections were thought to be self-limiting. However, in recent years, many reports have described cases of refractory MP pneumonia (MPP), which may be accompanied by potentially life-threatening extrapulmonary complications, such as necrotizing pneumonia, bronchiolitis obliterans (BO), and thrombosis (4). The presence of thrombosis often indicates a more severe condition, including pulmonary, cardiac, deep vein, and cerebral vascular thrombosis (5–7), posing a serious threat to the life and health of children. However, the mechanisms underlying thrombosis formation in MPP are still not well understood.

Previous studies have consistently demonstrated that MP infection leads to a substantial increase in neutrophil counts in peripheral blood (8), as well as accumulation in bronchoalveolar lavage fluid (BALF) (9), and lung tissue (10). Neutrophil counts have been associated with greater severity of MPP and adverse outcomes, such as necrotizing pneumonia (11) and respiratory failure (9). An analysis of 43 cases of MPP with thrombosis at Beijing Children’s Hospital (Beijing, China) revealed a significant increase in the proportion of peripheral blood neutrophils (4). Neutrophils have been implicated in thrombosis across a spectrum of infectious and noninfectious conditions, including COVID-19 (12), sepsis (13), inflammatory bowel disease (14), antiphospholipid syndrome (15), and atherosclerosis (16). Endothelial cell (EC) activation plays a pivotal role in thrombus formation (13, 17, 18) and is characterized by upregulation of intercellular adhesion molecule-1 (ICAM-1), vascular cell adhesion molecule-1 (VCAM-1), P-selectin, and von Willebrand factor (vWF) (19, 20). A recent study suggests that MPP-associated thrombosis may frequently arise through local immunothrombotic processes within the affected vasculature rather than through classical thromboembolism (21). These observations raise the possibility that neutrophil-mediated EC activation may represent an important mechanism linking inflammation to thrombosis in MPP.

However, how neutrophils and NETs contribute to EC activation and thrombosis during MP infection remains unclear. Therefore, we integrated clinical data, mouse models, and in vitro and ex vivo analyses to test the hypothesis that neutrophils drive MPP-associated thrombosis by promoting NETs-dependent EC activation, focusing on TLR2 and JAK/STAT3 signaling as potential mechanistic pathways.

## Results

### Neutrophil elevation as a risk factor for thrombosis in children with MPP.

A total of 9,661 patients with community-acquired pneumonia were initially identified. After applying the inclusion criteria, 1,508 patients diagnosed with MPP were enrolled in the study, including 61 with thrombosis and 1,447 without thrombosis (Supplemental Figure 1; supplemental material available online with this article; https://doi.org/10.1172/jci.insight.203856DS1). The demographic and baseline clinical characteristics of the 1,508 children with MPP are presented in Table 1. The mean age of patients with MPP was 5.0 years. Among them, 61 children (4.1%) developed thrombotic complications, including 3 cases of cerebral embolism, 56 of pulmonary embolism, and 2 of deep vein thrombosis. Thrombosis occurred between 5 and 40 days after fever onset, with a median duration of 17 days.

**Figure 1:**
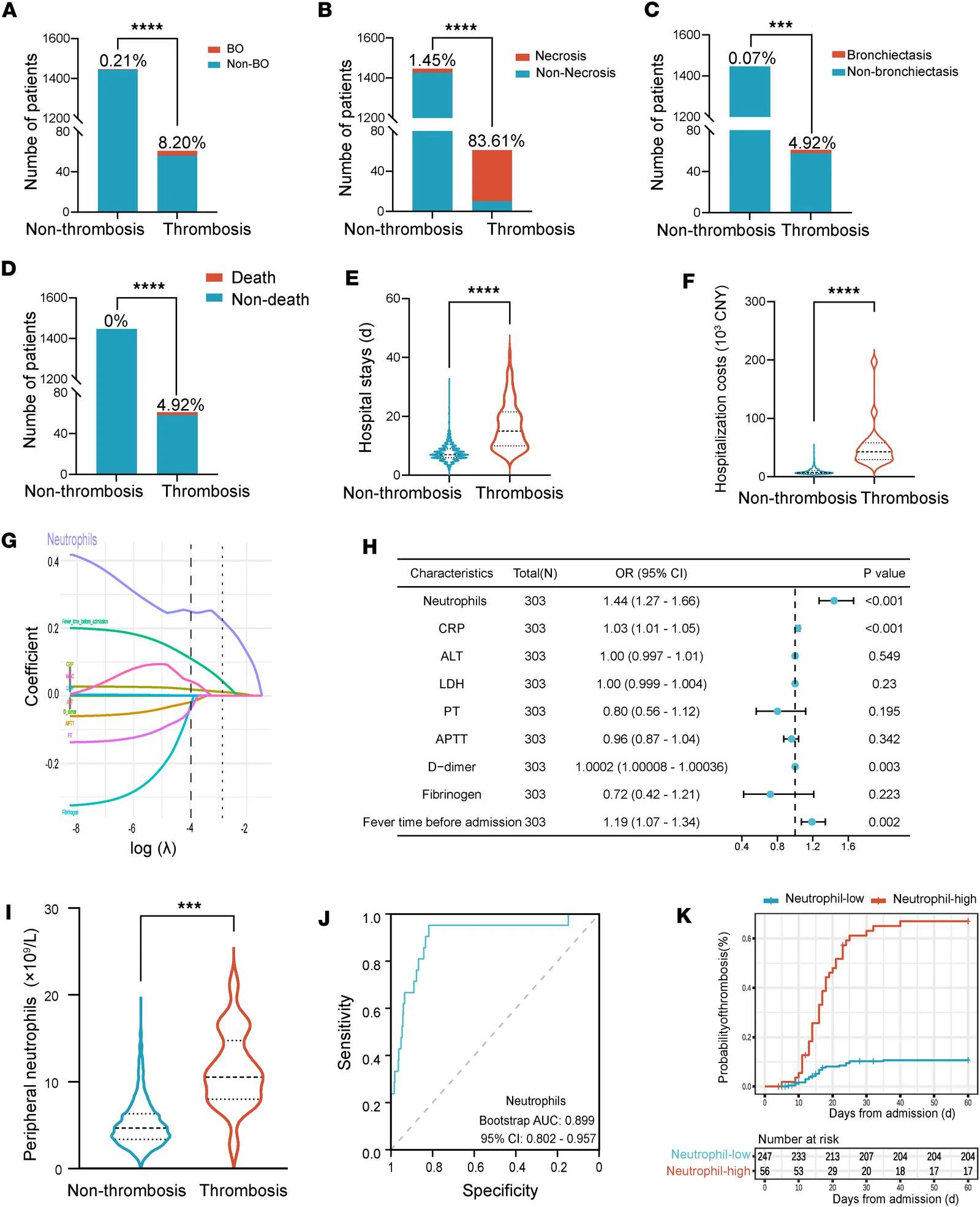
Neutrophil count as a key indicator associated with thrombosis the *Mycoplasma pneumoniae* pneumonia (MPP) cohort. (**A**–**D**) Proportions of patients with bronchial obstruction (BO), necrosis, bronchiectasis, and mortality in the thrombosis versus nonthrombosis groups (*n* = 1,508). Categorical variables were compared using χ² tests and are presented as percentages. (**E** and **F**) Comparison of hospitalization duration (**E**) and costs (**F**) between thrombosis and nonthrombosis groups (*n* = 1,508). Data are presented as median (IQR) and compared using the Mann-Whitney *U* test. (**G**) LASSO coefficient profiles of candidate laboratory indicators. Each curve represents the trajectory of a variable’s regression coefficient as a function of log (λ) (*n* = 1508). The vertical dashed line indicates the optimal penalty parameter (λ) selected by cross-validation. (**H**) Forest plot of multivariable Firth’s penalized logistic regression analysis in the propensity score–matched (PSM) cohort (*n* = 303). Neutrophil count, C-reactive protein (CRP), D-dimer, and preadmission fever duration were independently associated with thrombosis. Odds ratios (ORs) with 95% CIs and corresponding *P* values are shown. (**I**) Comparison of peripheral neutrophil counts between patients with and without thrombosis. Violin plots illustrate the distribution of neutrophil levels in each group (*n* = 303). Statistical significance was assessed using the Mann-Whitney *U* test. (**J**) Receiver operating characteristic (ROC) curve evaluating the discriminative performance of neutrophil count for thrombosis (*n* = 303). Internal validation was performed using bootstrap resampling (1,000 iterations). The bootstrap-corrected area under the curve (AUC) was 0.899, with a 95% CI of 0.802–0.957. (**K**) Kaplan-Meier curves showing the cumulative probability of thrombosis stratified by neutrophil count (high versus low) in the PSM cohort (*n* = 303). Differences between groups were assessed using the log-rank test. The number of patients at risk at each time point is shown below the plot. ***P < 0.001, ****P < 0.0001.

Patients with thrombosis had significantly higher rates of BO (8.20% versus 0.21%, *P* < 0.001), necrosis (83.61% versus 1.45%, *P* < 0.001), bronchiectasis (4.92% versus 0.07%, *P* < 0.001), and mortality (4.92% versus 0%, *P* < 0.001) than those without thrombosis. Additionally, patients in the thrombosis group experienced longer hospital stays (15 days versus 7 days, *P* < 0.001) and higher hospitalization costs (42.71 × 10^3^ versus 7.34 × 10^3^ Chinese yuan [CNY], *P* < 0.001) than patients without thrombosis (Figure 1, A–F).

Patients with thrombosis exhibited higher white blood cell (WBC) and neutrophil counts, C-reactive protein (CRP), alanine transaminase (ALT), aspartate transaminase (AST), lactate dehydrogenase (LDH), D-dimer levels, and preadmission fever duration, whereas lymphocyte counts, hemoglobin, and activated partial thromboplastin time (APTT) were lower than in nonthrombosis patients (Table 1). To minimize baseline bias, a 1:4 propensity score-matched (PSM) cohort was established, comprising 61 thrombosis and 242 nonthrombosis patients, with laboratory differences remaining consistent (Supplemental Figure 1 and Table 1). Absolute standardized mean differences before and after matching are presented in Supplemental Figure 2.

In the PSM cohort (*n* = 303), least absolute shrinkage and selection operator (LASSO) regression was used to identify key laboratory predictors of thrombosis (Figure 1G). The variables retained by LASSO, together with their penalized coefficients, are summarized in Supplemental Table 1. Neutrophil count emerged as a principal variable and was subsequently included in a Firth’s penalized logistic regression model with the selected covariates. To minimize structural collinearity, WBC count was excluded because neutrophils constitute a major proportion of circulating leukocytes. Multivariable analysis identified neutrophil count, CRP, D-dimer, and preadmission fever duration as independent predictors of thrombosis, with neutrophil count showing the strongest association (OR = 1.44; 95% CI, 1.27–1.66; *P* < 0.001; Figure 1H).

Neutrophil counts were significantly higher in patients with thrombosis (Table 1 and Figure 1I) and showed a positive correlation with D-dimer levels (r = 0.439, *P* < 0.001; Supplemental Figure 3A). Discriminative performance was evaluated using a bootstrap-corrected receiver operating characteristic (ROC) curve, with an AUC of 0.899 (95% CI, 0.802–0.957; Figure 1J). Patients were stratified into high- and low-neutrophil groups based on the optimal cutoff (8.82 × 10^9^/L) determined by X-tile software and were followed for 60 days. During follow-up, 21 patients (6.9%) were lost to follow-up, and no deaths occurred before the onset of thrombosis. The cumulative incidence of thrombosis was significantly higher in the high-neutrophil group than in the low-neutrophil group (log-rank *P* < 0.001; Figure 1K). Internal sensitivity analyses confirmed the robustness of the association between neutrophil count and thrombosis risk, both as a continuous variable and across neutrophil quartiles (Supplemental Table 2 and Supplemental Figure 3B).

### Neutrophils promote EC activation in MPP mice.

**Figure 2:**
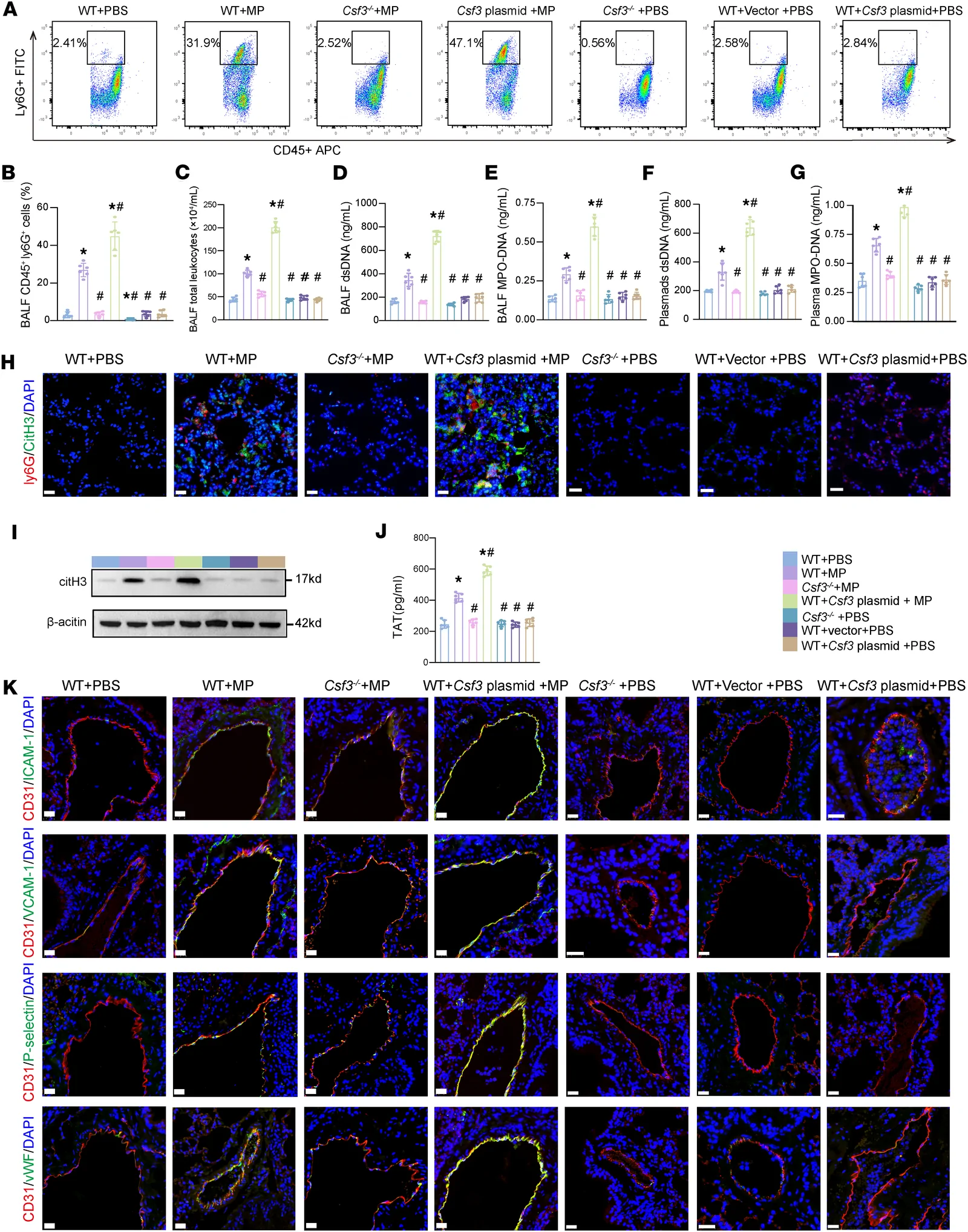
Neutrophils induce endothelial activation in MPP mice (*n* = **6 mice per group).** (**A** and **B**) Flow cytometric analysis and quantification of CD45 ^+^ Ly6G ^+^ cells in BALF from MPP mice with varying neutrophil levels. CD45 ^+^ Ly6G ^+^ cells represent mouse neutrophils. Representative flow cytometry plots are shown, and quantitative analysis was performed. (**C**) Total cell counts in BALF from MPP mice with varying neutrophil levels. (**D**–**G**) Levels of double-stranded DNA (dsDNA) and MPO-DNA in plasma and BALF. (**H**) Representative immunofluorescence images of NETs in lung sections from MPP mice, showing colocalization of Ly6G (red), CitH3 (green), and DAPI (blue). Scale bar: 40 μm. (**I**) Representative Western blot analysis of CitH3, a marker of NETs formation, in lung tissue from MPP mice. (**J**) Plasma levels of thrombin-antithrombin (TAT) complexes measured by enzyme-linked immunosorbent assay. (**K**) Representative immunofluorescence staining of pulmonary ECs for activation markers: ICAM-1, VCAM-1, P-selectin, and von Willebrand factor (vWF). Endothelial cells (ECs) were labeled with CD31 (red), activation markers (green), and DAPI (blue). Scale bars: 20 μm. Data are presented as mean ± SD and were analyzed by 1-way ANOVA followed by the Games-Howell post hoc test (**B**–**G** and **J**). *P < 0.05 versus WT + PBS group; ^#^*P* < 0.05 versus WT + MP group.

Given the pivotal role of EC activation in thrombosis, we investigated the contribution of neutrophils to EC activation in MPP mice. We developed MPP mouse models with varied neutrophils levels by intratracheally administering MP into WT, neutropenia (*Csf3* deficient*, Csf3^–/–^*), and neutrophilia mice (induced by *Csf3* plasmid injection) (Supplemental Figure 4). Gene expression profiles in *Csf3* ^–/–^ mice are presented in Supplemental Figure 4A. Peripheral blood neutrophil counts were significantly higher in WT mice treated with the *Csf3* plasmid (38.97 × 10^9^/L versus 0.6 × 10^9^/L, *P* < 0.001), representing neutrophilia. In contrast, *Csf3*^–/–^ mice exhibited a greater reduction in neutrophil counts (0.2 × 10^9^/L versus 0.6 × 10^9^/L, *P* < 0.001) than those in untreated WT mice, reflecting neutropenia (Supplemental Figure 4B). After MP challenge, neutrophilic mice showed intensified inflammatory cell recruitment in BALF and peripheral blood, while neutropenic mice showed reduced inflammation (Figure 2, A–C, and Supplemental Figure 5A). NETs release is a critical function of neutrophils and has been implicated in the pathogenesis of thrombosis. To evaluate NETs formation in MPP mice and its association with thrombosis, we measured levels of double-stranded DNA (dsDNA) and Myeloperoxidase-DNA (MPO-DNA) complex — 2 well-established markers of NETs — in peripheral blood and BALF (22), and assessed NETs in lung tissue using immunofluorescence staining and Western blot analysis. NETs levels were significantly increased in the blood, BALF, and lung tissue of MPP mice, with the highest levels observed in neutrophilic MPP mice. In contrast, NETs formation was markedly attenuated in neutropenic MPP mice (Figure 2, D–I). Consistent with these findings, plasma thrombin-antithrombin (TAT) levels, a marker of coagulation activation (23), increased in neutrophilic mice and decreased in neutropenic mice, indicating that neutrophil abundance influences thrombotic signaling during MP infection (Figure 2J).

We further assessed pulmonary EC activation by immunofluorescence staining. The expression of EC activation markers, including ICAM-1, VCAM-1, P-selectin, and vWF, was significantly increased in MPP mice treated with the *Csf3* plasmid and significantly reduced in *Csf3* ^–/–^ MPP mice compared with WT MPP mice (Figure 2K). These findings indicate that increased neutrophil accumulation promotes NETs formation and EC activation, whereas neutrophil depletion attenuates these responses, supporting a role for neutrophils in linking inflammation to EC activation and thrombosis in MPP.

### MP promotes the release of NETs from neutrophils.

**Figure 3:**
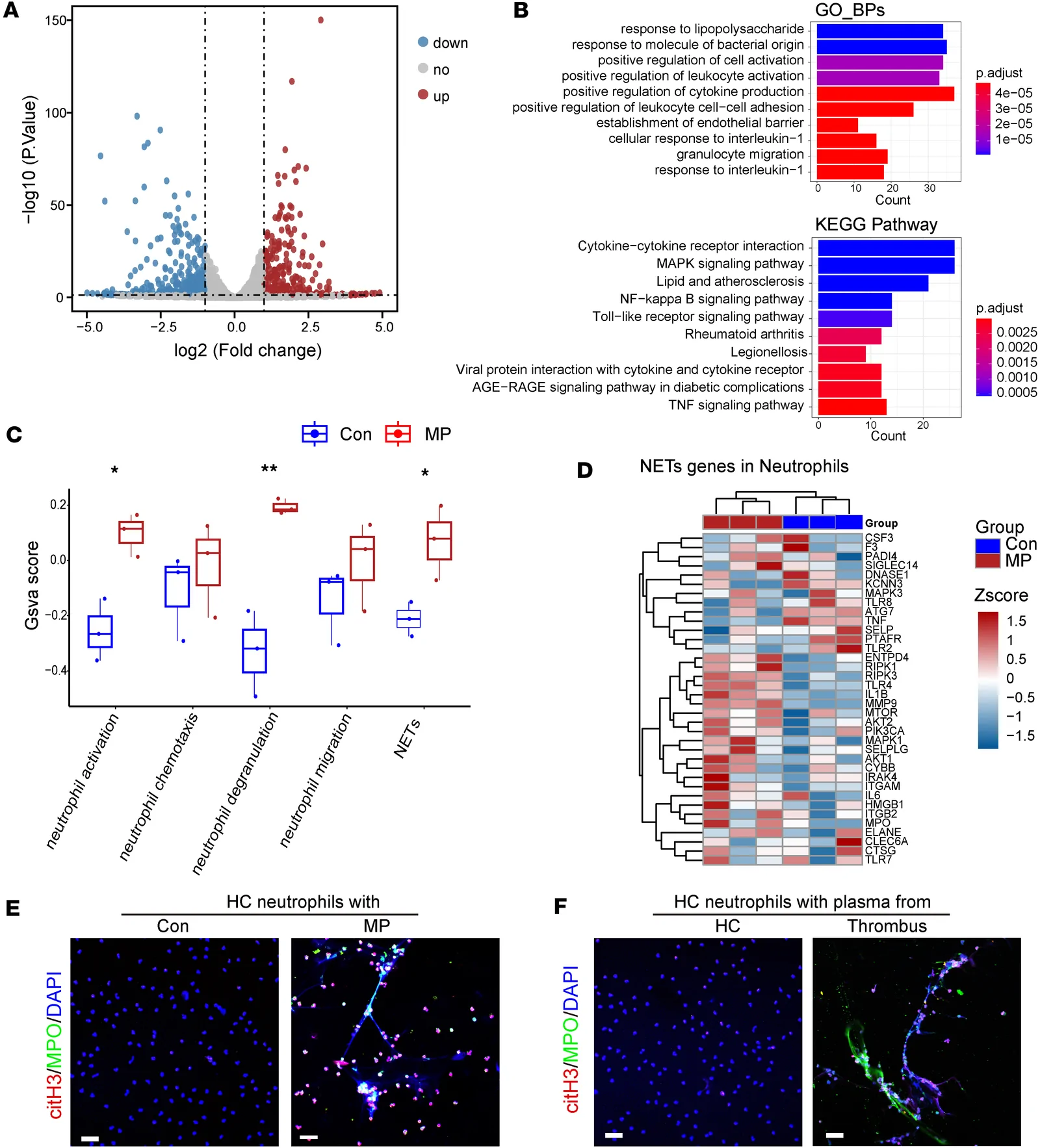
Mycoplasma pneumoniae (MP) promotes neutrophil NETs formation. (**A**) RNA-seq was performed on neutrophils isolated from healthy controls (HCs) and stimulated with MP or PBS (control), *n* = 3 donors per group. The volcano plot displays differentially expressed genes (DEGs) between the MP and control groups; red and blue dots indicate upregulated and downregulated genes, respectively. DEGs were identified using DESeq2 analysis. (**B**) Gene Ontology (GO) and Kyoto Encyclopedia of Genes and Genomes (KEGG) pathway enrichment analysis of DEGs. Biological processes (BPs), a major component of GO, are shown. (**C**) Gene set variation analysis (GSVA) comparing neutrophil functions between the MP and control groups. Differences in GSVA scores between groups were assessed using the limma package. Data are presented as median (IQR); *n* = 3 donors per group; *P < 0.05, **P < 0.01, ***P < 0.001, ****P < 0.0001. (**D**) Expression profiles of NETs-related genes in neutrophils. (**E** and **F**) Representative immunofluorescence images of NETs formation in HC neutrophils cocultured with PBS or MP (**E**) or plasma from HCs and patients with thrombosis (**F**). Neutrophils were stained for myeloperoxidase (MPO, green), citrullinated histone H3 (CitH3, red), and DAPI (blue). Representative images from 3 donors per group are shown. Scale bar: 25 μm.

RNA-seq of human neutrophils stimulated with MP revealed broad transcriptional reprogramming (464 upregulated and 361 downregulated genes; Figure 3A and Supplemental Figure 5B). Enriched functions included neutrophil migration, chemotaxis, adhesion, cytokine receptor pathways, MAPK, TLR, and NF-κB signaling (Figure 3B). Gene set variation analysis (GSVA) showed strong enrichment of neutrophil activation, degranulation, and especially NETs-formation pathways, with most NETs-related genes upregulated (Figure 3, C and D). MP strains and plasma from thrombotic patients triggered robust NETs release in vitro, indicating that MP and thrombotic host factors are sufficient to drive NETosis (Figure 3, E and F).

### Neutrophils promote EC activation through NETs in MPP mice.

**Figure 4:**
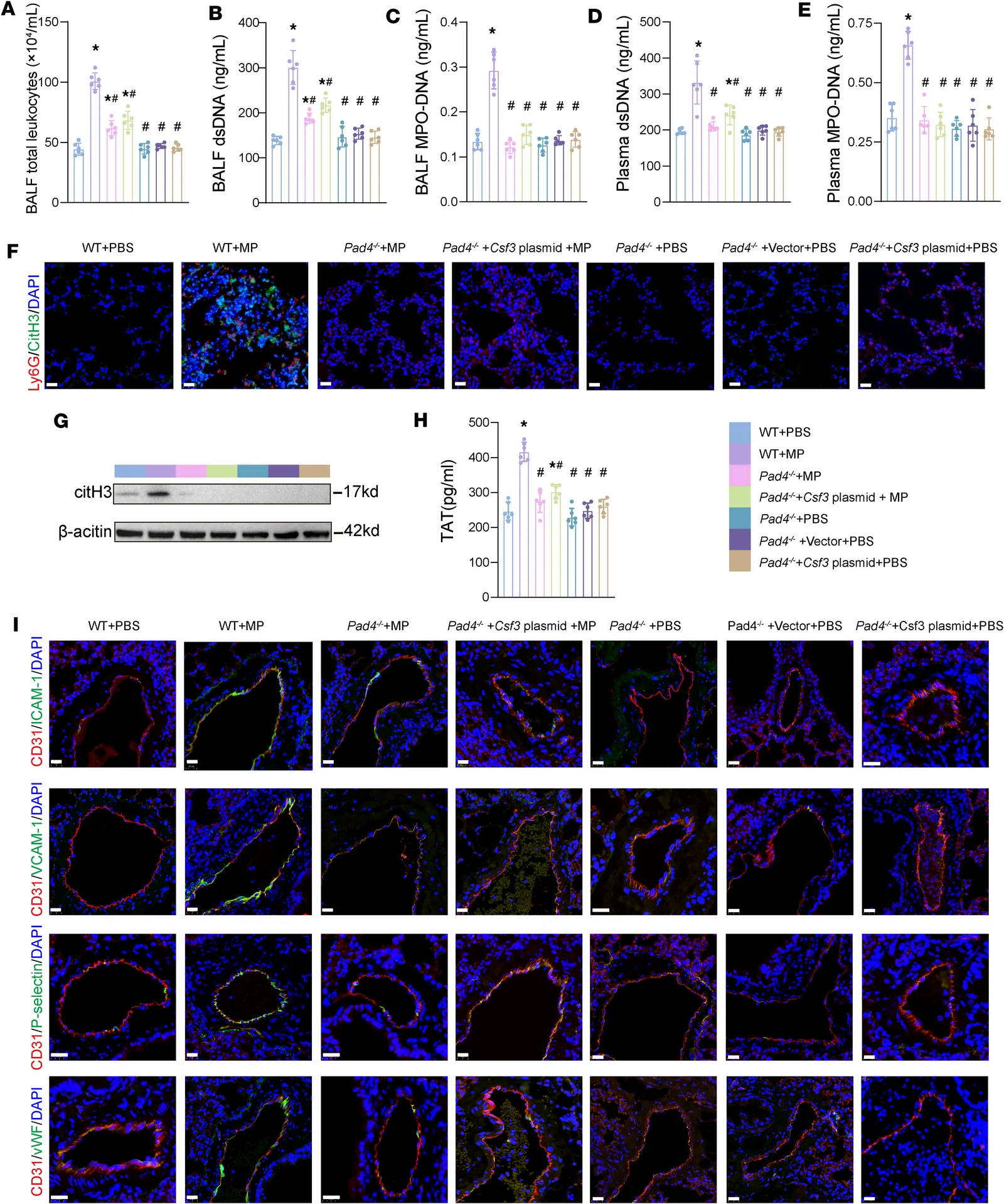
Neutrophils promote endothelial activation through NETs in MPP mice (*n* = 6 per group). (**A**) Total cell counts in the bronchoalveolar lavage fluid (BALF) of MPP mice with varying NETs levels. (**B**–**E**) Levels of double-stranded DNA (dsDNA) and MPO-DNA in plasma and BALF. (**F**) Representative immunofluorescence images of NETs in lung sections. NETs are visualized by colocalization of Ly6G (red), CitH3 (green), and DAPI (blue). Scale bar: 20 μm. (**G**) Western blot analysis of NETs formation in lung tissue. (**H**) Plasma levels of thrombin-antithrombin (TAT) complexes. (**I**) Representative immunofluorescence staining of pulmonary endothelial cells (ECs) for markers of endothelial activation: ICAM-1, VCAM-1, P-selectin, and von Willebrand factor (vWF). ECs were labeled with CD31 (red), activation markers (green), and DAPI (blue). Scale bar: 20 μm. Quantitative data are presented as mean ± SD and were analyzed by 1-way ANOVA followed by the Games-Howell post hoc test (**A**–**E** and **H**). *P < 0.05 versus WT + PBS group; ^#^*P* < 0.05 versus WT + MP group.

To isolate the role of NETs, we used *Pad4^–/–^* mice (defective NETs formation) with or without *Csf3*-induced neutrophilia. Gene identification results for the *Pad4^–/–^* group are shown in Supplemental Figure 4C. MP-infected *Pad4^–/–^* mice exhibited reduced inflammatory cell infiltration (Figure 4A); markedly lower NETs levels across blood, BALF, and lung (Figure 4, B–G); and reduced plasma TAT complexes (Figure 4H). Importantly, EC activation markers were significantly lower in *Pad4^–/–^* MPP mice than in WT MPP mice, and neutrophilia could not restore EC activation in the absence of NETs (Figure 4I). Thus, neutrophils promote EC activation during MPP primarily through NETs formation.

### NETs contribute to EC activation via TLR2 and JAK/STAT3.

We further explored the role and underlying mechanisms by which MP-induced NETs — formed upon stimulation of neutrophils by MP — activate ECs. Human umbilical vein endothelial cells (HUVECs) treated with MP-induced NETs showed strong upregulation of ICAM-1, VCAM-1, P-selectin, and vWF (Supplemental Figure 6A).

**Figure 5:**
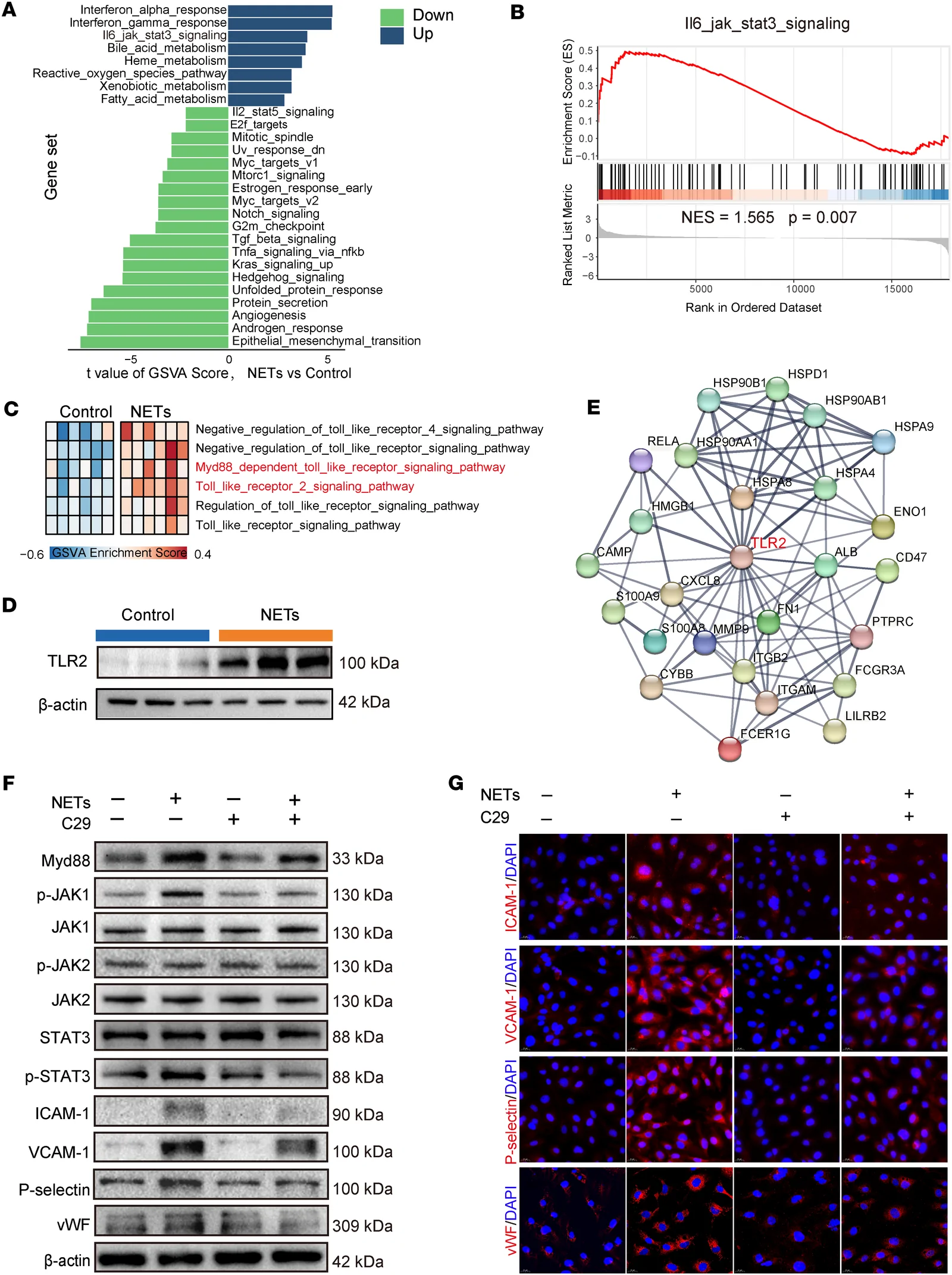
NETs promote endothelial activation through TLR2 and JAK/STAT3 signaling. (**A** and **B**) RNA-seq analysis of HUVECs cultured with or without MP-induced NETs. Gene set variation analysis (GSVA) and gene set enrichment analysis (GSEA) were performed to compare pathway activity between NETs-treated and control groups using the limma package (*n* = 6 biological replicates). (**C**) GSVA analysis showing activation of TLR-related signaling pathways. Differences in GSVA scores were assessed using the limma package. (**D**) Representative Western blot analysis of TLR2 expression in HUVECs cultured with or without MP-induced NETs. Data were obtained from 3 independent experiments. (**E**) Protein-protein interaction (PPI) network showing associations between TLR2 and MP-NETs–related proteins. (**F** and **G**) HUVECs were pretreated with or without the TLR2 inhibitor C29 before stimulation with MP-induced NETs. Western blotting and immunofluorescence staining were performed to evaluate JAK/STAT3 signaling proteins and endothelial activation markers. Representative images and blots from 3 independent experiments are shown.

RNA-seq of NETs-stimulated HUVECs identified 361 differentially expressed genes (DEGs), including 195 upregulated and 166 downregulated genes, as illustrated in the volcano plot and heatmap (Supplemental Figure 7, A and B). Enriched pathways included cytokine signaling, calcium signaling, and JAK/STAT3 signaling (Supplemental Figure 7, C–F). Among these pathways, JAK/STAT3 signaling was the most significantly enriched pathway and was therefore prioritized for further validation. GSVA showed pronounced activation of JAK/STAT3 signaling pathway (Figure 5, A and B), which includes key components, such as Jak1, Jak2, and Stat3. Notably, the expression levels of Jak1 and Stat3 were significantly higher in the NETs-stimulated group than in the control group (*P* < 0.001; Supplemental Figure 8).

Given the well-established role of TLRs in EC activation (24–26), we further assessed the activation status of TLR-related pathways using GSVA based on the GO database. This analysis revealed significant activation of the TLR2 signaling pathway and the MyD88-dependent TLR pathway in NETs-stimulated HUVECs (Figure 5C). Western blot analysis further confirmed increased expression of TLR2 following NETs stimulation (Figure 5D). MP-related NETs proteins were showed in Supplemental Table 3. To identify the specific MP-NETs proteins responsible for activating TLR2, we used the STRING database to construct a protein-protein interaction (PPI) network. Through this analysis, we identified 25 MP-NETs–related proteins closely associated with TLR2 and potentially involved in its activation (Figure 5E).

Functionally, MP stimulation significantly increased extracellular dsDNA release, reflecting NETs formation, which was reduced by Cl-amidine (Cl) (Supplemental Figure 6B). MP-induced NETs-enriched conditioned medium activated TLR2/MyD88 signaling and selectively induced phosphorylation of JAK1 and STAT3, concomitant with elevated ICAM-1, VCAM-1, P-selectin, and vWF, whereas Cl inhibition largely abolished these effects (Supplemental Figure 6B). Pharmacological blockade of TLR2 with C29 attenuated MyD88 and JAK1/STAT3 activation and reduced endothelial activation marker expression

(Figure 5, F and G). Consistent reductions in membrane-associated ICAM-1, VCAM-1, and P-selectin were observed following TLR2 inhibition (Supplemental Figure 6C). Similarly, MyD88 inhibition with TJ-M2010-5 (TJ) suppressed JAK1 and STAT3 phosphorylation (Supplemental Figure 6D), and JAK1 inhibition with upadacitinib (UPA) decreased STAT3 activation and EC activation marker expression (Supplemental Figure 6E). Finally, similar EC activation was observed in human pulmonary microvascular endothelial cells (HPMECs) following MP-NETs treatment, indicating that the effects of MP-NETs extend across distinct EC types (Supplemental Figure 6F).

**Figure 6:**
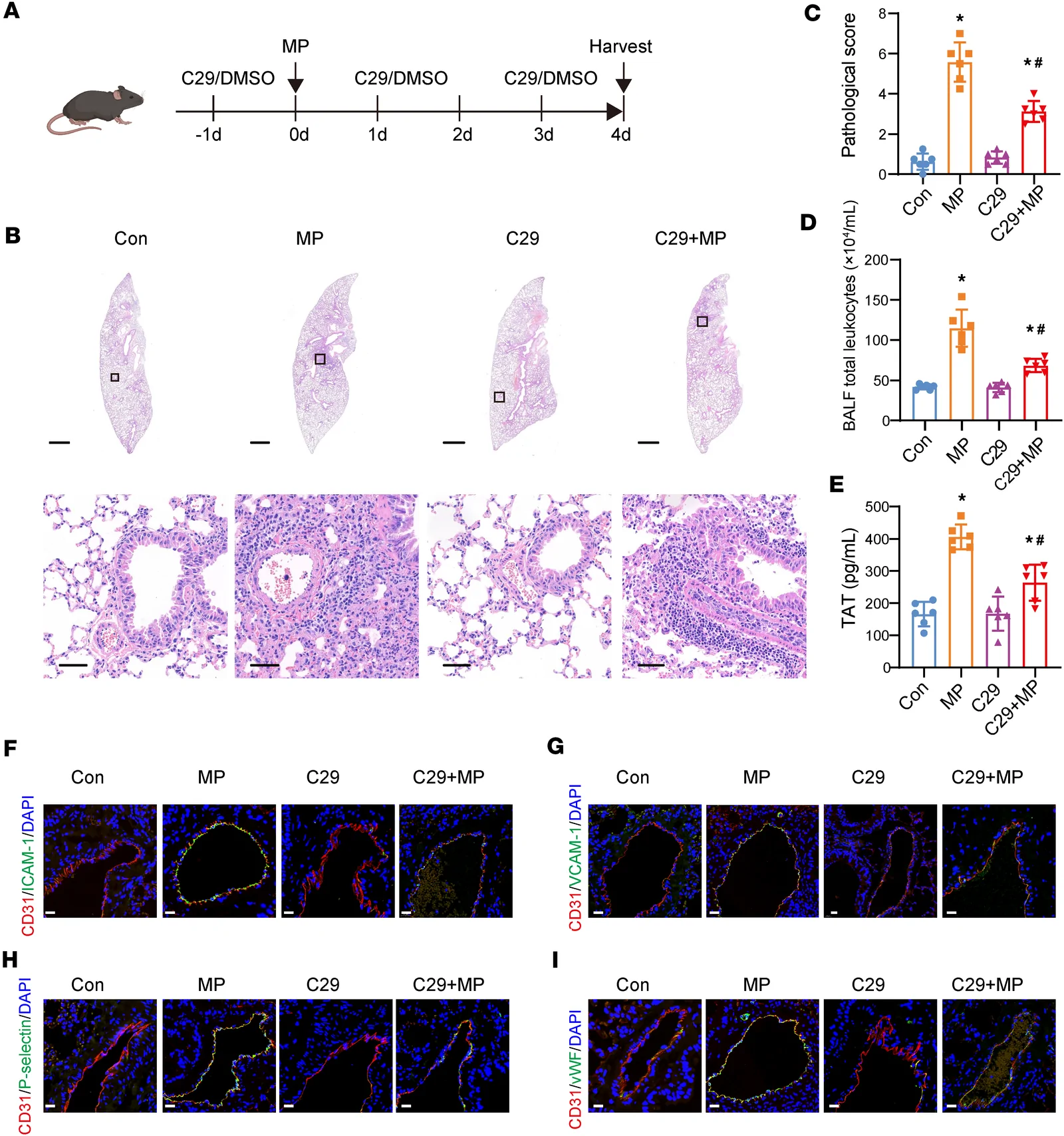
TLR2 inhibition ameliorates endothelial activation in MPP mice (*n* = 6 mice per group). (**A**) Mice received i.p. injections of the TLR2 antagonist C29 (30 mg/kg) every other day for 3 doses, beginning 24 hours before intratracheal administration of MP. (**B**) Representative H&E-stained lung sections from each group are shown. Top panel, scale bars: 1,000 μm; bottom panel, scale bars: 50 μm. (**C**) Semiquantitative assessment of lung pathological changes based on H&E staining, with recorded pathological scores. (**D**) Total cell counts in bronchoalveolar lavage fluid (BALF). (**E**) Plasma levels of thrombin-antithrombin (TAT) complexes. (**F**–**I**) Representative immunofluorescence images showing expression of endothelial activation markers: ICAM-1 (**F**), VCAM-1 (**G**), P-selectin (**H**), and von Willebrand factor (vWF) (**I**) on pulmonary endothelial cells (ECs). ECs were stained with CD31 (red), activation markers (green), and DAPI (blue). Scale bars: 20 μm. Quantitative data are presented as mean ± SD and were analyzed by 1-way ANOVA followed by the Games-Howell post hoc test (**C**–**E**). *P < 0.05 versus control (Con) group; ^#^*P* < 0.05 versus MP group.

In MPP mice, C29 treatment lowered inflammation (Figure 6, A–D), improved coagulation indices (Figure 6E), reduced pathological injury, and suppressed EC activation (Figure 6, F–I).

### Neutrophils and NETs formation is associated with EC activation in patients with MPP.

We conducted a comprehensive analysis of blood, embolized lung, and thrombus tissues in the propensity score–matched MPP cohort. We conducted a comprehensive analysis of clinically discarded blood samples, embolized lung tissues, and thrombus tissues from patients corresponding to the MPP cases. In total, 303 blood samples, 2 embolized samples, and 1 lung tissue sample were collected. Because lung tissues could not be obtained from nonthrombus patients, lung lobes resected from 2 children who underwent elective surgery for congenital lung cysts were used as control samples (Supplemental Table 4).

**Figure 7:**
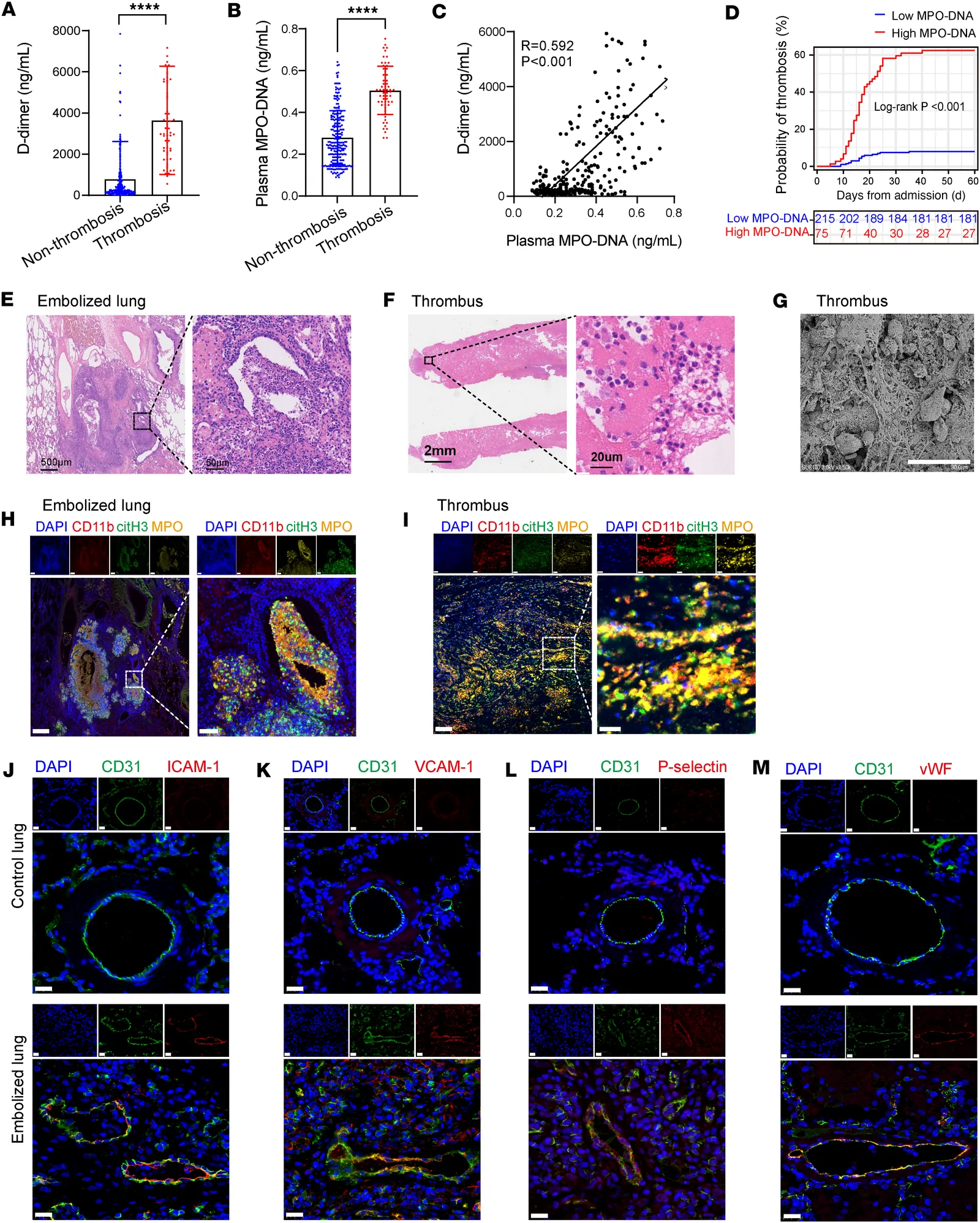
NETs and endothelial activation in blood samples and lung tissues from patients with thrombosis in the PSM MPP cohort. (**A**) Plasma D-dimer levels in the PSM MPP cohort (*n* = 303). Data are presented as median (IQR) and were analyzed using the Mann-Whitney *U* test. (**B**) Plasma levels of MPO-DNA in the PSM MPP cohort (*n* = 303). Data are presented as median (IQR) and were analyzed using the Mann-Whitney *U* test. *P < 0.05 versus Con group; ^#^*P* < 0.05 versus MP group. (**C**) Correlation between plasma MPO-DNA levels and D-dimer levels (*n* = 303). Spearman’s rank correlation analysis was performed. (**D**) Kaplan-Meier curves showing the cumulative probability of thrombosis stratified by plasma MPO-DNA count (high versus low) (*n* = 303). Differences between groups were assessed using the log-rank test. (**E**) Representative H&E staining of embolized lung tissue from a patient with MPP-associated thrombosis. Left panel, scale bar: 500 μm; right panel, scale bar: 50 μm. (**F**) Representative H&E staining of thrombus tissue from 2 patients with MPP-associated thrombosis. Left panel, scale bar: 2 mm; right panel, scale bar: 20 μm. (**G**) Representative scanning electron microscopy images of thrombus tissues from 2 patients with MPP-associated thrombosis showing extracellular web-like structures in close proximity to neutrophils. Scale bar: 20 μm. (**H**) Representative immunofluorescence images showing NETs formation in embolized lung tissue from 1 patient with MPP-associated thrombosis. Colocalization of CD11b (red), CitH3 (green), MPO (yellow), and DAPI (blue) indicates NETs formation. Left panel, scale bar: 500 μm; right panel, scale bar: 50 μm. (**I**) Representative immunofluorescence images of NETs in thrombus tissue from 2 patients with MPP-associated thrombosis. Left panel, scale bar: 100 μm; right panel, scale bar: 20 μm. (**J**–**M**) Representative immunofluorescence staining of pulmonary ECs for markers of endothelial activation: ICAM-1 (**J**), VCAM-1 (**K**), P-selectin (**L**), and vWF (**M**). ECs were labeled with CD31 (red), target activation markers (green), and DAPI (blue). Representative images from one patient with MPP-associated thrombosis are shown. Scale bar: 20 μm. ****P < 0.0001.

Patients with thrombosis showed significantly higher D-dimer levels and increased plasma NETs concentrations (all *P* < 0.001; Figure 7, A and B, and Supplemental Figure 9A). Plasma NETs was positively correlated with D-dimer and neutrophil counts (Figure 7C and Supplemental Figure 9, B–D). Elevated plasma MPO-DNA levels were associated with a significantly increased risk of thrombosis in the PSM cohort (log-rank *P* < 0.001) (Figure 7D). Histological analysis of embolized lung and thrombus tissue demonstrated marked neutrophil infiltration (Figure 7, E and F). Scanning electron microscopy revealed extracellular web-like structures in close proximity to neutrophils, and NETs formation was confirmed by immunofluorescence staining (Figure 7, G–I). Pulmonary vascular ECs from thrombotic children displayed strong ICAM-1, VCAM-1, P-selectin, and vWF expression (Figure 7, J–M), confirming endothelial activation associated with NETs-rich inflammation.

## Discussion

This study establishes neutrophils and NETs as key drivers of thrombosis in pediatric MPP. Although thrombosis occurred in only 4.1% of instances, it was associated with severe pulmonary pathology, frequent chronic airway complications (BO, bronchiectasis), increased mortality, and high healthcare burden. Clinically, neutrophil count stood out as the strongest thrombosis predictor, outperforming multiple inflammatory and coagulation indices, and correlated with D-dimer, indicating a close relationship between neutrophilic inflammation and thrombotic activity.

Previous case-control studies with small sample sizes have suggested that patients with MP-associated thrombosis exhibit elevated neutrophil counts (27, 28). Building on these findings, we conducted a retrospective cohort study encompassing 1,468 children hospitalized for MPP over a 3-year period confirming a significant increase in neutrophil counts among patients with MP-associated thrombosis. Elevated neutrophil counts were further identified as a risk factor for thrombosis and demonstrated strong predictive value for its diagnosis. Neutrophil infiltration is widely recognized as a hallmark of MPP (8, 10, 29). However, increasing neutrophil counts does not appear to facilitate the clearance of MP. In contrast, neutrophil depletion or inhibition of their function can reduce pulmonary inflammation (10, 30) and mitigate the formation of in situ pulmonary thrombi (31). Emerging evidence supports that thrombosis in MPP is more likely to represent in situ immunothrombosis occurring within the pulmonary vasculature rather than embolic events (21). ECs, which line the interior surface of blood vessels and are in direct contact with circulating blood, can adopt a prothrombotic phenotype in response to various systemic or local injuries, thereby promoting thrombus initiation (32, 33). We therefore focused our analyses on EC activation as a key mechanistic link between inflammation and thrombosis.

Several studies have implicated NETs in thrombotic processes (12, 14, 16). NETs are web-like structures released by activated neutrophils, consisting of a DNA scaffold decorated with histones, neutrophil elastase, MPO, and antimicrobial peptides (34). The formation mechanisms and composition of NETs can vary depending on the stimulus (35). During disease progression, NETs act as a double-edged sword: while they can enhance host defense by promoting pathogen clearance and contributing to hemostasis, excessive or dysregulated NETs formation can amplify inflammation, cause tissue damage, and promote thrombosis (36, 37). Our previous transcriptome analyses of blood and BALF from patients with MPP suggested a potential role for NETs in the pathogenesis of the disease (9, 38). In this study, we demonstrated that MP stimulates neutrophils to release NETs both in vivo and in vitro. Furthermore, a mouse model with impaired NETs formation revealed that neutrophils promote EC activation through NETs. These findings provide mechanistic evidence that NETs formation contributes to MP-associated thrombosis.

Engagement of pathogen recognition receptors such as TLRs triggers ECs to adopt a proinflammatory and proangiogenic phenotype (25, 26). Both in vitro and in vivo studies have demonstrated that endothelial activation contributes to thrombotic risk by upregulating vWF and P-selectin in JAK-mutated myeloproliferative neoplasms (39, 40). Downstream of JAK mutations, activation of the STAT3 pathway has been shown to increase the expression of EC adhesion molecules (41). Overactivation of STAT3 is also considered a key driver of coagulopathy and thrombosis associated with COVID-19 (42). In this study, transcriptome sequencing of HUVECs treated with MP-induced NETs revealed activation of TLR2 and the JAK/STAT3 signaling pathway. Although additional EC-activating pathways may be involved, the present study focused on the TLR2 and JAK/STAT3 because of its strong association with EC activation and thrombosis. In vitro inhibition of TLR2 suppressed MyD88 expression, downregulated JAK/STAT3 signaling, and attenuated EC activation. Similarly, TLR2 inhibition in the MPP mouse model reduced MP-induced inflammation and EC activation. These findings suggest that neutrophils promote EC activation through the NETs/TLR2 signaling axis (Supplemental Figure 10). In addition, recent studies in patients with dengue have shown that IgA complexes can activate neutrophils and induce endothelial damage (43). Although the upstream mechanism differs from NETs-mediated activation observed in MPP, these findings highlight the broader role of neutrophil-driven endothelial injury in infection-associated vascular complications.

Furthermore, the clinical manifestations of infection-induced endothelial dysfunction vary significantly depending on the underlying mechanisms. While direct pathogen toxicity, such as that caused by the dengue flavivirus NS1 protein, characteristically induces barrier hyperpermeability and vascular leakage (44–48), MPP-associated endothelial injury manifests primarily as a prothrombotic phenotype. Our findings suggest that this activation is primarily mediated by an excessive host immune response rather than direct microbial toxicity, whereby NETs activate ECs through the TLR2 and JAK/STAT3 signaling pathways to promote thrombosis.

However, several limitations of our study should be acknowledged. First, thrombosis is a complex and multifactorial process, and our investigation was confined to EC activation. Second, given the complex composition of NETs, we could not identify the specific components responsible for dysregulating endothelial and coagulation systems. Instead, NETs were treated as a collective entity in stimulating ECs. Third, the mechanistic interplay between TLR2 and JAK/STAT3 signaling pathways warrants further investigation. Fourth, bulk RNA-seq cannot resolve neutrophil heterogeneity, and *Pad4*-independent NETs formation cannot be completely excluded.

In conclusion, elevated neutrophil count represents an independent risk factor for thrombosis in pediatric MPP. Our in vitro and in vivo findings support a role for NETs-mediated EC activation through TLR2 and JAK/STAT3 signaling in the pathogenesis of MPP-associated thrombosis. These results provide insight into the inflammatory-thrombotic mechanisms underlying MPP-associated thrombosis and highlight neutrophils, NETs, TLR2, and JAK/STAT3 signaling as key components of this pathway that warrant further investigation.

## Methods

### Sex as a biological variable.

Male mice were exclusively used in this study to minimize potential confounding effects of sex hormones on neutrophil activation, NETs formation, and coagulation responses, as previous studies have suggested that sex hormones may influence these processes (49, 50). Whether these findings are applicable to female mice requires further investigation.

### Human populations and design.

We conducted a matched case-control study nested within a prospective cohort of children diagnosed with MPP who were admitted to the Department of Respiratory Medicine at the Children’s Hospital of Nanjing Medical University between January 2019 and December 2021. The inclusion criteria were as follows: (a) age between 28 days and 15 years; (b) clinical presentation with respiratory symptoms; (c) laboratory confirmation of MP infection by both serologic (antibody) and nucleic acid detection; and (d) radiographic evidence of pneumonia.

Exclusion criteria included: (a) history of congenital heart disease, immunodeficiency, hereditary neurological disorders, or coagulation abnormalities; (b) coinfection with other pathogens; (c) recent trauma or surgical procedures within the past month; (d) use of medications affecting blood coagulation or hematopoiesis; and (e) presence of an indwelling arteriovenous catheter.

Data were collected prospectively, including age, sex, and duration of fever before hospitalization. Laboratory assessments included WBC counts, differential counts for neutrophils and lymphocytes, and levels of CRP, ALT, AST, and LDH as well as coagulation profiles. Additionally, biological samples included peripheral blood samples collected within 24 hours of hospital admission as well as embolized lung and thrombus tissues. Control lung tissues were obtained from children undergoing pulmonary resection for noninfectious conditions, such as congenital pulmonary cysts.

Cases were defined as patients presenting with unexplained respiratory distress, chest pain, hemoptysis, syncope, or shock, along with unilateral or bilateral asymmetric lower limb swelling and pain, with or without elevated D-dimer levels. A diagnosis of thrombosis was confirmed through imaging studies, including computed tomography angiography, cerebral magnetic resonance angiography and venography, vascular ultrasound, or echocardiography. Control patients were defined as individuals within the MPP cohort who did not experience a thrombotic event within 60 days of admission.

All children were followed for 1 year from the date of admission. The primary outcome was the occurrence of thrombosis within 60 days. Patients lost to follow-up were censored at the date of their last clinical visit. Secondary outcomes included total fever duration, length of hospital stay, medical costs, and the development of complications, such as necrosis, BO, bronchiectasis, or death.

### MP culture.

The standard strain of MP, M129 B7 (ATCC29342), was obtained from the American Type Culture Collection (ATCC, USA). MP was cultured using *Mycoplasma* broth and agar plates (Oxoid, United Kingdom), supplemented with *Mycoplasma* Supplement G. The frozen M129 strain was resuscitated in a 37°C water bath, followed by incubation in liquid medium under 5% CO_2_ at 37°C. Once the culture medium turned orange — indicating sufficient growth — MP stocks were harvested. CFU were quantified by plating the bacterial suspension onto *Mycoplasma* agar plates and counting colonies after a 4-week incubation period. MP stocks were subsequently concentrated to a final titer of 1 × 10^8^ CFU/mL in *Mycoplasma* broth for use in experiments.

### Animals.

C57BL/6JGpt mice (6–8 weeks old; GemPharmatech Co.), a C57BL/6J-derived substrain, were used in this study. *Pad4*^–/–^ and *Csf3*^–/–^ mice, both on a C57BL/6 genetic background, were procured from Cyagen (Suzhou) Biotechnology. All mice were maintained under specific pathogen–free conditions with controlled temperature, humidity, and a 12-hour light/dark cycle. Mice were deeply anesthetized with pentobarbital sodium (50 mg/kg, i.p.). Under deep anesthesia, euthanasia was performed by exsanguination, followed by harvesting of lung and other tissues. Death was confirmed by cessation of respiration.

### Genotyping.

All *Csf3^–/–^* and *Pad4^–/–^* mice were genotyped by PCR prior to use. Genomic DNA was extracted from toe tissue, and PCR was performed using specific primer sets. For *Csf3^–/–^* genotyping, the following primers were used: common forward primer 5′-CTCCATCCCTACTACCCATGATTG-3′, *Csf3^–/–^* reverse primer 5′-GTCATTCTGCCTGTCATTCTGCCG-3′, and WT reverse primer 5′-GTCCAGCATTCAGGCCGTTCTGTC-3′. For *Pad4^–/–^* genotyping, the primers were: common forward primer 5′-GTGAGAATGAGCCTCAAGAAGATC-3′, *Pad4^–/–^* reverse primer 5′-CTGTGATTCGTGTCTTCCAGTGC-3′, and WT reverse primer 5′-TTGGACCAGAGAGACCGCATTG-3′.

PCR thermal cycling conditions were as follows: 94°C for 3 minutes 45 seconds, followed by 35 cycles of denaturation at 94°C for 30 seconds, annealing at 60°C for 35 seconds, and extension at 72°C for 35 seconds, with a final extension at 72°C for 5 minutes. Amplified products were separated on a 1.5% agarose gel. For *Csf3*^–/–^ mice, a single 397 bp band indicated homozygosity; the presence of both 397 bp and 457 bp bands indicated heterozygosity; and a single 457 bp band indicated the WT genotype. For *Pad4^–/–^* mice, a 657 bp band indicated homozygosity; both 657 bp and 443 bp bands indicated heterozygosity; and a 443 bp band indicated the WT genotype. Water was used as a negative control and yielded no bands.

### Animals and genotyping.

Male C57BL/6 (WT) mice aged 6–8 weeks were obtained from the Laboratory Animal Center of Nanjing Medical University. *Pad4*^–/–^ and *Csf3*^–/–^ mice, both on a C57BL/6 genetic background, were procured from Cyagen (Suzhou) Biotechnology. All mice were maintained under specific pathogen–free conditions, with controlled temperature, humidity, and a 12-hour light/dark cycle. Genotyping was performed via PCR using genomic DNA extracted from toe biopsies. Distinct amplicon patterns identified WT, heterozygous, and homozygous knockouts (Supplemental Figure 4). Mice were euthanized humanely after experiments.

### Neutrophilia models.

To induce neutrophilia in mice, we used the pLIVE plasmid (Mirus Bio, USA) for in vivo protein expression. The cDNA sequence of *Csf3* (NCBI ID: 12985) was inserted into the pLIVE vector to construct the *Csf3* plasmid, as previously described (51). In total, 50 μg of the *Csf3* plasmid was diluted in 0.9% saline to a total volume equivalent to 10% of the mouse’s body weight and then administered i.v. via the tail vein over 5–8 seconds to establish a mouse model of neutrophilia.

### MPP model.

To establish an MPP model, mice were intratracheally injected once with 25 μL of either PBS or MP suspension. Samples were collected on the fourth day after injection (30, 52). To investigate the contribution of neutrophils to EC activation in MPP mice, we established MPP mouse models with varying neutrophil levels: (a) WT: WT + PBS, WT + MP; (b) neutropenic: *Csf3^–/–^* + PBS, *Csf3^–/–^* + MP; and (c) neutrophilic group: WT + vector + PBS, WT + *Csf3* plasmid + PBS, WT + Csf3 plasmid + MP. To further examine the role of NETs in EC activation in MPP mice, we generated mouse models with different NETs levels: (a) WT: WT + PBS, WT + MP; (b) NETs-deficient: *Pad4^–/–^* + PBS, *Pad4^–/–^* + MP; and (c) NETs-deficient but neutrophilic: *Pad4^–/–^* + vector + PBS, *Pad4^–/–^* + *Csf3* plasmid + PBS, *Pad4^–/–^* + *Csf3* plasmid + MP. Mice were selected and randomly assigned to each group, with 6 mice per group. Mice were randomly assigned to experimental groups (*n* = 6 per group). Animals that died prematurely before completing the experimental protocol were excluded from the analysis.

### NETs quantification.

NETs were quantified by measuring dsDNA and MPO-DNA levels in plasma and BALF samples from humans and mice. dsDNA levels were determined using the Quant-iT PicoGreen dsDNA kit (P11496, Invitrogen), while MPO-DNA complexes were quantified using an MPO-DNA ELISA kit (U96-1054E and U96-1089E, YOBIBIO), according to the manufacturers’ instructions.

### TLR2 antagonist treatment.

The TLR2 antagonist C29 (HY-100461, MedChem Express, USA) was dissolved in a vehicle comprising 10% dimethyl sulfoxide (DMSO), 40% polyethylene glycol (PEG) 300, 5% Tween 80, and 45% saline. A volume of vehicles alone was used for control treatments. All drug solutions were freshly prepared on the day of administration. Mice were i.p. injected with C29 (30 mg/kg) every other day for a total of 3 doses, starting 24 hours before MP intratracheal injection. Mice were randomly assigned to 4 groups: control, MP, C29, and MP + C29. The dosage and administration protocol were based on previously published data (53).

### Neutrophil isolation and stimulation.

Human neutrophils were isolated via density gradient centrifugation at 750*g* using a neutrophil separation kit (TBD Sciences, Tianjin, China), as previously reported (26). Cells (1 × 10^6^/mL) were stimulated with 1 × 10^8^ CFU/mL MP or PBS for 4 hours. Supernatants were collected for proteomics and cell pellets were collected for RNA-seq. NETs were induced using MP or 10% plasma from MP-associated thrombosis patients. Immunofluorescence analysis was conducted to evaluate NETs formation.

### Preparation of MP-induced NETs-enrich conditioned medium.

Neutrophils were stimulated for 4 hours at 37°C with 1 × 10^8^ CFU/mL MP. NETs-enrich conditioned medium was subsequently prepared as previously described (32). After centrifugation at 450*g* for 10 minutes, intact cells and debris formed a pellet, leaving a NETs-enrich supernatant. The dsDNA concentration in the conditioned medium was quantified using the Quant-iT PicoGreen dsDNA kit (P11496, Invitrogen) according to the manufacturer’s instructions.

To assess the contribution of MP-induced NETs to EC activation, Cl (200 μM; HY-100574, MedChem Express), a pharmacological inhibitor of NETs formation, was used to block NETs release. Neutrophils were assigned to 4 experimental groups: (a) untreated control, (b) MP alone, (c) Cl alone, and (d) MP + Cl. Cells in the Cl and MP + Cl groups were preincubated with Cl for 1 hour prior to MP stimulation. Conditioned supernatants from each group were then normalized to a dsDNA concentration of 400 ng/mL before being applied to HUVECs.

### HUVECs culture and simulation.

HUVECs were obtained from Bena Culture Collection (China) and maintained in EC medium (ScienCell 1001, USA) supplemented with 5% fetal bovine serum, 1% EC growth supplement, and 1% penicillin/streptomycin solution at 37°C in a humidified atmosphere with 5% CO_2_. To investigate the downstream signaling pathways mediating NETs-induced endothelial activation, HUVECs were separately pretreated with the TLR2 inhibitor C29 (50 μM; HY-100461, MedChem Express) for 1 hour, the MyD88 inhibitor TJ (15 μM; HY-139397, MedChem Express) for 1 hour, or the JAK1 inhibitor UPA (1 μM; HY-19569, MedChem Express) for 2 hours, followed by stimulation with MP-induced NETs (400 ng/mL dsDNA) for 24 hours. Cell viability was assessed using a Cell Counting Kit-8 (C0041, Beyotime) assay to exclude potential cytotoxic effects of the inhibitors. Experimental groups for each inhibitor study included control, NETs, inhibitor alone, and NETs + inhibitor. After incubation, cells were collected for Western blotting or immunofluorescence analysis.

### RNA-seq and bioinformatic analysis.

Nucleic acid extraction, library preparation, and RNA-seq of neutrophils were conducted following the methodology described in our previous study (38). DEGs were identified based on a fold change > 1.5 and an adjusted *P* < 0.05 using DESeq2 analysis. GO and KEGG pathway enrichment analyses were performed using the clusterProfiler package. GSVA of neutrophils was conducted for gene sets associated with neutrophil chemotactic activation (GO:0042119), chemotaxis (GO:0030593), migration (GO:1990266), degranulation (GO:0043312), and NETs (54). For HUVECs, GSVA and GSEA were performed using the 50 hallmark gene sets from the Molecular Signature Database. GSVA scores were compared using the limma package in R.

### PPI network analysis.

TLR2- and MP-NETs–associated proteins were analyzed using STRING (v10.5), applying a minimum interaction score of 0.7 to generate PPI networks.

### Immunofluorescence staining.

Cells or tissues were permeabilized with 0.3% Triton X-100 for 10 minutes and then blocked in 5% goat serum for 1 hour. After blocking, the samples were incubated with primary antibodies overnight at 4°C. The following day, they were washed 3 times with PBS and incubated with the appropriate secondary antibodies for 1 hour at room temperature. Nuclei were counterstained with DAPI solution for 5 minutes. Immunofluorescence signals were visualized using a laser confocal microscope (ZEISS, Germany).

The primary antibodies used included: anti-MPO (1:200, 66177-1-Ig, Proteintech), anti-CitH3 (1:100, ab5103, Abcam), anti-ICAM-1 (1:200, 60299-1-Ig, Proteintech; 1:250, ab171123, Abcam), anti-VCAM-1 (1:250, ab134047, Abcam), anti-P-selectin (1:100, 60322-1-Ig, Proteintech; 1:100, ab255822, Abcam), anti-vWF (1:250, ab154193, Abcam; 1:100, GB11020, Servicebio), and anti-CD31 (1:200, ab56299, Abcam). The secondary antibodies were Alexa Fluor Plus 488-conjugated goat anti-rabbit IgG (1:500, Thermo Fisher, A32731), Alexa Fluor Plus 488-conjugated goat anti-mouse IgG (1:500, Thermo Fisher, A32723), and Alexa Fluor Plus 647-conjugated goat anti-rat IgG (1:500, Thermo Fisher, A48265). For negative controls, tissue sections were incubated with secondary antibodies alone in the absence of primary antibodies under the same experimental conditions. No detectable nonspecific fluorescence was observed in the secondary antibody-only controls (Supplemental Figure 10).

### Histopathology.

The left lung tissues were fixed in 4% paraformaldehyde, embedded in paraffin, sectioned, and stained with H&E for histological examination. For semi-quantitative analysis, 4 random fields per section were assessed to determine histopathological changes. Lung injury was scored based on alveolar edema, alveolar wall thickness, tissue destruction, and inflammatory cell infiltration, using a 4-point scale: 0 = normal, 1 = mild, 2 = moderate, and 3 = severe. The individual scores were summed to generate an overall lung injury score, as previously described (55).

### TAT complex quantification.

Plasma levels of the TAT complex were quantified using enzyme-linked immunosorbent assay kits (E-EL-M1138, Elabscience, China) according to the manufacturer’s instructions.

### BALF analysis.

BALF was collected by infusing 500 μL of PBS into the right lung via a tracheal cannula. BALF cells were pelleted by centrifugation at 4°C at 1,000*g* for 5 minutes. Total cell counts were determined using cell counting plates, and differential cell analysis was performed by flow cytometry. For staining, the cell suspension was incubated with allophycocyanin-conjugated (APC-conjugated) anti-mouse CD45 (1032112, BioLegend) and fluorescein isothiocyanate–conjugated (FITC-conjugated) anti-mouse Ly6G (127606, BioLegend) at 4°C for 30 minutes. The stained cells were analyzed using a flow cytometer (Beckman, USA). Data were processed using FlowJo software (v10.8.1).

### Western blot analysis.

Western blot analysis was performed to evaluate protein expression in cultured cells and lung tissues, as previously described (56). Total cellular proteins were extracted using RIPA lysis buffer. For analysis of membrane-associated adhesion molecules, membrane proteins were additionally extracted from HUVECs using a Plasma membrane Protein and Cytoplasmic Protein Extraction Kit (P0033, Beyotime Biotechnology) according to the manufacturer’s instructions. The primary antibodies used included: anti-TLR2 (1:500, ET1705-92, Huabio), anti-MyD88 (1:2,000, 67969-1-Ig, Proteintech), anti-JAK1 (1:1,000, 3344, CST), anti-phospho-JAK1 (Tyr1034/1035) (1:1,000, 74129, CST), anti-JAK2 (1:1,000, ET1607-35, Huabio), anti-phospho-JAK2 (Tyr1007) (1:1,000, ab195055, Abcam), anti-STAT3 (1:1,000, ET1605-45, Huabio), anti-phospho-STAT3 (Ser727) (1:500, ET1607-39, Huabio), anti-ICAM-1 (1:1,000, 60299-1-Ig, Proteintech), anti-VCAM-1 (1:500, ab134047, Abcam), anti-P-selectin (1:1,000, ET1703-49, Huabio), anti-vWF (1:1,000, ET1701-87, Huabio), and anti-CitH3 (1:1,000, ab5103, Abcam).

### Membrane protein extraction.

Membrane proteins were extracted from HUVECs using a Plasma Membrane Protein and Cytoplasmic Protein Extraction Kit (P0033, Beyotime Biotechnology, China) according to the manufacturer’s instructions. Briefly, cells were collected and lysed sequentially with cytoplasmic and membrane protein extraction buffers. After centrifugation at 14,000*g*, the membrane protein fraction was isolated and quantified using a BCA protein assay kit prior to Western blot analysis.

### Scanning electron microscopy.

Fresh thrombus samples were fixed in 2.5% glutaraldehyde and then processed through standard dehydration and sputter coating procedures for imaging. The prepared samples were examined for ultrastructural features using a scanning electron microscope (Hitachi SU8100) operated at an accelerating voltage of 3.0 kV.

### Statistics.

Statistical analyses were performed using GraphPad Prism 9.0, R 4.5.1, and SPSS 26. Missing data were addressed using multiple imputation by chained equations (*mice* package in R). Patients lost to follow-up were treated as right-censored in Cox proportional hazards models, in accordance with standard survival analysis methodology. Data are presented as mean ± SD or median (IQR). Group comparisons were performed using 2-tailed Student’s *t* test, Mann-Whitney *U* test, or 1-way ANOVA followed by Games-Howell post hoc test. Categorical variables were analyzed using χ² tests. Propensity score matching was performed using age and sex to balance baseline characteristics between groups. In the matched cohort, LASSO regression was applied to select relevant laboratory indicators. Given the limited number of outcome events, Firth’s penalized logistic regression was subsequently used to evaluate associations between selected variables and the outcome. The discriminative ability of neutrophil count was assessed using ROC analysis with internal validation by bootstrap resampling. Kaplan-Meier analysis evaluated thrombosis risk within 60 days, with neutrophil thresholds defined using X-tile software (version 3.6.1). Internal sensitivity analyses were performed by modeling neutrophil count as a continuous variable in Cox regression and by stratifying patients into neutrophil quartiles. A *P* value less than 0.05 was considered statistically significant.

### Study approval.

This study was conducted in accordance with the principles of the Declaration of Helsinki and approved by the institutional Ethics Committee (nos. 201812257 and 201812257-1). Written informed consent was obtained from all participants or their legal guardians. All animal experiments were conducted following the Regulations for the Administration of Affairs Concerning Experimental Animals and were approved by the IACUC (1905057 and 1905057-1).

### Data availability.

Source data underlying all graphs, uncropped Western blot images, deidentified clinical cohort data, and processed RNA-seq gene expression count matrices are provided in the Supporting Data Values file accompanying this manuscript. The raw RNA-seq data generated in this study have been deposited in the Genome Sequence Archive for Human (GSA-Human) at the National Genomics Data Center, China National Center for Bioinformation/Beijing Institute of Genomics, Chinese Academy of Sciences. The accession information is publicly available under BioProject accession no. PRJCA037579 and GSA-Human accession nos. HRA010858 and HRA010859 (https://ngdc.cncb.ac.cn/gsa-human). Due to ethical restrictions and patient consent agreements, access to individual-level sequencing data is controlled. The proteomic data generated in this study have been deposited in OMIX, China National Center for Bioinformation/Beijing Institute of Genomics, Chinese Academy of Sciences (https://ngdc.cncb.ac.cn/omix/release/OMIX009662).

## Author contributions

XH and YZ performed the experiments, processed the experimental data, performed most of the analysis, drafted the manuscript, and designed the figures. YG performed the preprocessing of the data and aided in the data analysis. TL, HG, YL, DL, and HS were involved in processing the raw samples and in the ELISA experiment. XH and FL secured project funding. FL and DZ conceived the project and act as guarantor for this study.

## Conflict of Interest

The authors have declared that no conflict of interest exists.

## Funding support

- National Natural Science Foundation of China (82500006 to XH).
- National Natural Science Foundation of China (82572609 to FL).
- Children’s Hospital of Nanjing Medical University (KJCXM2024002 to FL).

## Supplementary Material

Supplemental data

Unedited blot and gel images

Supporting data values

## Figures and Tables

**Table 1:**
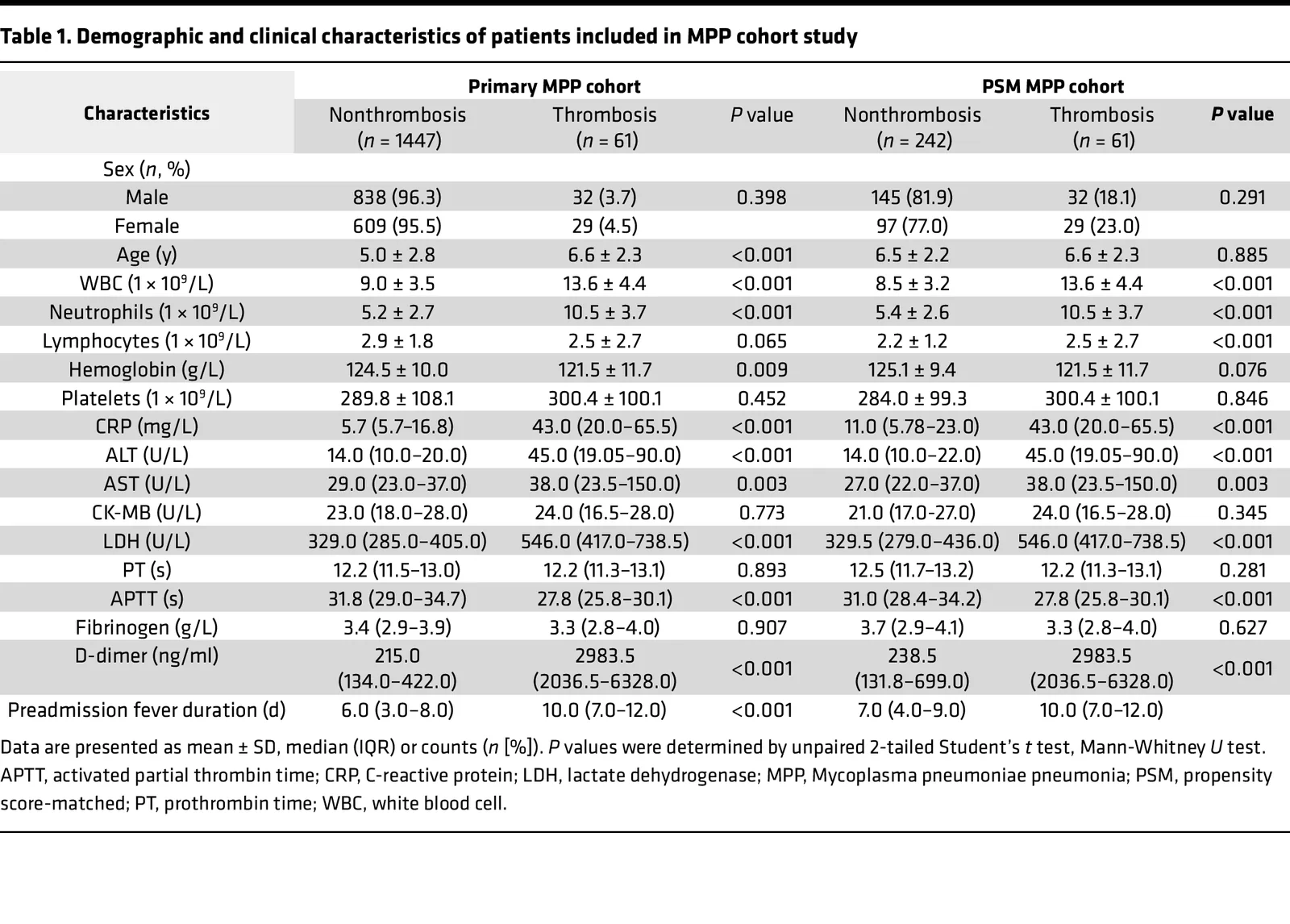
Demographic and clinical characteristics of patients included in MPP cohort study

## Version 1 09/22/2026

Electronic publication
