# Supplementary material for: Neutrophils promote endothelial cell activation in pediatric Mycoplasma pneumoniae pneumonia
Source: JCI Insight. 2026 Sep 22;11(18):e203856. doi: 10.1172/jci.insight.203856 (PMC13596722; doi:10.1172/jci.insight.203856)
Supplement: Supplementary file 1 [file jciinsight-11-203856-s188.pdf]

Supplemental data

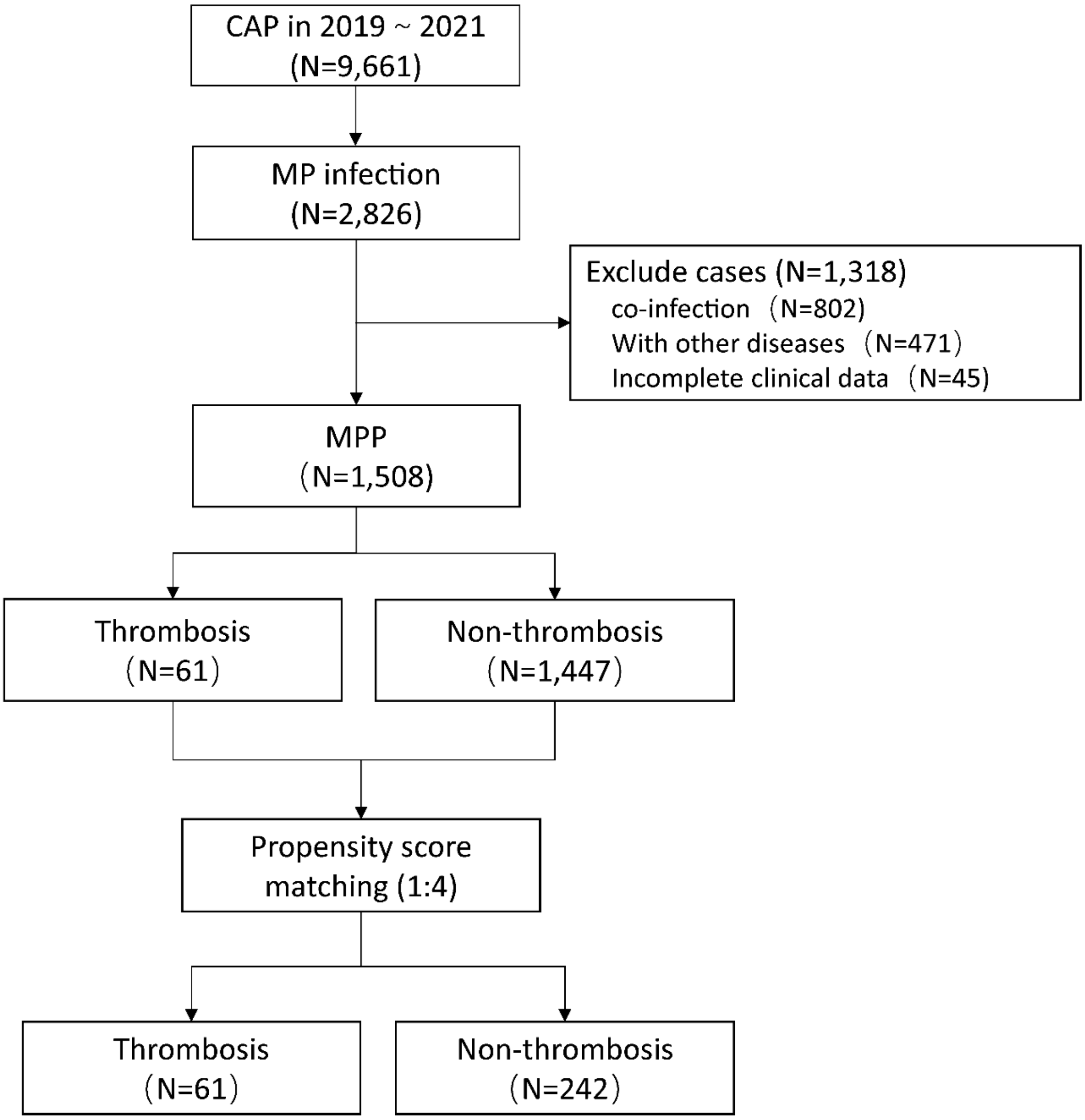

Supplemental Figure 1. Flowchart of the study.
Abbreviations: CAP, community-acquired pneumonia; MP, Mycoplasma pneumoniae; MPP, *Mycoplasma pneumoniae* pneumonia.

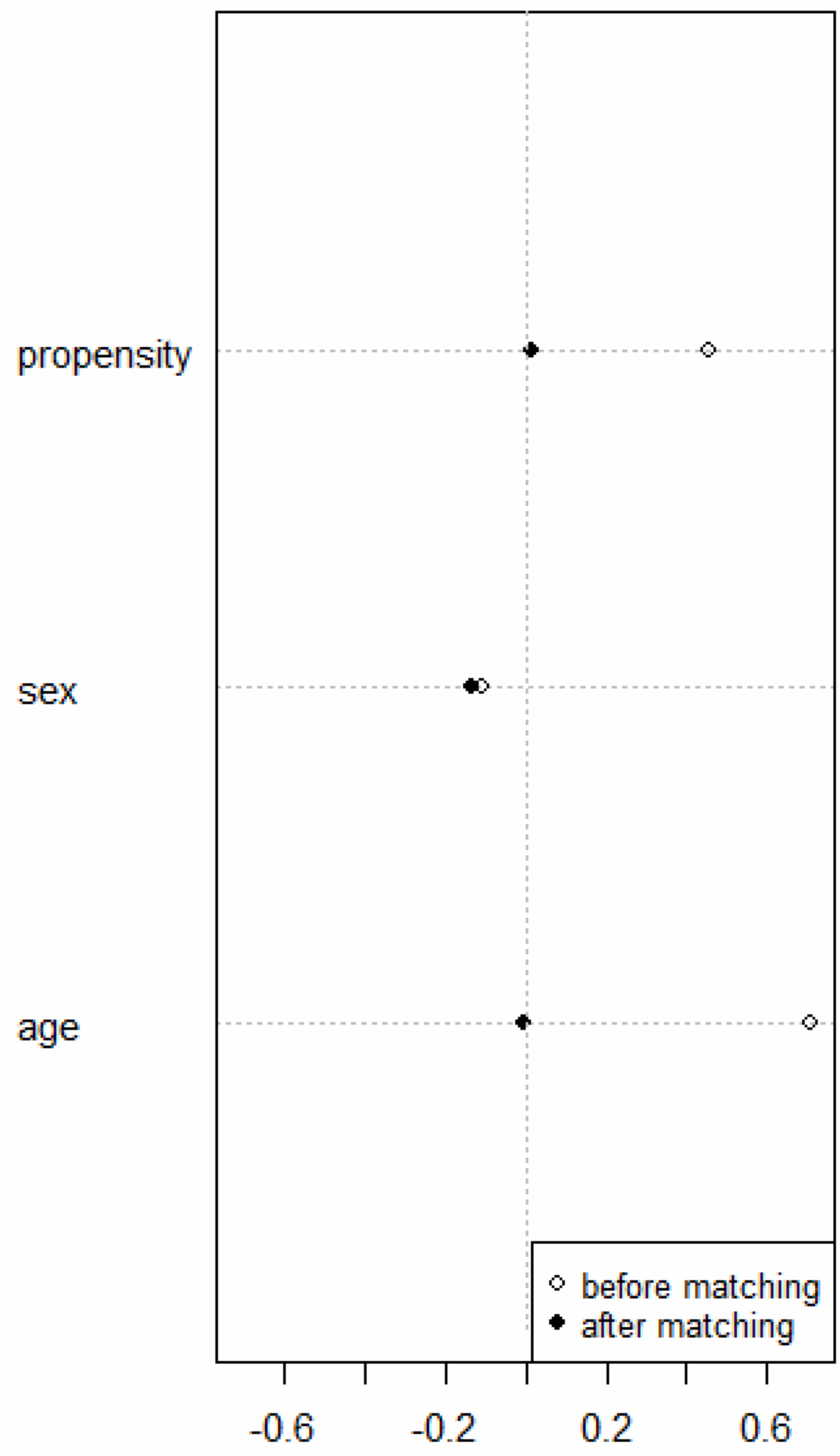

Supplemental Figure 2. Standardized mean difference before and after propensity score matching.

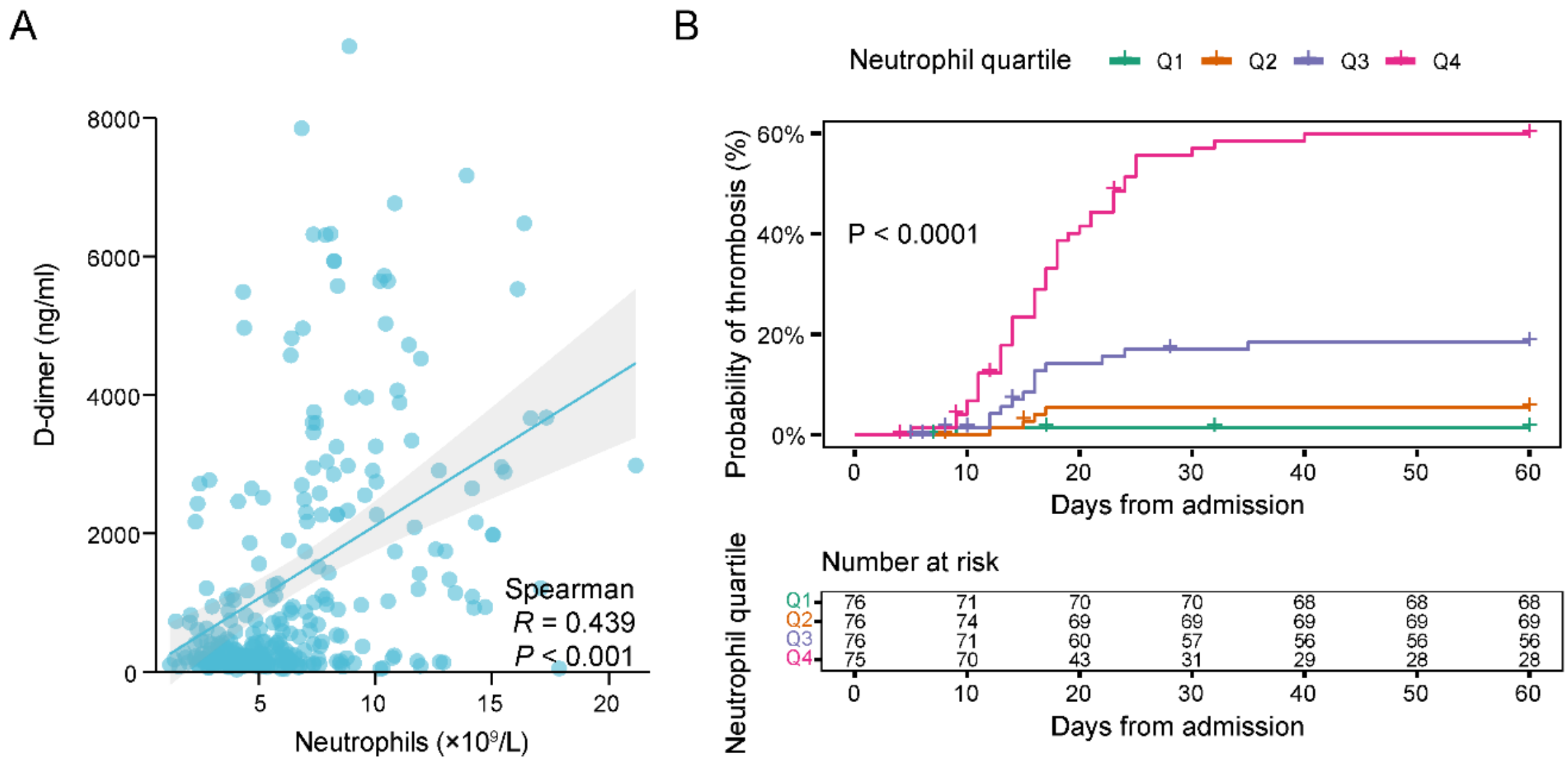

Supplemental Figure 3. Internal sensitivity analyses supporting the association between neutrophil count and thrombosis risk in the propensity score–matched (PSM) *Mycoplasma pneumoniae* pneumonia (MPP) cohort.

(A) Correlation between neutrophil count and D-dimer levels. A moderate positive correlation was observed (Spearman's r = 0.439, P < 0.001).

(B) Kaplan–Meier analysis of cumulative thrombosis incidence stratified by neutrophil count quartiles (Q1–Q4). Differences among groups were assessed using the log-rank test ($P < 0.001$).

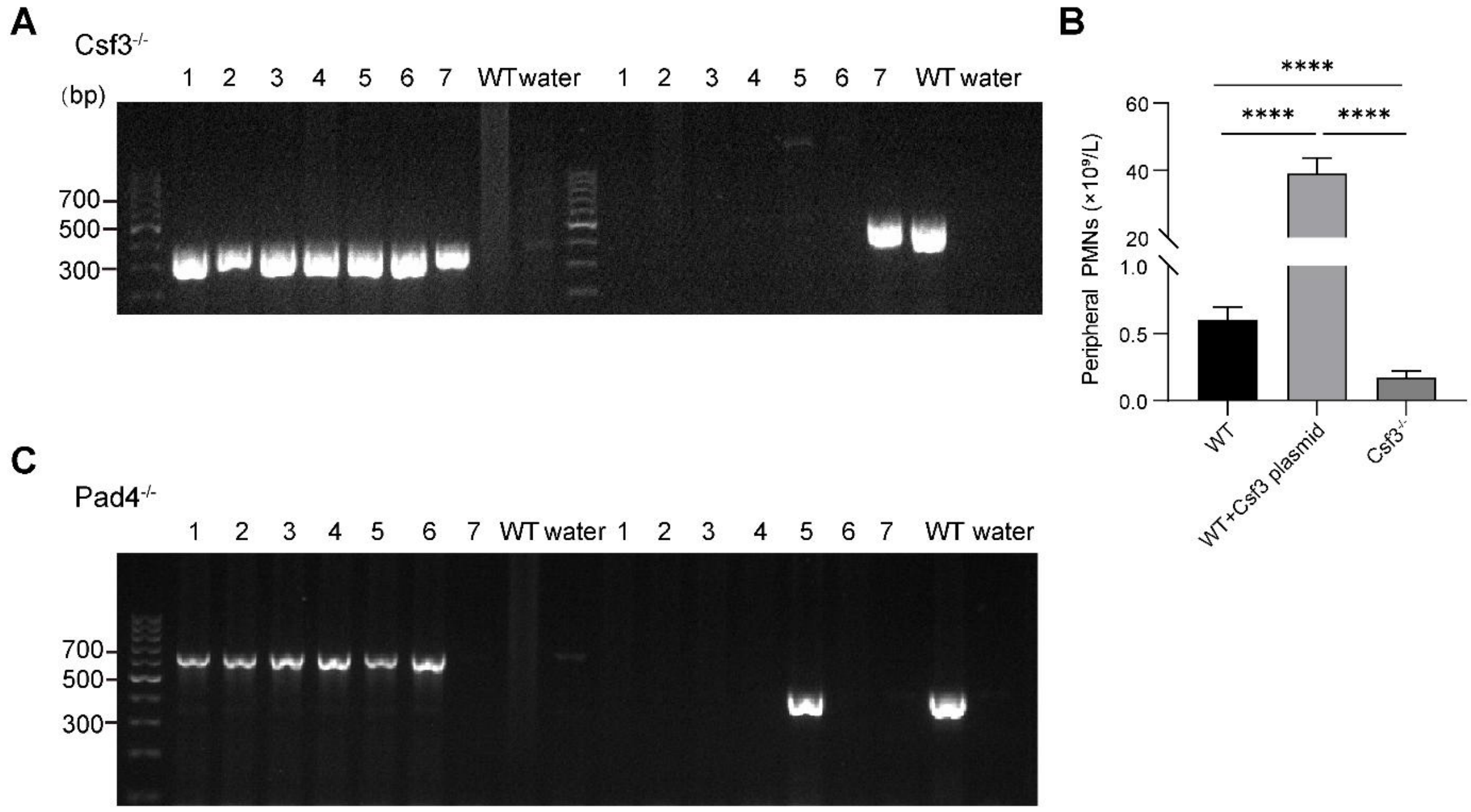

Supplemental Figure 4. Identification of mouse genotypes by agarose gel electrophoresis

(A) *Csf3*$^{-/-}$ genotyping. Representative agarose gel electrophoresis results are shown. DNA electrophoresis results show that homozygous *Csf3*$^{-/-}$ mice (#1–#6) display a single band at 397 bp. Mouse #7 is a *Csf3*$^{+/-}$ heterozygote, exhibiting two bands at 397 bp and 457 bp. Wild type (WT) mice show a single band at 457 bp. Water was used as a negative control, with no bands observed.

(B) Peripheral blood neutrophil counts in WT mice administered *Csf3* plasmid and *Csf3*$^{-/-}$ mice. Tail vein injection of the *Csf3* plasmid was used to induce a neutrophilia model. Quantitative data are presented as mean ± SD and were analyzed by one-way ANOVA followed by the Games–Howell post-hoc test.

(C) *Pad4*$^{-/-}$ genotyping. Representative agarose gel electrophoresis results are shown. DNA electrophoresis results show that homozygous *Pad4*$^{-/-}$ mice (#1–#4, #6) exhibit a single band at 657 bp. Mouse #5 is a Pad4$^{+/-}$ heterozygote, displaying bands at 657 bp and 443 bp. WT mice display a single band at 457 bp. Water served as the negative control and showed no bands.

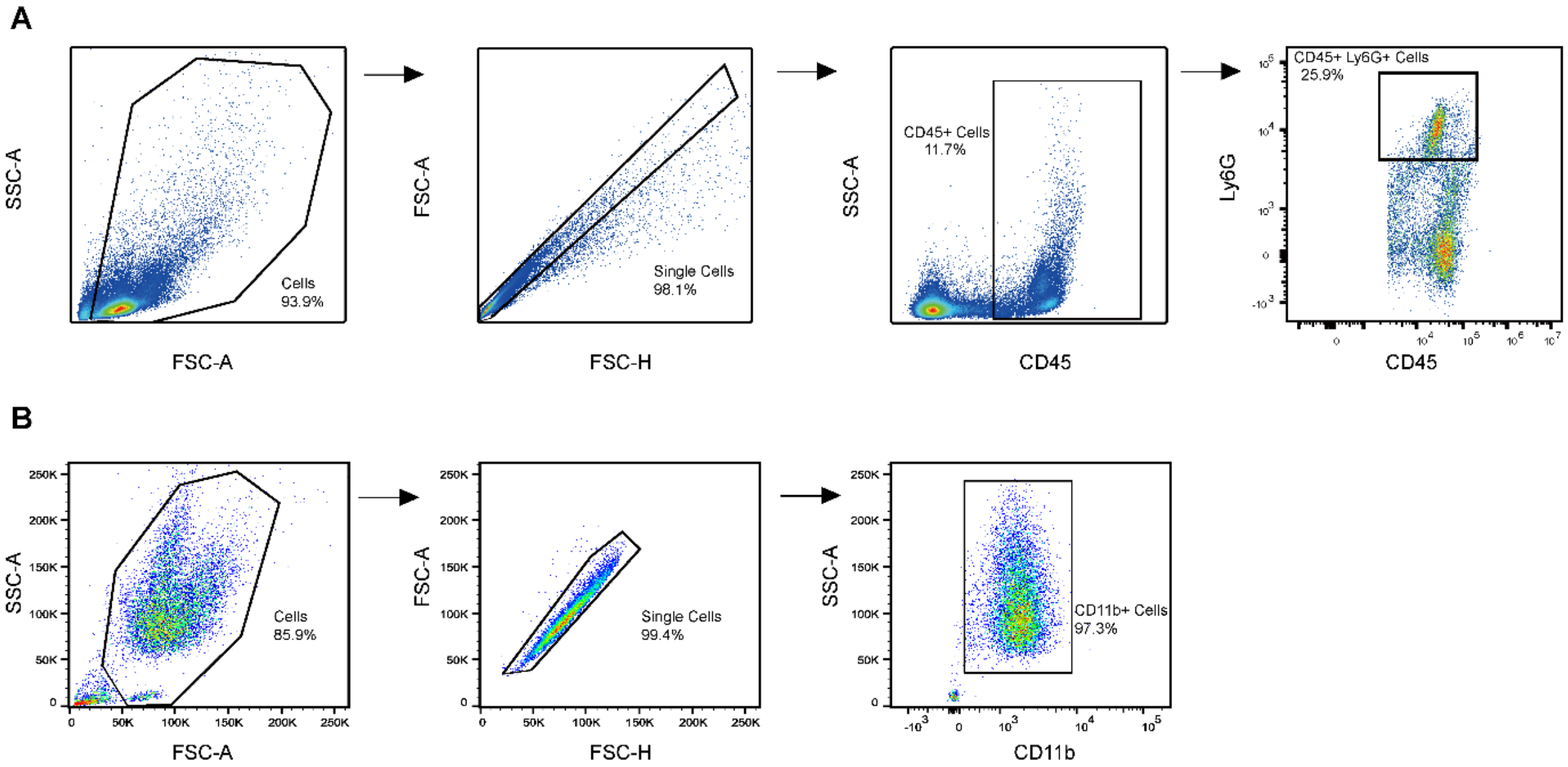

Supplemental Figure 5. Gating strategy for flow cytometry.

(A) Classification of immune cells in BALF. $CD45^{+}ly6G^{+}$ cells: mouse neutrophils.

(B) Purity assessment of isolated human primary neutrophils. $CD11b^{+}$ cells: human neutrophils.

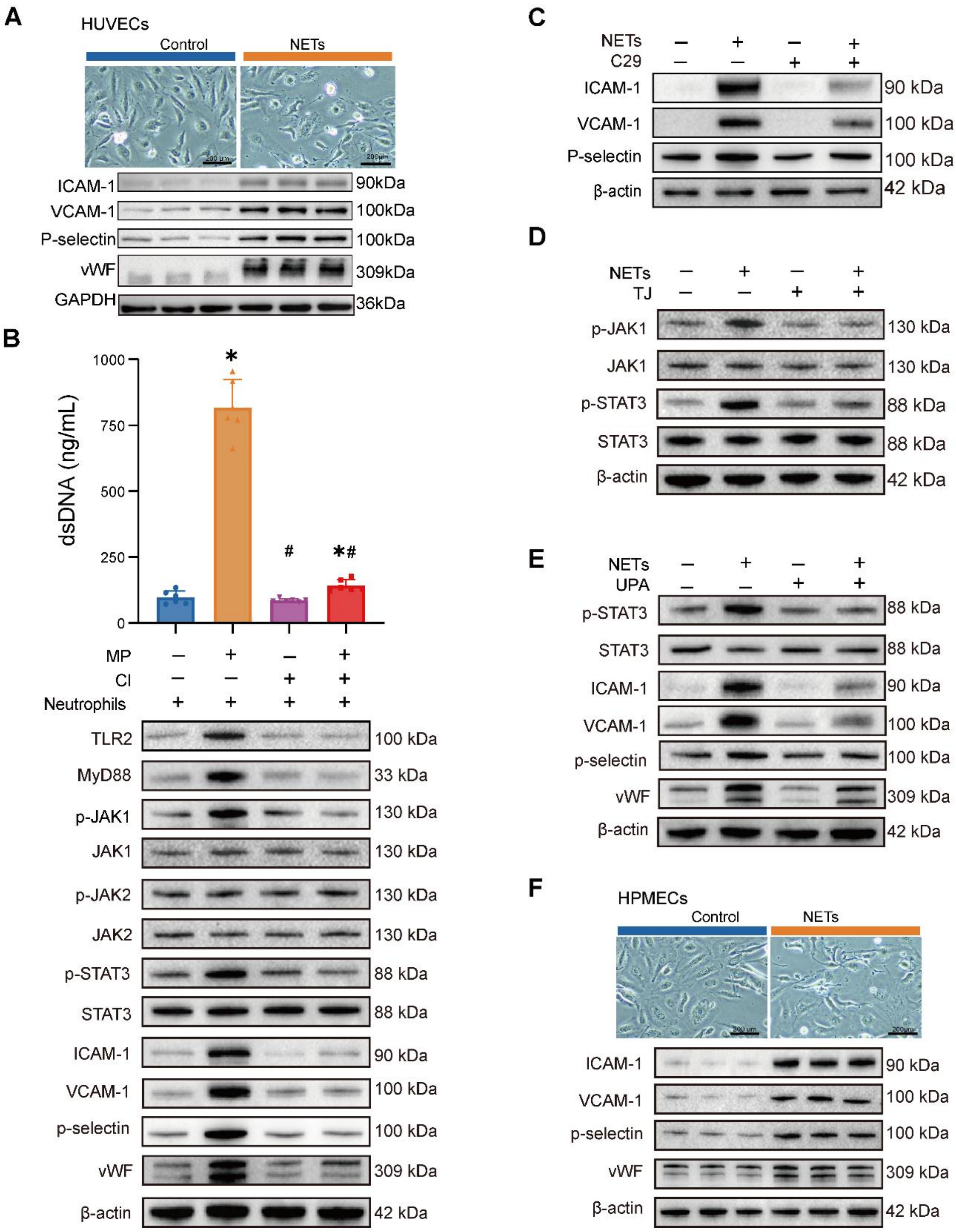

Supplemental Figure 6. NETs promote endothelial activation via TLR2 (n = 3 independent experiments).

(A) Representative images of human umbilical vein endothelial cells (HUVECs) cultured with or without MP-induced NETs. Scale bar = 200 μm. EC activation markers (ICAM-1, VCAM-1, P-selectin, and vWF) were assessed by Western blotting.

(B) Neutrophils were stimulated with MP to generate NETs-rich conditioned medium, with or without Cl-amidine pretreatment to inhibit NETs formation. Conditioned medium was subsequently applied to HUVECs. Expression of TLR2, MyD88, JAK-STAT3 signaling

proteins, and EC activation markers was assessed by Western blotting. Quantitative data are presented as mean ± SD and were analyzed by one-way ANOVA followed by the Games–Howell post-hoc test. *$P < 0.05$ vs. neutrophils group; #$P < 0.05$ vs. neutrophils + MP group.

(C) Membrane proteins from HUVECs treated with NETs and/or the TLR2 inhibitor C29 were analyzed by Western blot.

(D) HUVECs were pretreated with the MyD88 inhibitor TJ-M2010-5 (TJ) before stimulation with MP-induced NETs. Representative Western blot images of JAK-STAT3 signaling proteins are shown.

(E) HUVECs were pretreated with the JAK1 inhibitor upadacitinib (UPA) before stimulation with MP-induced NETs. Representative Western blot images of JAK-STAT3 signaling proteins and EC activation markers are shown.

(F) Representative images of human pulmonary microvascular endothelial cells (HPMECs) cultured with or without MP-induced NETs. Scale bar = 200 μm. Representative Western blot images of EC activation markers are shown.

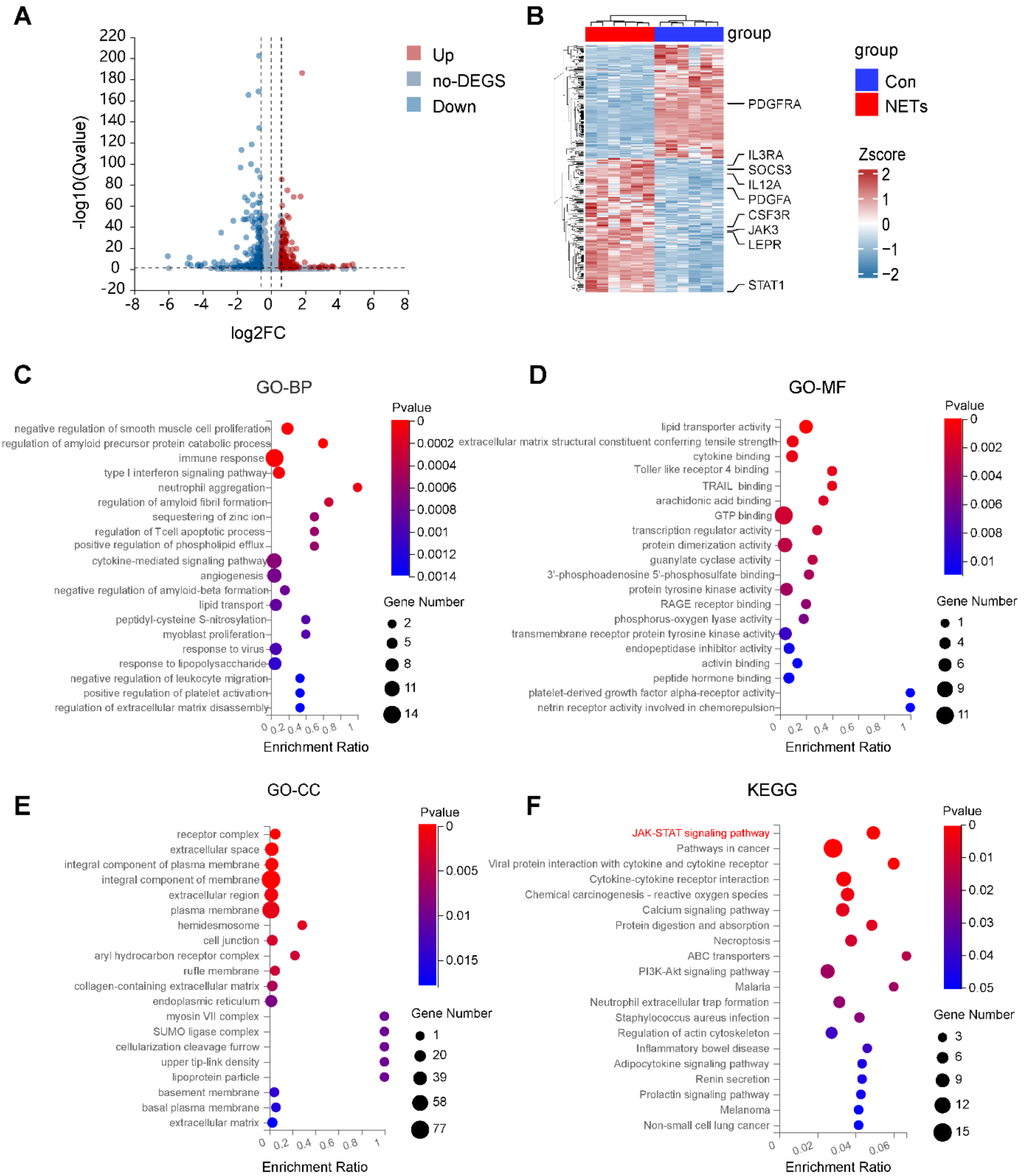

Supplemental Figure 7. RNA sequencing analysis of NET-treated human umbilical vein endothelial cells (HUVECs).

(A) HUVECs cultured with or without NETs (400 ng/mL) were subjected to RNA sequencing. The volcano plot displays the differentially expressed genes (DEGs) between the NETs and control groups. Red dots represent upregulated genes, and blue dots represent downregulated genes. DEGs were identified using DESeq2 analysis. RNA sequencing was performed using six biological replicates per group.

(B) Heatmap showing the expression profiles of DEGs. Red indicates upregulation and blue indicates downregulation. Genes associated with the JAK–STAT signaling pathway are highlighted.

(C-F) Enrichment analysis was performed using Gene Ontology (GO) and Kyoto Encyclopedia of Genes and Genomes (KEGG) databases.

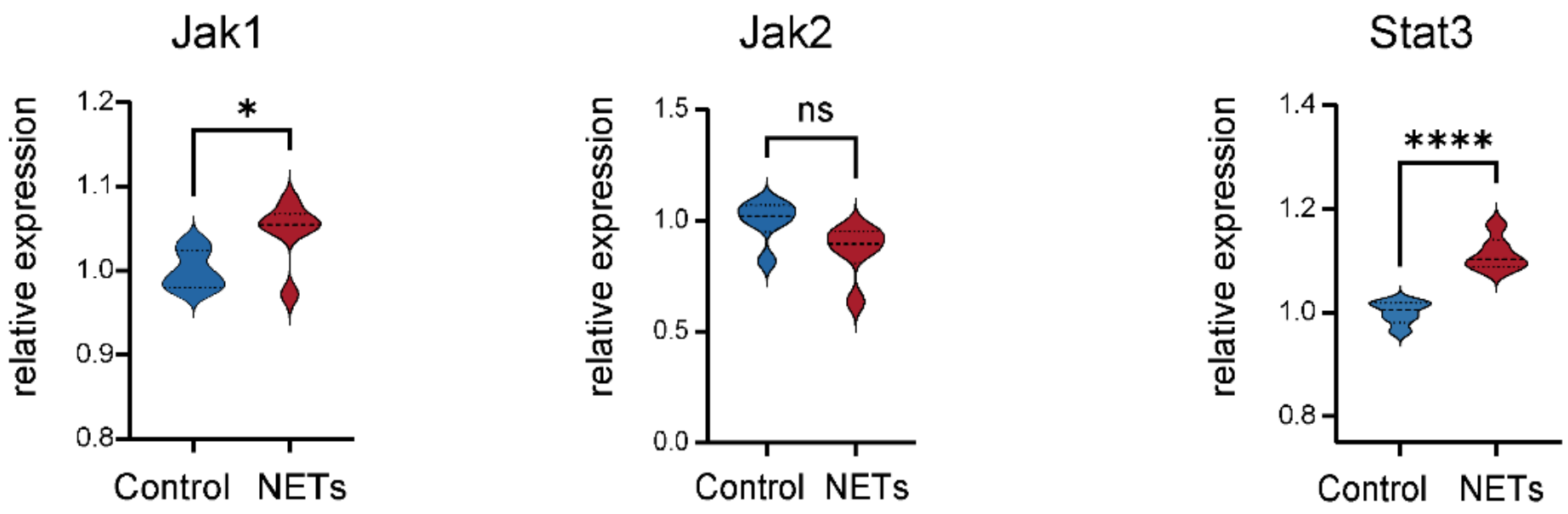

Supplemental Figure 8. Expression levels of key genes involved in the JAK–STAT3 signaling pathway.

Quantitative analysis of key genes involved in the JAK–STAT3 signaling pathway in NET-treated and control HUVECs based on RNA-sequencing data. Data were obtained from n = 6 biological replicates per group. Data are presented as mean ± SD. Statistical comparisons were performed using an unpaired two-tailed Student's t test. *$P < 0.05$, **$P < 0.01$, ***$P < 0.001$, **$P < 0.0001$.

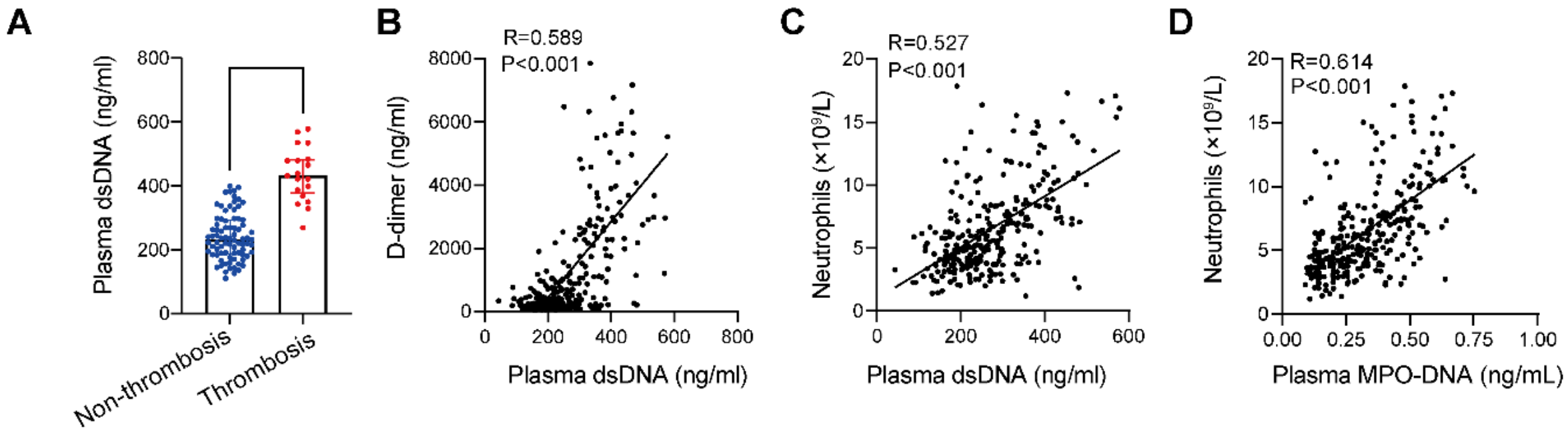

Supplemental Figure 9. Plasma NETs levels and their correlation with coagulation and neutrophil counts in MPP patients (*n*=303).

(A) Plasma levels of dsDNA in the PSM MPP cohort. Data are presented as median with interquartile range and were analyzed using the Mann-Whitney U test.

(B–D) Correlation analyses between plasma NETs markers and coagulation or neutrophil counts. Plasma dsDNA positively correlated with (B) D-dimer levels and (C) peripheral neutrophil counts. (D) Plasma MPO-DNA complexes also positively correlation with neutrophil counts. Spearman's rank correlation analysis was performed.

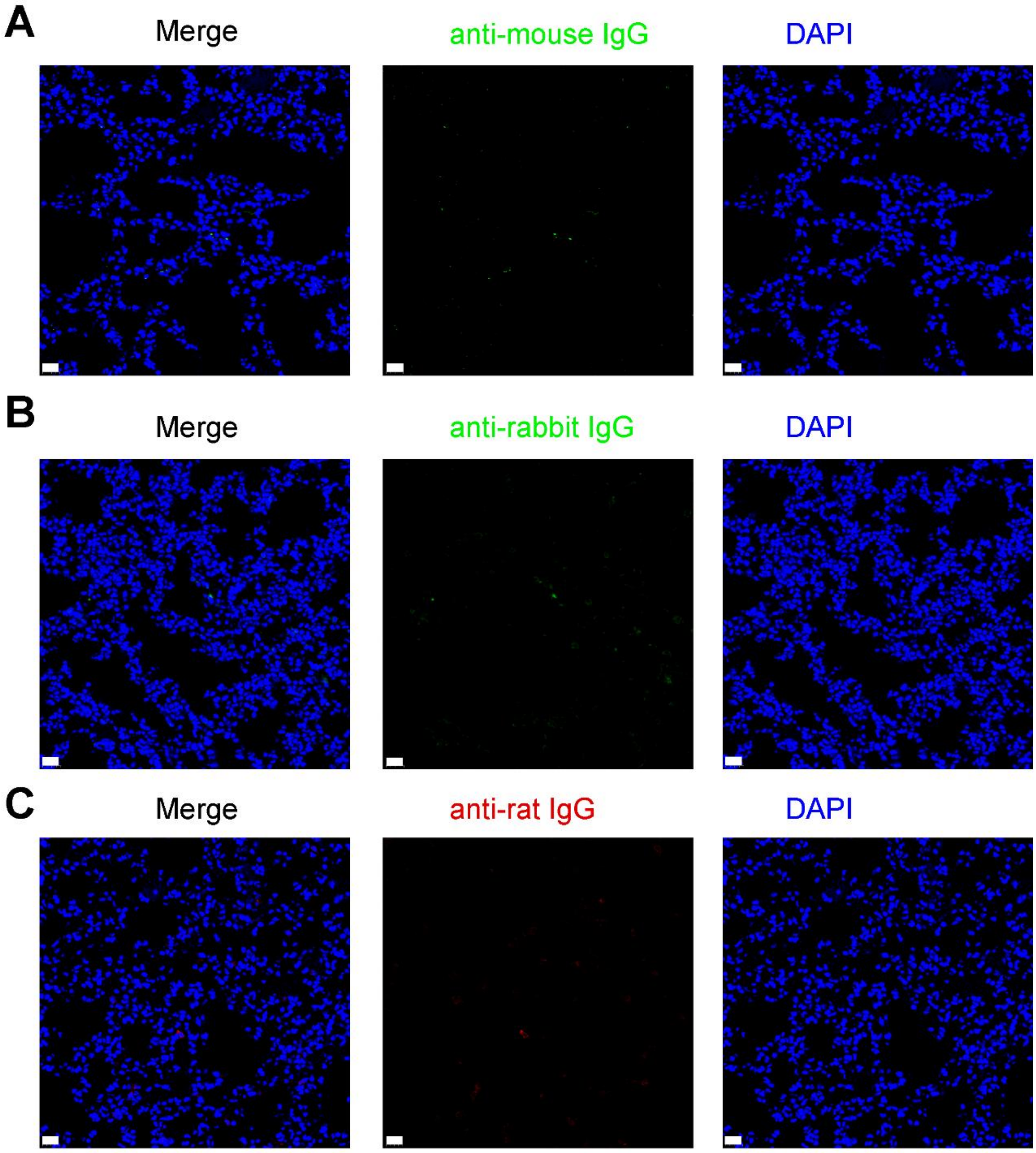

Supplementary Figure 10. Secondary antibody–only controls for immunofluorescence staining.

Secondary antibody alone controls. Lung sections were incubated with (A) anti-mouse IgG, (B) anti-rabbit IgG, and (C) anti-rat IgG in the absence of primary antibodies. DAPI was used for nuclear counterstaining. No detectable nonspecific fluorescence was observed. Scale bars = 20 μm.

Supplemental Table 1. Coefficients of laboratory variables selected by LASSO regression (corresponding to Figure 1G)

Variable	Coefficient
(Intercept)	-5.05106
WBC	0.043054
Neutrophils	0.249207
CRP	0.018776
ALT	0.0013
LDH	0.000934
PT	-0.03727
APTT	-0.01848
D dimer	0.000214
Fibrinogen	-0.0273
Pre-admission fever duration	0.109889

Variables were selected using LASSO regression in the propensity score–matched cohort. Coefficients represent penalized estimates.

Supplemental Table 2. Internal sensitivity analyses of neutrophil count as a predictor of thrombosis risk.

Analysis type	Variable	HR	95% CI	P value	Notes
Kaplan–Meier / log-rank	Neutrophil ≥8.82 vs <8.82 $\times 10^9$/L	-	-	<0.001	X-tile cutoff
Cox univariate	Neutrophil (continuous, $\times 10^9$/L)	1.383	1.315 – 1.454	<0.001	Internal sensitivity analysis
Cox multivariate	Neutrophil (continuous, $\times 10^9$/L)	1.344	1.272 – 1.421	<0.001	Adjusted for age, sex, and D-dimer

Abbreviations: HR= hazard ratio; CI = confidence interval

Supplemental Table 3. MP-NETs related proteins.

Genes	First.Protein.Description	/hwfsyt2/SP_MSI/CommercialProjects/2024/DIA_Label_free/20241202_F24A610003447_J241125043_hsa_Astral_gs/mzml//24NP23260001.mzML
MPO	Myeloperoxidase	1.81E+07
AZU1	Azurocidin	1.60E+07
PRSS1	Serine protease 1	1.17E+07
S100A8	Protein S100-A8	1.09E+07
LYZ	Lysozyme C	1.01E+07
S100A12	Protein S100-A12	1.00E+07
S100A9	Protein S100-A9	7.74E+06
LCN2	Neutrophil gelatinase-associated lipocalin	7.50E+06
PRTN3	Myeloblastin	6.84E+06
CTSG	Cathepsin G	6.25E+06
CALM1	Calmodulin-1	4.18E+06
SH3BGRL3	SH3 domain-binding glutamic acid-rich-like protein 3	3.94E+06
CAT	Catalase	3.29E+06
ANXA3	Annexin A3	2.67E+06
CAP1	Adenylyl cyclase-associated protein 1	2.34E+06
KRT1	Keratin, type II cytoskeletal 1	2.19E+06
ELANE	Neutrophil elastase	2.16E+06
TALDO1	Transaldolase	2.13E+06
KRT10	Keratin, type I cytoskeletal 10	1.71E+06
LTF	Lactotransferrin	1.39E+06

SH3BGRL	SH3 domain-binding glutamic acid-rich-like protein	1.37E+06
ANXA5	Annexin A5	1.33E+06
PPIA	Peptidyl-prolyl cis-trans isomerase A	1.32E+06
HP	Haptoglobin	1.32E+06
S100A6	Protein S100-A6	1.18E+06
LCP1	Plastin-2	1.17E+06
MSN	Moesin	1.13E+06
RNASE2	Non-secretory ribonuclease	1.12E+06
PPIB	Peptidyl-prolyl cis-trans isomerase B	1.11E+06
TMSB4X	Thymosin beta-4	1.07E+06
ALDOA	Fructose-bisphosphate aldolase A	1.02E+06
HSPA6; HSPA7	Heat shock 70 kDa protein 6	980305
GYG1	Glycogenin-1	959892
FLNA	Filamin-A	946630
APEX1	DNA-(apurinic or apyrimidinic site) endonuclease	943579
ACTN1	Alpha-actinin-1	896889
BASP1	Brain acid soluble protein 1	866573
RPS27A; UBA52; UBB; UBC	Polyubiquitin-B	842976
PLBD1	Phospholipase B-like 1	779247
MYH9	Myosin-9	732108
ANP32A	Acidic leucine-rich nuclear	688299

	phosphoprotein 32 family member A	
ARHGDIB	Rho GDP-dissociation inhibitor 2	680367
HSPA1A	Heat shock 70 kDa protein 1A	667538
HSPA8	Heat shock cognate 71 kDa protein	657184
SERPINC1	Antithrombin-III	632326
TCN1	Transcobalamin-1	560304
ANXA1	Annexin A1	529053
CTSS	Cathepsin S	518981
PARK7	Parkinson disease protein 7	511974
IQGAP1	Ras GTPase-activating-like protein IQGAP1	497864
ACTB; ACTG1	Actin, cytoplasmic 1	493483
OLFM4	Olfactomedin-4	492142
HSPA5	Endoplasmic reticulum chaperone BiP	445457
KLK11	Kallikrein-11	439482
NCF2	Neutrophil cytosol factor 2	439055
ZYX	Zyxin	427912
A2M	Alpha-2-macroglobulin	387788
VCL	Vinculin	377855
RAB7A	Ras-related protein Rab-7a	375332
GDI2	Rab GDP dissociation inhibitor beta	357539
COL9A1	Collagen alpha-1(IX) chain	342647
WIPF1	WAS/WASL-interacting protein family member 1	336180
MMP9	Matrix metalloproteinase-9	328138

S100A4	Protein S100-A4	321626
ITGB2	Integrin beta-2	310055
DBI	Acyl-CoA-binding protein	308057
GPI	Glucose-6-phosphate isomerase	304843
WDR1	WD repeat-containing protein 1	298930
CHIT1	Chitotriosidase-1	297843
ANXA6	Annexin A6	297419
RNASE3	Eosinophil cationic protein	294989
LMNB1	Lamin-B1	291988
LSP1	Lymphocyte-specific protein 1	280572
COTL1	Coactosin-like protein	273370
PSTPIP1	Proline-serine-threonine phosphatase-interacting protein 1	270488
EPX	Eosinophil peroxidase	262194
LMAN2	Vesicular integral-membrane protein VIP36	261103
KRT2	Keratin, type II cytoskeletal 2 epidermal	254139
RAP1A	Ras-related protein Rap-1A	250341
KRT9	Keratin, type I cytoskeletal 9	249725
RAB11B	Ras-related protein Rab-11B	238668
HDGF	Hepatoma-derived growth factor	231894
NPC2	NPC intracellular cholesterol transporter 2	216299
MNDA	Myeloid cell nuclear differentiation antigen	214682
MGAM	Maltase-glucoamylase	214377
CAMP	Cathelicidin antimicrobial peptide	214215

ALB	Albumin	207732
DNM2	Dynamin-2	207727
VPS26A	Vacuolar protein sorting-associated protein 26A	207034
RNASET2	Ribonuclease T2	206820
KRT14	Keratin, type I cytoskeletal 14	204474
B2M	Beta-2-microglobulin	202854
SERPINB1	Leukocyte elastase inhibitor	197786
S100A11	Protein S100-A11	193948
ANP32B	Acidic leucine-rich nuclear phosphoprotein 32 family member B	191683
GSN	Gelsolin	188038
DDB1	DNA damage-binding protein 1	186316
ANXA4	Annexin A4	181600
PEBP1	Phosphatidylethanolamine-binding protein 1	179674
PLEK	Pleckstrin	172040
GDI1	Rab GDP dissociation inhibitor alpha	170117
PYCARD	Apoptosis-associated speck-like protein containing a CARD	166836
C3	Complement C3	163761
HPX	Hemopexin	159664
ARHGDIA	Rho GDP-dissociation inhibitor 1	158058
RAB1A	Ras-related protein Rab-1A	157836
KERA	Keratocan	157478
SRSF3	Serine/arginine-rich splicing factor	151942

	3	
IGHG1	Immunoglobulin heavy constant gamma 1	151871
GLO1	Lactoylglutathione lyase	151803
RPIA	Ribose-5-phosphate isomerase	151611
CYBA	Cytochrome b-245 light chain	151401
CD177	CD177 antigen	151104
S100P	Protein S100-P	149934
ORM1	Alpha-1-acid glycoprotein 1	145014
PRG2	Bone marrow proteoglycan	143124
CANX	Calnexin	142325
HPGD	15-hydroxyprostaglandin dehydrogenase [NAD(+)]	137987
FN1	Fibronectin	136572
GGCT	Gamma-glutamylcyclotransferase	130483
NEDD8	NEDD8	126058
WAS	Wiskott-Aldrich syndrome protein	124505
DPP3	Dipeptidyl peptidase 3	123997
STOM	Stomatin	121322
SAFB2	Scaffold attachment factor B2	120281
FCGR3B	Low affinity immunoglobulin gamma Fc region receptor III-B	120084
TLN1	Talin-1	119740
PGD	6-phosphogluconate dehydrogenase, decarboxylating	118562
BST1	ADP-ribosyl cyclase/cyclic ADP-ribose hydrolase 2	117182
EIF4B	Eukaryotic translation initiation	116048

	factor 4B	
QDPR	Dihydropteridine reductase	112656
DEFA1; DEFA3	Neutrophil defensin 1	112482
CTBS	Di-N-acetylchitobiase	111707
LAMP2	Lysosome-associated membrane glycoprotein 2	111497
CYCS	Cytochrome c	106158
RAB39A; RAB6A; RAB6B	Ras-related protein Rab-6A	105945
HNRNPA2B1	Heterogeneous nuclear ribonucleoproteins A2/B1	104483
SLC35A5	Probable UDP-sugar transporter protein SLC35A5	103992
APEH	Acylamino-acid-releasing enzyme	102186
HNRNPUL2	Heterogeneous nuclear ribonucleoprotein U-like protein 2	100902
SERPINA1	Alpha-1-antitrypsin	100605
HBA1	Hemoglobin subunit alpha	100594
SRSF2	Serine/arginine-rich splicing factor 2	100293
YWHAE	14-3-3 protein epsilon	99510.1
CALR	Calreticulin	98465.9
EFHD2	EF-hand domain-containing protein D2	97500.4
KRT5	Keratin, type II cytoskeletal 5	96751.6
CHI3L1	Chitinase-3-like protein 1	96525.1

ALDOC	Fructose-bisphosphate aldolase C	94178.8
H2BC12; H2BC12L; H2BC13; H2BC14; H2BC15; H2BC18; H2BC4; H2BC5; H2BC9	Histone H2B type 1-K	94156.6
H4C1	Histone H4	92887.7
LDHA	L-lactate dehydrogenase A chain	91715.9
CLC	Galectin-10	88873.2
HMGB2	High mobility group protein B2	87767.3
PTTG1IP	Pituitary tumor-transforming gene 1 protein-interacting protein	87245.6
HNRNPA1	Heterogeneous nuclear ribonucleoprotein A1	86893.7
MIF	Macrophage migration inhibitory factor	86480.6
SUB1	Activated RNA polymerase II transcriptional coactivator p15	85999.1
HSP90AB1	Heat shock protein HSP 90-beta	85913.8
COL1A1	Collagen alpha-1(I) chain	85607.8
PFN1	Profilin-1	85483.9
YWHAZ	14-3-3 protein zeta/delta	85340.7
APOA1	Apolipoprotein A-I	85152.5
CSTB	Cystatin-B	83403.7

GALNS	N-acetylgalactosamine-6-sulfatase	83004.2
FKBP1A	Peptidyl-prolyl cis-trans isomerase FKBP1A	82184.1
EIF3A	Eukaryotic translation initiation factor 3 subunit A	81932.5
HYDIN	Hydrocephalus-inducing protein homolog	81165.5
GANAB	Neutral alpha-glucosidase AB	80821.7
ENO1	Alpha-enolase	80567.9
CREG1	Protein CREG1	80153.6
CUTA	Protein CutA	79922.3
ARHGEF1	Rho guanine nucleotide exchange factor 1	79457.2
VIM	Vimentin	79241.9
CSTF3	Cleavage stimulation factor subunit 3	79033.9
ARHGEF19	Rho guanine nucleotide exchange factor 19	75430.8
TXNRD1	Thioredoxin reductase 1, cytoplasmic	74773.9
TOLLIP	Toll-interacting protein	73665.4
ARHGAP25	Rho GTPase-activating protein 25	73546.8
H2AC11; H2AC12; H2AC14; H2AC18; H2AC20; H2AC4;	Histone H2A type 1-B/E	73262.5

H2AC6; H2AC7; H2AJ; H2AW		
NSF	Vesicle-fusing ATPase	73168.2
ACYP1	Acylphosphatase-1	73149.4
ADK	Adenosine kinase	72602.9
SIRPA	Tyrosine-protein phosphatase non-receptor type substrate 1	72445.9
SPRR2E	Small proline-rich protein 2E	71496.1
KCTD12	BTB/POZ domain-containing protein KCTD12	70706.9
SNRPB; SNRPN	Small nuclear ribonucleoprotein-associated proteins B and B'	70508.8
ITIH2	Inter-alpha-trypsin inhibitor heavy chain H2	69614.8
PTMA	Prothymosin alpha	69055.3
PZP	Pregnancy zone protein	68853.6
CRYZ	Quinone oxidoreductase	67952.9
LASP1	LIM and SH3 domain protein 1	66838.8
LSM8	U6 snRNA-associated Sm-like protein LSm8	65656.4
STMN1	Stathmin	65375.1
FBP1	Fructose-1,6-bisphosphatase 1	65029.1
TP53I3	Quinone oxidoreductase PIG3	65006.5
H3-3A; H3-4; H3C1; H3C15	Histone H3.1	63758.9
CYBB	Cytochrome b-245 heavy chain	63675.5
CORO1A	Coronin-1A	63640.7

GLOD4	Glyoxalase domain-containing protein 4	63357.7
TRIR	Telomerase RNA component interacting RNase	63333.1
MLEC	Malectin	63211.7
P4HB	Protein disulfide-isomerase	62432.3
MSRA	Mitochondrial peptide methionine sulfoxide reductase	61336.9
OGFR	Opioid growth factor receptor	60831.5
NME1	Nucleoside diphosphate kinase A	60697.7
HCLS1	Hematopoietic lineage cell-specific protein	60630.6
CTSZ	Cathepsin Z	60233.6
HEPH	Hephaestin	58518.2
CEACAM8	Carcinoembryonic antigen-related cell adhesion molecule 8	56543.9
ITGAM	Integrin alpha-M	56303.3
PGLYRP1	Peptidoglycan recognition protein 1	56252.3
MYDGF	Myeloid-derived growth factor	56095.2
FMNL1	Formin-like protein 1	55648.9
PLPBP	Pyridoxal phosphate homeostasis protein	55404.5
TGM3	Protein-glutamine gamma-glutamyltransferase E	54841.7
UBE2I	SUMO-conjugating enzyme UBC9	54216
GNS	N-acetylglucosamine-6-sulfatase	54012.9

NCL	Nucleolin	53497.5
CD47	Leukocyte surface antigen CD47	53433.7
RAP1B	Ras-related protein Rap-1b-like protein	53148.6
PTPRK	Receptor-type tyrosine-protein phosphatase kappa	53133.4
TSPO	Translocator protein	53001.7
RAB18	Ras-related protein Rab-18	52831.5
LMNB2	Lamin-B2	52789.9
CTSD	Cathepsin D	52725.2
HNRNPF	Heterogeneous nuclear ribonucleoprotein F	52251.4
KHSRP	Far upstream element-binding protein 2	50920.9
AGL	Glycogen debranching enzyme	50646.9
TFIP11	Tuftelin-interacting protein 11	49849.3
MYO15A	Unconventional myosin-XV	49705.3
MMP8	Neutrophil collagenase	49256.5
TXN	Thioredoxin	49162.1
CPPED1	Serine/threonine-protein phosphatase CPPED1	49044.1
VTN	Vitronectin	48985.3
RAD23B	UV excision repair protein RAD23 homolog B	48852
GSR	Glutathione reductase, mitochondrial	48749.3
FCAR	Immunoglobulin alpha Fc receptor	48200.6
USP17L1;	Ubiquitin carboxyl-terminal	47864.1

USP17L10; USP17L11; USP17L12; USP17L13; USP17L15; USP17L17; USP17L18; USP17L19; USP17L20; USP17L21; USP17L22; USP17L24; USP17L3; USP17L5	hydrolase 17-like protein 3	
EIF6	Eukaryotic translation initiation factor 6	47771.9
FLNB	Filamin-B	47629.2
DNAJA2	DnaJ homolog subfamily A member 2	47459.5
SSBP1	Single-stranded DNA-binding protein, mitochondrial	47453.8
PPIL1	Peptidyl-prolyl cis-trans isomerase-like 1	47127.8
LSM3	U6 snRNA-associated Sm-like protein LSm3	47071.9
APOBR	Apolipoprotein B receptor	46814.7
CSTA	Cystatin-A	46681.7
GCHFR	GTP cyclohydrolase 1 feedback	46585.2

	regulatory protein	
DIAPH1	Protein diaphanous homolog 1	46576.4
HMGN1	Non-histone chromosomal protein HMG-14	46552.9
LDHB	L-lactate dehydrogenase B chain	46282.9
SLC2A3	Solute carrier family 2, facilitated glucose transporter member 3	45956.3
PGK1	Phosphoglycerate kinase 1	44825.4
STX7	Syntaxin-7	44803.7
PRDX1	Peroxiredoxin-1	44063.3
HMGB1	High mobility group protein B1	44028.8
HNRNPK	Heterogeneous nuclear ribonucleoprotein K	43955.3
GGH	Gamma-glutamyl hydrolase	43915.1
SPTAN1	Spectrin alpha chain, non-erythrocytic 1	43799.1
PSAP	Prosaposin	43590.1
GRB2	Growth factor receptor-bound protein 2	43531.5
VASP	Vasodilator-stimulated phosphoprotein	43478.3
HSP90B1	Endoplasmin	43409.3
XP32	Skin-specific protein 32	43286.5
RAB11FIP1	Rab11 family-interacting protein 1	42690.7
LSM7	U6 snRNA-associated Sm-like protein LSm7	42548.5
CALML5	Calmodulin-like protein 5	42383.4
PPP6R1	Serine/threonine-protein	42256.2

	phosphatase 6 regulatory subunit 1	
GLIPR2	Golgi-associated plant pathogenesis-related protein 1	42042.9
ACYP2	Acylphosphatase-2	41062.6
CRISP3	Cysteine-rich secretory protein 3	40643
HSPA4	Heat shock 70 kDa protein 4	40362.5
GAPDH	Glyceraldehyde-3-phosphate dehydrogenase	40105.1
ALAD	Delta-aminolevulinic acid dehydratase	40084.2
PTPRJ	Receptor-type tyrosine-protein phosphatase eta	39622.5
CPNE2	Copine-2	39520.8
FBP2	Fructose-1,6-bisphosphatase isozyme 2	39293.4
YWHAG	14-3-3 protein gamma	38820.6
CAPG	Macrophage-capping protein	38781.2
QPCT	Glutaminyl-peptide cyclotransferase	38600.4
SNRPD3	Small nuclear ribonucleoprotein Sm D3	38577.7
IL1RN	Interleukin-1 receptor antagonist protein	38549.5
UFD1	Ubiquitin recognition factor in ER-associated degradation protein 1	38092.5
G6PD	Glucose-6-phosphate 1-dehydrogenase	38017.7
SPTBN1	Spectrin beta chain, non-	37419.8

	erythrocytic 1	
PKM	Pyruvate kinase PKM	36680.6
LCP2	Lymphocyte cytosolic protein 2	36644
HSPE1	10 kDa heat shock protein, mitochondrial	36037.6
HMGN2	Non-histone chromosomal protein HMG-17	35843.5
SLC25A24	Calcium-binding mitochondrial carrier protein SCaMC-1	35262.1
EEF1D	Elongation factor 1-delta	35154.5
GBE1	1,4-alpha-glucan-branching enzyme	34918.9
FCGR3A	Low affinity immunoglobulin gamma Fc region receptor III-A	34710.1
ACTN4	Alpha-actinin-4	34463.2
RAC2	Ras-related C3 botulinum toxin substrate 2	34361.7
CCP110	Centriolar coiled-coil protein of 110 kDa	34300.9
CAPZA1	F-actin-capping protein subunit alpha-1	34094.7
ZW10	Centromere/kinetochore protein zw10 homolog	33742.6
PRDX6	Peroxiredoxin-6	33735.9
AIF1	Allograft inflammatory factor 1	33422.2
PRKCB	Protein kinase C beta type	33311.1
SPRR1A; SPRR1B	Cornifin-B	33308.5

BANF1	Barrier-to-autointegration factor	33194.5
USP1	Ubiquitin carboxyl-terminal hydrolase 1	32787.8
RCSD1	CapZ-interacting protein	32170.5
NAMPT	Nicotinamide phosphoribosyltransferase	31782.1
GNB1; GNB2	Guanine nucleotide-binding protein G(I)/G(S)/G(T) subunit beta-1	31757.7
PDCD10	Programmed cell death protein 10	31518
HNRNPA3	Heterogeneous nuclear ribonucleoprotein A3	31199.9
SNRPG; SNRPGP15	Putative small nuclear ribonucleoprotein G-like protein 15	30131.3
ALDH1A2; ALDH2	Retinal dehydrogenase 2	30046.6
ALYREF	THO complex subunit 4	29884.7
LTA4H	Leukotriene A-4 hydrolase	29449.9
HNRNPH1	Heterogeneous nuclear ribonucleoprotein H	29337.2
XPNPEP3	Xaa-Pro aminopeptidase 3	29217.5
SFPQ	Splicing factor, proline- and glutamine-rich	29053.2
APMAP	Adipocyte plasma membrane-associated protein	28952.9
PIK3R1	Phosphatidylinositol 3-kinase regulatory subunit alpha	28906.3

DLST	Dihydrolipoyllysine-residue succinyltransferase component of 2-oxoglutarate dehydrogenase complex, mitochondrial	28881.2
MARCKSL1	MARCKS-related protein	28831.6
GPD2	Glycerol-3-phosphate dehydrogenase, mitochondrial	28826
PPP6C	Serine/threonine-protein phosphatase 6 catalytic subunit	28722.5
HEBP2	Heme-binding protein 2	28688.3
C4A; C4B	Complement C4-A	28469.9
ATP10B	Phospholipid-transporting ATPase VB	28447.6
CLINT1	Clathrin interactor 1	28363.8
GSTP1	Glutathione S-transferase P	28263.5
GGACT	Gamma-glutamylaminecyclotransferase	28158
SRRT	Serrate RNA effector molecule homolog	28126.5
ALOX5AP	Arachidonate 5-lipoxygenase-activating protein	28061
AIMP1	Aminoacyl tRNA synthase complex-interacting multifunctional protein 1	27730
VAV1	Proto-oncogene vav	27713.9
SORL1	Sortilin-related receptor	27437.8
SNRPD1	Small nuclear ribonucleoprotein Sm D1	27406.8

SNRNP200	U5 small nuclear ribonucleoprotein 200 kDa helicase	27311.6
CUTC	Copper homeostasis protein cutC homolog	27041.4
PGAM1	Phosphoglycerate mutase 1	27036
SPATA24	Spermatogenesis-associated protein 24	26980
SERPIND1	Heparin cofactor 2	26969.3
PPP4C	Serine/threonine-protein phosphatase 4 catalytic subunit	26865.6
STMN2	Stathmin-2	26782
ANP32E	Acidic leucine-rich nuclear phosphoprotein 32 family member E	26641.3
PYGL	Glycogen phosphorylase, liver form	26197.2
CD53	Leukocyte surface antigen CD53	26134.8
HNRNPM	Heterogeneous nuclear ribonucleoprotein M	26079.6
GNAI2	Guanine nucleotide-binding protein G(i) subunit alpha-2	26074.6
MICAL3	[F-actin]-monooxygenase MICAL3	25990.6
DNAJA1	DnaJ homolog subfamily A member 1	25851.1
NHLRC3	NHL repeat-containing protein 3	25617.8
FABP5	Fatty acid-binding protein 5	25506.4

TPM3	Tropomyosin alpha-3 chain	25458.6
HNRNPC	Heterogeneous nuclear ribonucleoproteins C1/C2	25290
HSP90AA1	Heat shock protein HSP 90-alpha	25154.2
NPEPPS	Puromycin-sensitive aminopeptidase	25106.8
ANXA2	Annexin A2	24994.6
CFI	Complement factor I	24896
STIP1	Stress-induced-phosphoprotein 1	24884.4
THYN1	Thymocyte nuclear protein 1	24638.9
LSM4	U6 snRNA-associated Sm-like protein LSm4	24634.7
FERMT3	Fermitin family homolog 3	24632.6
SET; SETSIP	Protein SETSIP	24175.6
H1-2; H1-3; H1-4	Histone H1.4	24163.5
TAGLN2	Transgelin-2	24129.8
PTPN6	Tyrosine-protein phosphatase non-receptor type 6	24114.5
NSFL1C	NSFL1 cofactor p47	24030.4
TREM1	Triggering receptor expressed on myeloid cells 1	23937.2
KRT77	Keratin, type II cytoskeletal 1b	23754.7
LSM6	U6 snRNA-associated Sm-like protein LSm6	23716.6
SDCBP	Syntenin-1	23676.8
SRSF4	Serine/arginine-rich splicing factor 4	23626.8

PHGDH	D-3-phosphoglycerate dehydrogenase	23566
VPS4A	Vacuolar protein sorting-associated protein 4A	23209.9
DSP	Desmoplakin	23106.4
SH3KBP1	SH3 domain-containing kinase-binding protein 1	23080.2
IGHV3-15; IGHV3-72; IGHV3-73	Immunoglobulin heavy variable 3-15	23057.9
SUMO2; SUMO3; SUMO4	Small ubiquitin-related modifier 3	22988
CASP14	Caspase-14	22976
LRRFIP1	Leucine-rich repeat flightless-interacting protein 1	22874.8
PRDX3	Thioredoxin-dependent peroxide reductase, mitochondrial	22737.2
KRT6A	Keratin, type II cytoskeletal 6A	22692.8
HCAR2; HCAR3	Hydroxycarboxylic acid receptor 3	22658.8
ARPC4	Actin-related protein 2/3 complex subunit 4	22330.8
FCN1	Ficolin-1	22240.3
PSMA7; PSMA8	Proteasome subunit alpha type-7	22204.7
SNRPD2	Small nuclear ribonucleoprotein Sm D2	22034.5

RAB6C; RAB6D	Ras-related protein Rab-6D	21948.6
GCA	Grancalcin	21837.3
AGA	N(4)-(beta-N-acetylglucosaminyl)-L-asparaginase	21660.8
CR1	Complement receptor type 1	21643.3
CD63	CD63 antigen	21590.8
SF3A1	Splicing factor 3A subunit 1	21545.8
AP1B1	AP-1 complex subunit beta-1	21461.3
PLCG2	1-phosphatidylinositol 4,5-bisphosphate phosphodiesterase gamma-2	21383.4
USP14	Ubiquitin carboxyl-terminal hydrolase 14	21258.9
FGD6	FYVE, RhoGEF and PH domain-containing protein 6	21224
SERPINB8	Serpin B8	21165.8
RPS7	40S ribosomal protein S7	21003.8
BAG6	Large proline-rich protein BAG6	20966.1
PDS5B	Sister chromatid cohesion protein PDS5 homolog B	20871.2
FNBP1	Formin-binding protein 1	20808.2
FLII	Protein flightless-1 homolog	20621.2
HP1BP3	Heterochromatin protein 1-binding protein 3	20563.2
CTSH	Pro-cathepsin H	20535.9
ATP5F1D	ATP synthase subunit delta,	20495.2

	mitochondrial	
PRAM1	PML-RARA-regulated adapter molecule 1	20458
TUBA1B	Tubulin alpha-1B chain	20456.2
PPP3CA	Serine/threonine-protein phosphatase 2B catalytic subunit alpha isoform	20437.1
PLEC	Plectin	20279.6
EHD1	EH domain-containing protein 1	20186.9
HSPA9	Stress-70 protein, mitochondrial	20110.1
COL1A2	Collagen alpha-2(I) chain	20067.3
TCEAL3	Transcription elongation factor A protein-like 3	19920
LITAF	Lipopolysaccharide-induced tumor necrosis factor-alpha factor	19898.5
NUTF2	Nuclear transport factor 2	19860.3
LYN	Tyrosine-protein kinase Lyn	19818.8
HVCN1	Voltage-gated hydrogen channel 1	19784.8
CAST	Calpastatin	19625.7
ARAP1	Arf-GAP with Rho-GAP domain, ANK repeat and PH domain-containing protein 1	19505.7
IGKC	Immunoglobulin kappa constant	19391.6
NQO2	Ribosyldihydronicotinamide dehydrogenase [quinone]	19270.9
C11orf54	Ester hydrolase C11orf54	18936
RCC2	Protein RCC2	18777.8
IGHG2	Immunoglobulin heavy constant	18660.3

	gamma 2	
PTX3	Pentraxin-related protein PTX3	18618.4
FASN	Fatty acid synthase	18582.1
WARS1	Tryptophan--tRNA ligase, cytoplasmic	18526
HBB	Hemoglobin subunit beta	18507.3
LSM2	U6 snRNA-associated Sm-like protein LSm2	18416
CPNE3	Copine-3	18345.1
LILRB2	Leukocyte immunoglobulin-like receptor subfamily B member 2	18207.1
PIP4P2	Type 2 phosphatidylinositol 4,5-bisphosphate 4-phosphatase	18173.4
MAP1LC3B; MAP1LC3B2	Microtubule-associated proteins 1A/1B light chain 3 beta 2	18121.4
GRN	Progranulin	18090.3
CEACAM6	Carcinoembryonic antigen-related cell adhesion molecule 6	18086.3
DDT; DDTL	D-dopachrome decarboxylase-like protein	18082.6
RALB	Ras-related protein Ral-B	18069.6
CBX3	Chromobox protein homolog 3	17920.6
RETN	Resistin	17882.8
CELF2	CUGBP Elav-like family member 2	17779
ILF3	Interleukin enhancer-binding factor 3	17756.4
CLIC1	Chloride intracellular channel	17732

	protein 1	
PLG	Plasminogen	17708.4
TXNDC17	Thioredoxin domain-containing protein 17	17614.9
RAB1B	Ras-related protein Rab-1B	17524.2
PDE6D	Retinal rod rhodopsin-sensitive cGMP 3',5'-cyclic phosphodiesterase subunit delta	17353.2
VNN2	Pantetheine hydrolase VNN2	17255.4
RAP2B	Ras-related protein Rap-2b	17217.8
SMARCA2	Probable global transcription activator SNF2L2	17166.5
ITIH4	Inter-alpha-trypsin inhibitor heavy chain H4	16967.6
EPDR1	Mammalian ependymin-related protein 1	16883.1
SYNGR2	Synaptogyrin-2	16854.6
ARL8B	ADP-ribosylation factor-like protein 8B	16643.5
DYSF	Dysferlin	16642.8
ANKRD36; ANKRD36B; ANKRD36C	Ankyrin repeat domain-containing protein 36A	16600.8
LBR	Delta(14)-sterol reductase LBR	16509.5
SLC16A3	Monocarboxylate transporter 4	16395.9
ENPP4	Bis(5'-adenosyl)-triphosphatase ENPP4	16248.7
COL11A2	Collagen alpha-2(XI) chain	16246.3

RNF167	E3 ubiquitin-protein ligase RNF167	16108.6
MYL12A; MYL12B	Myosin regulatory light chain 12B	16026.1
MYO9B	Unconventional myosin-IXb	15973.5
DMRTA2	Doublesex- and mab-3-related transcription factor A2	15885.6
PPIL3	Peptidyl-prolyl cis-trans isomerase-like 3	15762.4
CCDC88C	Protein Daple	15710.6
GPX4	Phospholipid hydroperoxide glutathione peroxidase	15699
MYL6	Myosin light polypeptide 6	15666.6
ICAM3	Intercellular adhesion molecule 3	15606
TKT	Transketolase	15507
TPI1	Triosephosphate isomerase	15503.1
MCEMP1	Mast cell-expressed membrane protein 1	15464.9
AHNAK	Neuroblast differentiation-associated protein AHNAK	15423
LZIC	Protein LZIC	15422.7
QSOX1	Sulfhydryl oxidase 1	15408.4
SRRM1	Serine/arginine repetitive matrix protein 1	15365.5
RHOG	Rho-related GTP-binding protein RhoG	15322.5
EEF1A2	Elongation factor 1-alpha 2	15278.6
PON3	Serum paraoxonase/lactonase 3	15212.1

CAPZB	F-actin-capping protein subunit beta	15182.1
NIPSNAP3A	Protein NipSnap homolog 3A	15170.4
SGTB	Small glutamine-rich tetratricopeptide repeat-containing protein beta	15158.4
BIN2	Bridging integrator 2	15145.1
EEF1G	Elongation factor 1-gamma	15085.7
ACTA2; ACTG2	Actin, aortic smooth muscle	15023.6
ABCA12	Glucosylceramide transporter ABCA12	14997.1
HRNR	Hornerin	14987.7
LGALS3BP	Galectin-3-binding protein	14978.2
SRSF6	Serine/arginine-rich splicing factor 6	14955.3
CERT1	Ceramide transfer protein	14942.9
TUBB	Tubulin beta chain	14941
CAPN1	Calpain-1 catalytic subunit	14940.4
NME2; NME2P1	Putative nucleoside diphosphate kinase	14938.7
MYADM	Myeloid-associated differentiation marker	14910.8
PDS5A	Sister chromatid cohesion protein PDS5 homolog A	14818
PDLIM5	PDZ and LIM domain protein 5	14737.4
PRDX4	Peroxiredoxin-4	14615.4
MANF	Mesencephalic astrocyte-derived	14615.2

	neurotrophic factor	
DDX1	ATP-dependent RNA helicase DDX1	14480.1
RAB8A; RAB8B	Ras-related protein Rab-8A	14334.8
SMIM24	Small integral membrane protein 24	14310.6
LAMP1	Lysosome-associated membrane glycoprotein 1	14272.2
HMGB3	High mobility group protein B3	14244.4
CP	Ceruloplasmin	14212.9
NINL	Ninein-like protein	14131.6
MYG1	MYG1 exonuclease	14070.1
TPK1	Thiamin pyrophosphokinase 1	14063.4
HDGFL2	Hepatoma-derived growth factor-related protein 2	14002.2
SASH3	SAM and SH3 domain-containing protein 3	14001.9
SUPT16H	FACT complex subunit SPT16	13983.9
ARPC1B	Actin-related protein 2/3 complex subunit 1B	13904.6
PSMA1	Proteasome subunit alpha type-1	13785.9
PARVG	Gamma-parvin	13740.7
EEF2	Elongation factor 2	13724.5
CRKL	Crk-like protein	13672.5
PDIA4	Protein disulfide-isomerase A4	13670.2
HYOU1	Hypoxia up-regulated protein 1	13567.7
RAB5A;	Ras-related protein Rab-5A	13530.2

RAB5B; RAB5C		
A1BG	Alpha-1B-glycoprotein	13344.8
TXNDC5	Thioredoxin domain-containing protein 5	13342.8
STX3	Syntaxin-3	13328.8
RPN1	Dolichyl-diphosphooligosaccharide--protein glycosyltransferase subunit 1	13298.6
RPS2	40S ribosomal protein S2	13264.3
PGM1	Phosphoglucomutase-1	13215.3
LAIR1; LAIR2	Leukocyte-associated immunoglobulin-like receptor 1	13190.4
TSN	Translin	13176.5
LRG1	Leucine-rich alpha-2-glycoprotein	13096.9
SNRPA; SNRPB2	U2 small nuclear ribonucleoprotein B''	13090.4
ADSS2	Adenylosuccinate synthetase isozyme 2	13028.4
PLAA	Phospholipase A-2-activating protein	12960.3
UGP2	UTP--glucose-1-phosphate uridylyltransferase	12924.5
HLA-B	HLA class I histocompatibility antigen, B alpha chain	12912.6
LGALS7	Galectin-7	12831.4
H6PD	GDH/6PGL endoplasmic bifunctional protein	12798.8

UBE2L3	Ubiquitin-conjugating enzyme E2 L3	12736.6
PDIA3	Protein disulfide-isomerase A3	12616.9
EIF4A1	Eukaryotic initiation factor 4A-I	12600.8
TGOLN2	Trans-Golgi network integral membrane protein 2	12544
PDCD6IP	Programmed cell death 6-interacting protein	12517.8
NUDT3	Diphosphoinositol polyphosphate phosphohydrolase 1	12465.5
ANXA11	Annexin A11	12453.3
SVIL	Supervillin	12445.6
HSPD1	60 kDa heat shock protein, mitochondrial	12433.4
ACLY	ATP-citrate synthase	12426.5
SUMF2	Inactive C-alpha-formylglycine-generating enzyme 2	12412.7
CFB	Complement factor B	12387.1
IGF2R	Cation-independent mannose-6-phosphate receptor	12355.1
SUPT5H	Transcription elongation factor SPT5	12335.8
PECAM1	Platelet endothelial cell adhesion molecule	12299
CEACAM1	Carcinoembryonic antigen-related cell adhesion molecule 1	12270.2
SELL	L-selectin	12196.4
PPP3R1	Calcineurin subunit B type 1	12188.3

DNAJC8	DnaJ homolog subfamily C member 8	12166
IQGAP2	Ras GTPase-activating-like protein IQGAP2	12134.2
PDLIM2	PDZ and LIM domain protein 2	12016.7
DCUN1D1	DCN1-like protein 1	11998.8
VDAC1	Voltage-dependent anion-selective channel protein 1	11993.1
MAP3K3	Mitogen-activated protein kinase kinase kinase 3	11966.4
DEF6	Differentially expressed in FDCP 6 homolog	11959.4
SYNCRIP	Heterogeneous nuclear ribonucleoprotein Q	11925.5
ADGRE3	Adhesion G protein-coupled receptor E3	11899.9
PPIF	Peptidyl-prolyl cis-trans isomerase F, mitochondrial	11886.9
TMOD3	Tropomodulin-3	11878
KRT18	Keratin, type I cytoskeletal 18	11854.6
MDH2	Malate dehydrogenase, mitochondrial	11840.7
SLC15A4	Solute carrier family 15 member 4	11666.6
CLEC4D	C-type lectin domain family 4 member D	11642.6
CLIP1; CLIP2	CAP-Gly domain-containing linker protein 1	11627.4
HLA-A	HLA class I histocompatibility	11500

	antigen, A alpha chain	
ARID3A	AT-rich interactive domain-containing protein 3A	11314.3
ECI1	Enoyl-CoA delta isomerase 1, mitochondrial	11301.5
GOT1	Aspartate aminotransferase, cytoplasmic	11291.1
DNAJC5	DnaJ homolog subfamily C member 5	11185.7
FH	Fumarate hydratase, mitochondrial	11170.5
PSAT1	Phosphoserine aminotransferase	11101.6
PEPD	Xaa-Pro dipeptidase	11026.1
SIRPB1	Signal-regulatory protein beta-1	10953.1
NCF1; NCF1B; NCF1C	Putative neutrophil cytosol factor 1B	10896.9
RAB27A	Ras-related protein Rab-27A	10856.2
BLMH	Bleomycin hydrolase	10829.3
PSMA6	Proteasome subunit alpha type-6	10773.9
KIF27	Kinesin-like protein KIF27	10643.2
DDOST	Dolichyl-diphosphooligosaccharide--protein glycosyltransferase 48 kDa subunit	10619.3
SCRN2	Secernin-2	10592.2
EZR	Ezrin	10531.1
EEA1	Early endosome antigen 1	10512.1
BPI	Bactericidal permeability-increasing protein	10511.1
BRD4	Bromodomain-containing protein	10506.3

	4	
NMNAT1	Nicotinamide/nicotinic acid mononucleotide adenylyltransferase 1	10464.9
HARS1	Histidine--tRNA ligase, cytoplasmic	10420
ARG1	Arginase-1	10376.1
ASS1	Argininosuccinate synthase	10370.6
THBS4	Thrombospondin-4	10356.4
ATP5F1B	ATP synthase subunit beta, mitochondrial	10326.5
P2RY13	P2Y purinoceptor 13	10294.7
RDX	Radixin	10290.8
NPM1	Nucleophosmin	10284
NUCKS1	Nuclear ubiquitous casein and cyclin-dependent kinase substrate 1	10274.1
F5	Coagulation factor V	10215.1
STBD1	Starch-binding domain-containing protein 1	10191
GLRX	Glutaredoxin-1	10167.7
PITHD1	PITH domain-containing protein 1	10158.8
HNRNPL	Heterogeneous nuclear ribonucleoprotein L	10154.1
HPRT1	Hypoxanthine-guanine phosphoribosyltransferase	10153.8
RAD23A	UV excision repair protein RAD23 homolog A	10148.8

CXCL8	Interleukin-8	10067.5
EIF3J	Eukaryotic translation initiation factor 3 subunit J	10058.2
HNRNPR	Heterogeneous nuclear ribonucleoprotein R	10019.4
CLEC12A	C-type lectin domain family 12 member A	9958.21
ARHGEF18	Rho guanine nucleotide exchange factor 18	9936.38
CD33	Myeloid cell surface antigen CD33	9932.83
NCSTN	Nicastrin	9890.77
SERPINB10	Serpin B10	9888.16
CNK3/IPCEF1;IPCEF1	CNK3/IPCEF1 fusion protein	9835.73
NCK2	Cytoplasmic protein NCK2	9802.27
H2AZ1; H2AZ2	Histone H2A.Z	9790.38
FBXO6	F-box only protein 6	9754.81
DNAJB1	DnaJ homolog subfamily B member 1	9740.96
FKBP2	Peptidyl-prolyl cis-trans isomerase FKBP2	9737.52
RHOA; RHOC	Rho-related GTP-binding protein RhoC	9728.92
IGKV2-24; IGKV2-29; IGKV2D-24; IGKV2D-26;	Probable non-functional immunoglobulin kappa variable 2D-24	9722

IGKV2D-29		
F2	Prothrombin	9714.56
PLTP	Phospholipid transfer protein	9702.8
MYCBP2	E3 ubiquitin-protein ligase MYCBP2	9691.05
COPB1	Coatomer subunit beta	9674.11
LGALS1	Galectin-1	9660.19
ENOPH1	Enolase-phosphatase E1	9658.62
FAHD1	Acylpyruvase FAHD1, mitochondrial	9658.05
LST1	Leukocyte-specific transcript 1 protein	9639.09
EPRS1	Bifunctional glutamate/proline--tRNA ligase	9584.91
SMARCC2	SWI/SNF complex subunit SMARCC2	9498.92
RBBP5	Retinoblastoma-binding protein 5	9436.35
CPS1	Carbamoyl-phosphate synthase [ammonia], mitochondrial	9436
HNRNPU	Heterogeneous nuclear ribonucleoprotein U	9371.1
TNC	Tenascin	9368.9
ARSB	Arylsulfatase B	9320.5
AZGP1	Zinc-alpha-2-glycoprotein	9214.93
FKBP4	Peptidyl-prolyl cis-trans isomerase FKBP4	9149.79
ACTR2	Actin-related protein 2	9055.31
ERH	Enhancer of rudimentary homolog	9048.84

CCT8	T-complex protein 1 subunit theta	9045.66
CAMK2G	Calcium/calmodulin-dependent protein kinase type II subunit gamma	9000.74
USP8	Ubiquitin carboxyl-terminal hydrolase 8	8966.12
SF3B2	Splicing factor 3B subunit 2	8955.7
ASAH1	Acid ceramidase	8937.22
ESYT1	Extended synaptotagmin-1	8934.43
SLC25A5	ADP/ATP translocase 2	8886.44
SERPINF1	Pigment epithelium-derived factor	8872.23
HNRNPUL1	Heterogeneous nuclear ribonucleoprotein U-like protein 1	8843.69
VPS26B	Vacuolar protein sorting-associated protein 26B	8827.69
EEF1A1; EEF1A1P5	Elongation factor 1-alpha 1	8811.5
IQGAP3	Ras GTPase-activating-like protein IQGAP3	8683.56
IFI16	Gamma-interferon-inducible protein 16	8682.79
HDGFL3	Hepatoma-derived growth factor-related protein 3	8672.46
LHPP	Phospholysine phosphohistidine inorganic pyrophosphate phosphatase	8671.6
IGLC2; IGLC3	Immunoglobulin lambda constant 2	8627.38

DCD	Dermcidin	8548.7
SNRPE	Small nuclear ribonucleoprotein E	8527.23
PPP2CA	Serine/threonine-protein phosphatase 2A catalytic subunit alpha isoform	8420.48
TMEM30A	Cell cycle control protein 50A	8369.07
PSMC3	26S proteasome regulatory subunit 6A	8326.63
RPL37A	60S ribosomal protein L37a	8318.99
CYRIB	CYFIP-related Rac1 interactor B	8304.73
REL	Proto-oncogene c-Rel	8300.96
HNRNPD	Heterogeneous nuclear ribonucleoprotein D0	8294.69
PLXNC1	Plexin-C1	8274.81
PPP1R12A	Protein phosphatase 1 regulatory subunit 12A	8221.15
PTPRC	Receptor-type tyrosine-protein phosphatase C	8210.27
SLC25A3	Phosphate carrier protein, mitochondrial	8151.96
SLC17A5	Sialin	8118.2
RNPEP	Aminopeptidase B	8101.09
PDCD6	Programmed cell death protein 6	8065.69
YBX1	Y-box-binding protein 1	8058.16
APOOL	MICOS complex subunit MIC27	8040.51
ERP29	Endoplasmic reticulum resident protein 29	8024.73
SOD1	Superoxide dismutase [Cu-Zn]	7991.61

PRKDC	DNA-dependent protein kinase catalytic subunit	7988.91
PCNA	Proliferating cell nuclear antigen	7826.07
MCU	Calcium uniporter protein, mitochondrial	7764.53
SIRPB1	Signal-regulatory protein beta-1 isoform 3	7739.33
RAVER1	Ribonucleoprotein PTB-binding 1	7713.77
LTB4R	Leukotriene B4 receptor 1	7700.87
CSK	Tyrosine-protein kinase CSK	7683.58
CYP4F3	Cytochrome P450 4F3	7621.2
NCF4	Neutrophil cytosol factor 4	7598.87
DTD2	D-aminoacyl-tRNA deacylase 2	7590.64
CASP3	Caspase-3	7562.32
KPRP	Keratinocyte proline-rich protein	7550.86
ARF1; ARF3; ARF5	ADP-ribosylation factor 3	7478.07
HADH	Hydroxyacyl-coenzyme A dehydrogenase, mitochondrial	7426.31
TMPO	Lamina-associated polypeptide 2, isoform alpha	7353.97
HYI	Putative hydroxypyruvate isomerase	7348.55
COL3A1	Collagen alpha-1(III) chain	7319.32
NIT2	Omega-amidase NIT2	7315.09
CFL1	Cofilin-1	7217.42
ACIN1	Apoptotic chromatin condensation inducer in the nucleus	7211.51

PNP	Purine nucleoside phosphorylase	7181.69
DSC1	Desmocollin-1	7174.27
PDIA6	Protein disulfide-isomerase A6	7128.32
BSG	Basigin	7126.89
CLEC5A	C-type lectin domain family 5 member A	7008.48
CKB	Creatine kinase B-type	7005.95
CAPZA2	F-actin-capping protein subunit alpha-2	6957.97
PLEKHO2	Pleckstrin homology domain-containing family O member 2	6942.7
SEPTIN7	Septin-7	6901.42
NIT1	Deaminated glutathione amidase	6890.36
ANXA7	Annexin A7	6880.55
KRT17	Keratin, type I cytoskeletal 17	6835.52
COL9A3	Collagen alpha-3(IX) chain	6791.79
MARCKS	Myristoylated alanine-rich C-kinase substrate	6786.34
EIF2S1	Eukaryotic translation initiation factor 2 subunit 1	6758.65
APOB	Apolipoprotein B-100	6745.86
GNAS	Guanine nucleotide-binding protein G(s) subunit alpha isoforms short	6687.4
PTBP1	Polypyrimidine tract-binding protein 1	6678.23
ACADVL	Very long-chain specific acyl-CoA dehydrogenase, mitochondrial	6643.63

TBCA	Tubulin-specific chaperone A	6589.5
PLAU	Urokinase-type plasminogen activator	6578.02
CCT7	T-complex protein 1 subunit eta	6556.34
ANPEP	Aminopeptidase N	6534.82
JUP	Junction plakoglobin	6468.82
PCBD1	Pterin-4-alpha-carbinolamine dehydratase	6462.36
HAAO	3-hydroxyanthranilate 3,4-dioxygenase	6424.87
ALDH18A1	Delta-1-pyrroline-5-carboxylate synthase	6337.38
GABARAP; GABARAPL2	Gamma-aminobutyric acid receptor-associated protein	6316.54
SPN	Leukosialin	6303.7
XRCC6	X-ray repair cross-complementing protein 6	6262.97
MTHFD1	C-1-tetrahydrofolate synthase, cytoplasmic	6261.49
EXOC2	Exocyst complex component 2	6227.33
PSMA3	Proteasome subunit alpha type-3	6185.54
PSMB6	Proteasome subunit beta type-6	6125.21
SLC38A10	Putative sodium-coupled neutral amino acid transporter 10	6118.35
RAB29; RAB32; RAB38	Ras-related protein Rab-7L1	6118
KRT16	Keratin, type I cytoskeletal 16	6032.68

RPS3	40S ribosomal protein S3	6031.38
DHX15	ATP-dependent RNA helicase DHX15	5991.15
GYS1	Glycogen [starch] synthase, muscle	5979.72
AKR1E2	1,5-anhydro-D-fructose reductase	5977.56
OSBP	Oxysterol-binding protein 1	5952.74
MACROH2A1	Core histone macro-H2A.1	5810.15
HIDE1	Protein HIDE1	5805.96
CRK	Adapter molecule crk	5791.47
JPT1	Jupiter microtubule associated homolog 1	5729.23
GLB1	Beta-galactosidase	5727.39
RRBP1	Ribosome-binding protein 1	5719.67
SRSF5	Serine/arginine-rich splicing factor 5	5601
CDK5RAP2; PDE4DIP	Myomegalin	5597.4
DDX46	Probable ATP-dependent RNA helicase DDX46	5564.47
TOMM40	Mitochondrial import receptor subunit TOM40 homolog	5545.47
TUBA1C	Tubulin alpha-1C chain	5490.01
CASP6	Caspase-6	5401.17
VDAC2	Voltage-dependent anion-selective channel protein 2	5333.78
PHB1	Prohibitin 1	5305.08
ALOX5	Polyunsaturated fatty acid 5-	5220.97

	lipoxygenase	
TOM1	Target of Myb protein 1	5215.99
LMNA	Prelamin-A/C	5199.79
MKNK1; MKNK2	MAP kinase-interacting serine/threonine-protein kinase 1	5150.46
TIAL1	Nucleolysin TIAR	5121.69
GMIP	GEM-interacting protein	5110.04
CD55	Complement decay-accelerating factor	5109.09
SNRPA1	U2 small nuclear ribonucleoprotein A'	5089.19
ERO1A	ERO1-like protein alpha	5089
C5	Complement C5	5057.38
MYLK	Myosin light chain kinase, smooth muscle	5023.13
CYFIP2	Cytoplasmic FMR1-interacting protein 2	4997.02
SCYL3	Protein-associating with the carboxyl-terminal domain of ezrin	4964.69
RAB21	Ras-related protein Rab-21	4894.96
PCK2	Phosphoenolpyruvate carboxykinase [GTP], mitochondrial	4887.95
OXCT1	Succinyl-CoA:3-ketoacid coenzyme A transferase 1, mitochondrial	4818.19
DNAJB6	DnaJ homolog subfamily B member 6	4812.37

IMPDH2	Inosine-5'-monophosphate dehydrogenase 2	4777.82
PABPC1; PABPC1L; PABPC5	Polyadenylate-binding protein 1	4764.82
DENR	Density-regulated protein	4762.84
ASL	Argininosuccinate lyase	4722.33
SHMT2	Serine hydroxymethyltransferase, mitochondrial	4709.74
ACAD9	Complex I assembly factor ACAD9, mitochondrial	4677.88
MAT2A	S-adenosylmethionine synthase isoform type-2	4663.28
TUBB4A; TUBB4B	Tubulin beta-4A chain	4661.34
RNF213	E3 ubiquitin-protein ligase RNF213	4650.52
RNH1	Ribonuclease inhibitor	4639.52
FLG2	Filaggrin-2	4605.75
ARHGAP30	Rho GTPase-activating protein 30	4596.04
NUDT1	Oxidized purine nucleoside triphosphate hydrolase	4531.32
LPCAT2	Lysophosphatidylcholine acyltransferase 2	4527.32
ALPL	Alkaline phosphatase, tissue-nonspecific isozyme	4503.6
LRCH4	Leucine-rich repeat and calponin homology domain-containing	4492.31

	protein 4	
CAD	CAD protein	4453.23
PACSIN2	Protein kinase C and casein kinase substrate in neurons protein 2	4420.06
SEC11A	Signal peptidase complex catalytic subunit SEC11A	4403.66
NAA10	N-alpha-acetyltransferase 10	4395.72
RIN3	Ras and Rab interactor 3	4395.36
RAN	GTP-binding nuclear protein Ran	4382.64
ATP1A1	Sodium/potassium-transporting ATPase subunit alpha-1	4339.13
USP7	Ubiquitin carboxyl-terminal hydrolase 7	4309.17
EML2	Echinoderm microtubule-associated protein-like 2	4309.06
KCNAB2	Voltage-gated potassium channel subunit beta-2	4295.15
RELA	Transcription factor p65	4273.68
FIS1	Mitochondrial fission 1 protein	4273.12
H1-10	Histone H1.10	4257.53
FCER1G	High affinity immunoglobulin epsilon receptor subunit gamma	4226.98
RPL9	60S ribosomal protein L9	4202.49
RBKS	Ribokinase	4176.76
DDX19A; DDX25	ATP-dependent RNA helicase DDX19A	4173.37
AKR1A1	Aldo-keto reductase family 1 member A1	4171.34

PGP	Glycerol-3-phosphate phosphatase	4133.74
MYO18A	Unconventional myosin-XVIIIa	4117.88
CALML3	Calmodulin-like protein 3	4075.48
FTL	Ferritin light chain	4061.4
MOSPD2	Motile sperm domain-containing protein 2	4029.55
ECH1	Delta(3,5)-Delta(2,4)-dienoyl-CoA isomerase, mitochondrial	4007.75
TROAP	Tastin	3945.69
NDUFS8	NADH dehydrogenase [ubiquinone] iron-sulfur protein 8, mitochondrial	3886.71
WDR5	WD repeat-containing protein 5	3852.14
RPS24	40S ribosomal protein S24	3804.33
CLTC; CLTCL1	Clathrin heavy chain 2	3786.22
CC2D1A	Coiled-coil and C2 domain-containing protein 1A	3766.62
HAL	Histidine ammonia-lyase	3719.14
CYSTM1	Cysteine-rich and transmembrane domain-containing protein 1	3700.24
MAN2B2	Epididymis-specific alpha-mannosidase	3686.04
DSG1	Desmoglein-1	3664.33
RPLP0	60S acidic ribosomal protein P0	3658.51
SCCPDH	Saccharopine dehydrogenase-like oxidoreductase	3650.66
RPS15A	40S ribosomal protein S15a	3648.19

CPQ	Carboxypeptidase Q	3644.24
RPL12	60S ribosomal protein L12	3635.74
RPLP1	60S acidic ribosomal protein P1	3552.32
KIAA1586	E3 SUMO-protein ligase KIAA1586	3509.54
FRRS1	Ferric-chelate reductase 1	3493.36
EPS15L1	Epidermal growth factor receptor substrate 15-like 1	3489.15
CUL3	Cullin-3	3413.99
DOCK2	Dedicator of cytokinesis protein 2	3409.04
SF1	Splicing factor 1	3392.41
SLC3A2	4F2 cell-surface antigen heavy chain	3392.18
PSMA5	Proteasome subunit alpha type-5	3379.94
COL2A1; COL5A2	Collagen alpha-1(II) chain	3365.63
HADHA	Trifunctional enzyme subunit alpha, mitochondrial	3298.61
IDH1	Isocitrate dehydrogenase [NADP] cytoplasmic	3289.62
RANBP3	Ran-binding protein 3	3271.64
MIX23	Protein MIX23	3267.27
PAFAH1B2	Platelet-activating factor acetylhydrolase IB subunit alpha2	3256.92
SYNE1	Nesprin-1	3255.59
DDX39A; DDX39B	ATP-dependent RNA helicase DDX39A	3241.89
DNAH3	Dynein axonemal heavy chain 3	3227.5

HNRNPAB	Heterogeneous nuclear ribonucleoprotein A/B	3194.94
CS	Citrate synthase, mitochondrial	3188.71
RPL4	60S ribosomal protein L4	3165.86
RPSA	40S ribosomal protein SA	3148.01
GUSB	Beta-glucuronidase	3145.94
ATP5F1A	ATP synthase subunit alpha, mitochondrial	3099.9
ITM2B	Integral membrane protein 2B	2983.2
G3BP1	Ras GTPase-activating protein-binding protein 1	2877.27
HSD17B10	3-hydroxyacyl-CoA dehydrogenase type-2	2869.76
ENO3	Beta-enolase	2851.53
PSMD5	26S proteasome non-ATPase regulatory subunit 5	2839.5
SPRYD3	SPRY domain-containing protein 3	2835.37
OXSR1	Serine/threonine-protein kinase OSR1	2815.81
RBP4	Retinol-binding protein 4	2796.42
H1-0	Histone H1.0	2773.44
HRAS; KRAS; NRAS	GTPase NRas	2757.9
ARPC2	Actin-related protein 2/3 complex subunit 2	2742.49
SERPINA11	Serpin A11	2742.02
PDCD4	Programmed cell death protein 4	2733.73
SOD2	Superoxide dismutase [Mn],	2729.08

	mitochondrial	
UBA1	Ubiquitin-like modifier-activating enzyme 1	2696.53
HBD	Hemoglobin subunit delta	2690.57
PCBP2	Poly(rC)-binding protein 2	2604.72
SFN	14-3-3 protein sigma	2565.52
VAT1	Synaptic vesicle membrane protein VAT-1 homolog	2551.64
SF3A3	Splicing factor 3A subunit 3	2532.19
RPS3A	40S ribosomal protein S3a	2483.39
ERAP1	Endoplasmic reticulum aminopeptidase 1	2468.24
PREP	Prolyl endopeptidase	2433.19
ITIH1	Inter-alpha-trypsin inhibitor heavy chain H1	2349.45
UBE2D2;UBE2D3	Ubiquitin-conjugating enzyme E2 D3	2280.89
HK2;HK3	Hexokinase-2	2150.95
RPL7	60S ribosomal protein L7	2137.86
APIP	Methylthioribulose-1-phosphate dehydratase	2103.85
IMMT	MICOS complex subunit MIC60	1888.14
ZBTB8OS	Protein archease	1866.52
ALOX12B	Arachidonate 12-lipoxygenase, 12R-type	1801.16
XRCC5	X-ray repair cross-complementing protein 5	1776.09
LONP1	Lon protease homolog,	1759.62

	mitochondrial	
SHROOM2	Protein Shroom2	1752.35
TFRC	Transferrin receptor protein 1	1580.76
IGSF6	Immunoglobulin superfamily member 6	1567.05
RP2	Protein XRP2	1533.16
RPN2	Dolichyl-diphosphooligosaccharide--protein glycosyltransferase subunit 2	1531.32
GSTO1	Glutathione S-transferase omega-1	1473.59
RPL23	60S ribosomal protein L23	1414.12
NFASC	Neurofascin	1381.71
TMEM192	Transmembrane protein 192	1328.66
BRIX1	Ribosome biogenesis protein BRX1 homolog	1283.64

Supplemental Table 4. Information of patients donating lung and thrombus tissues.

ID	Sex	Age (y)	Diagnosis	Tissue
1	Male	7.8	MPP-related pulmonary embolism	Lung, Thrombus
2	Female	6.9	MPP-related pulmonary embolism	Thrombus
3	Male	10.2	Congenital lung cyst	Lung
4	Female	1.4	Congenital lung cyst	Lung
