# Supplementary material for: Neutrophils promote endothelial cell activation in pediatric Mycoplasma pneumoniae pneumonia
Source: JCI Insight. 2026 Sep 22;11(18):e203856. doi: 10.1172/jci.insight.203856 (PMC13596722; doi:10.1172/jci.insight.203856)

Data S1. Uncropped Western blot images corresponding to Figures 2I and 4G.

(a) Corresponds to Figure 2I. Citrullinated histone H3 (citH3) is shown on the left, with the corresponding β-actin loading control on the right ($n = 6$).

(b) Corresponds to Figure 4G. Citrullinated histone H3 (citH3) is shown on the left, with the corresponding β-actin loading control on the right ($n = 6$).

Note: The areas enclosed by the red boxes indicate the representative lanes presented in the main figures.

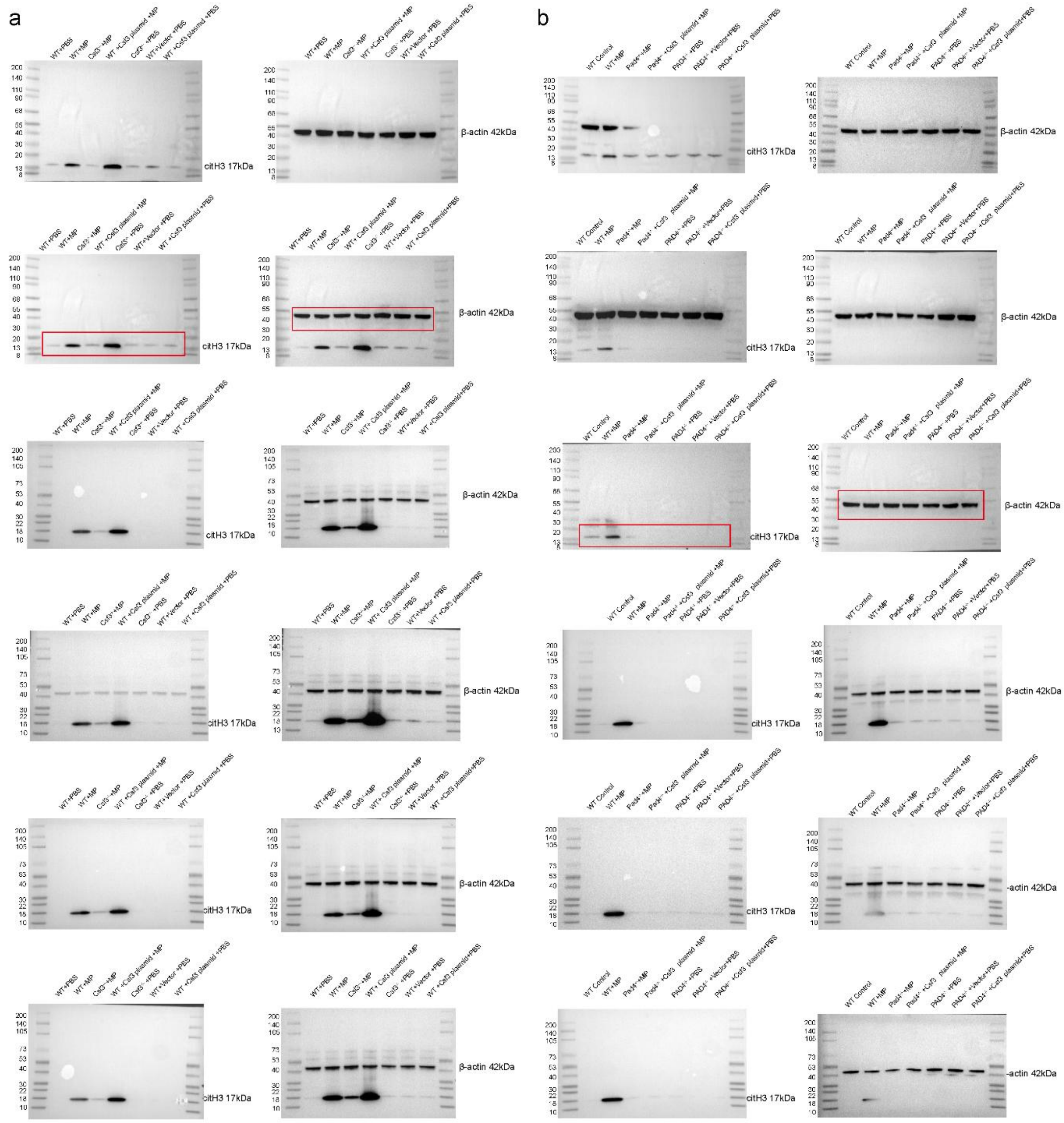

Data S2. Uncropped Western blot images corresponding to Supplemental Figure 8A and Figure 5D.

(a–e) Correspond to Supplemental Figure 8A. ICAM-1 (a), VCAM-1 (b), and P-selectin (c) were detected on the same membrane and share the same GAPDH loading control (d) (n = 3). Panel (e) shows vWF on the left, with its corresponding GAPDH loading control on the right (n = 3).

(f) Corresponds to Figure 5D. TLR2 is shown on the left, with its corresponding β-actin loading control on the right (n = 3).

Note: The areas enclosed by the red boxes indicate the representative lanes presented in the main figures.

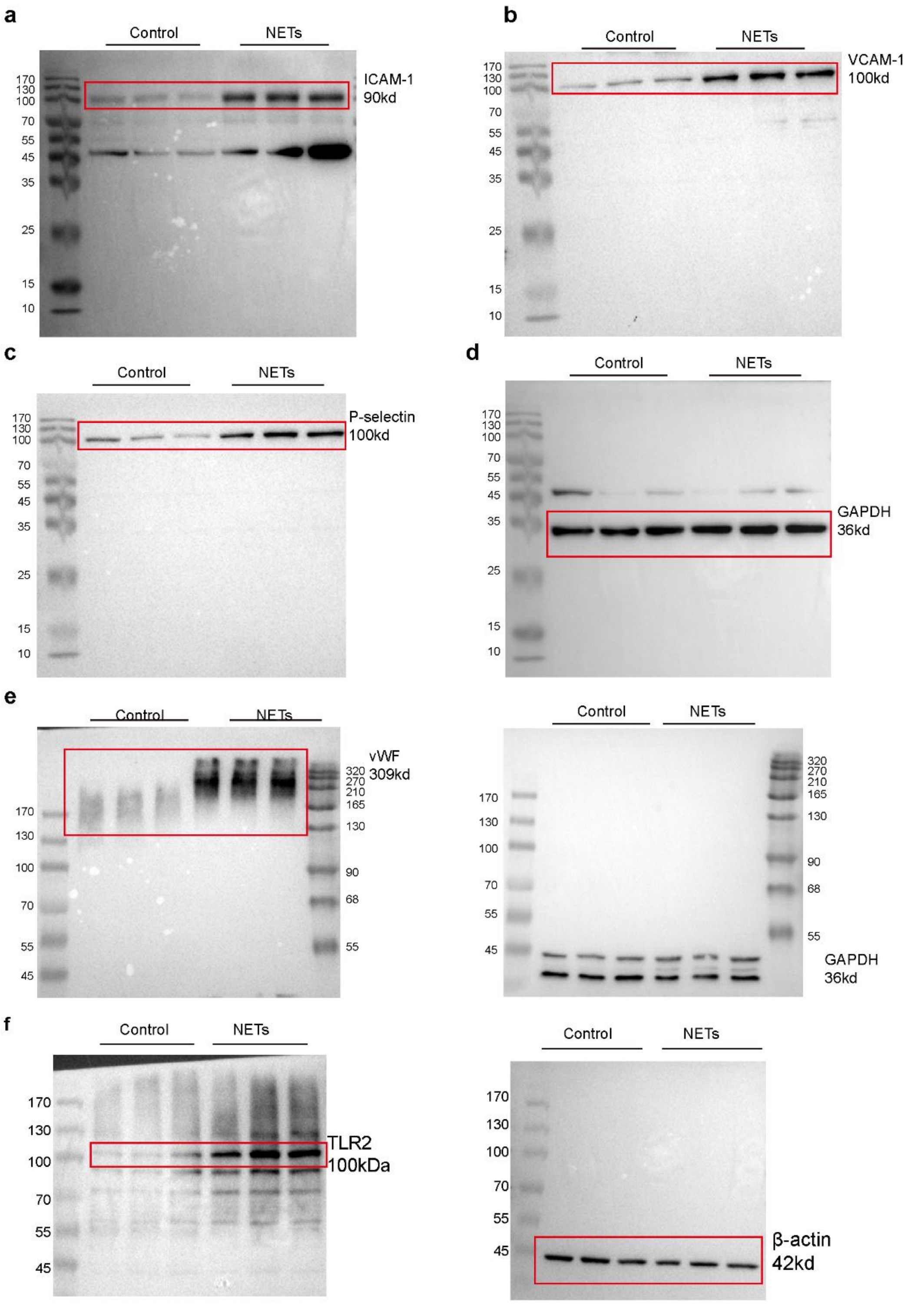

Data S3. Uncropped Western blot images corresponding to Figure 5F.

For each panel, the target protein is shown on the left, with the corresponding β-actin loading control on the right (n = 3). MyD88 (a–b), p-JAK1 (c–d), JAK1 (e–f), p-JAK2 (g–h), JAK2 (i–j), p-STAT3 (k–l), and STAT3 (m–n) are presented. Panels c and e (p-JAK1 and JAK1) were detected on the same membrane and therefore share a single β-actin loading control. Similarly, panels k and m (p-STAT3 and STAT3), and panels l and n (p-STAT3 and STAT3), were detected on the same membranes and share their respective β-actin loading controls. In addition, Data S3 panel b (MyD88) and Data S4 panels b, d, and f (ICAM-1, VCAM-1, and P-selectin) were detected on the same membrane and therefore share a single β-actin loading control.

Note: The areas enclosed by the red boxes indicate the representative lanes presented in the main figures.

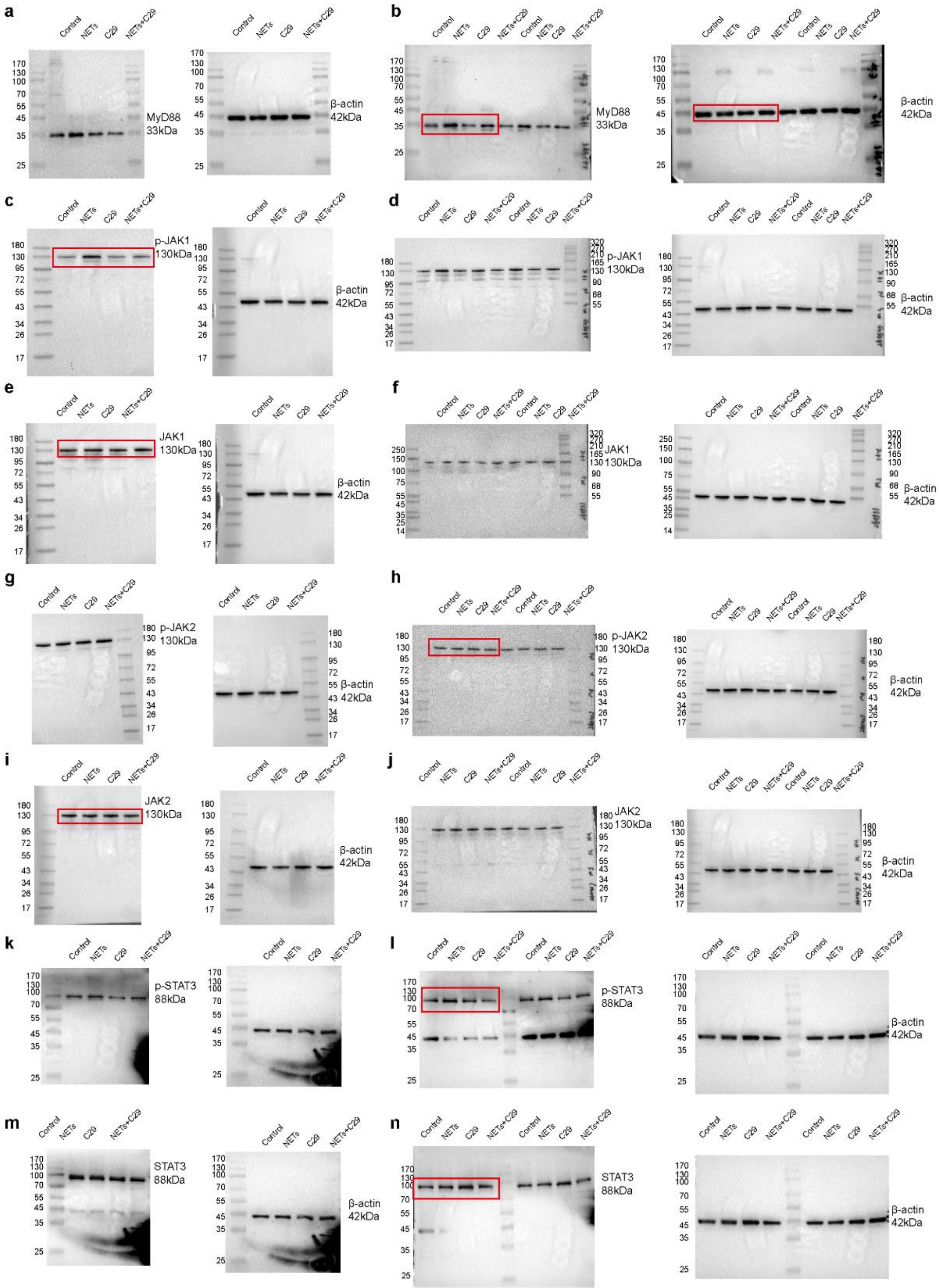

Data S4. Uncropped Western blot images corresponding to Figure 5H.

ICAM-1 (a–b), VCAM-1 (c–d), P-selectin (e–f), and vWF (g–h) are shown on the left, with their corresponding β-actin loading controls on the right (n = 3). Panels a, c, and e (ICAM-1, VCAM-1, and P-selectin) were detected on the same membrane and therefore share a single β-actin loading control. Data S4 panels b, d, and f (ICAM-1, VCAM-1, and P-selectin), together with Data S3 panel b (MyD88), were detected on the same membrane and therefore share a single β-actin loading control.

Note: The areas enclosed by the red boxes indicate the representative lanes presented in the main figures.

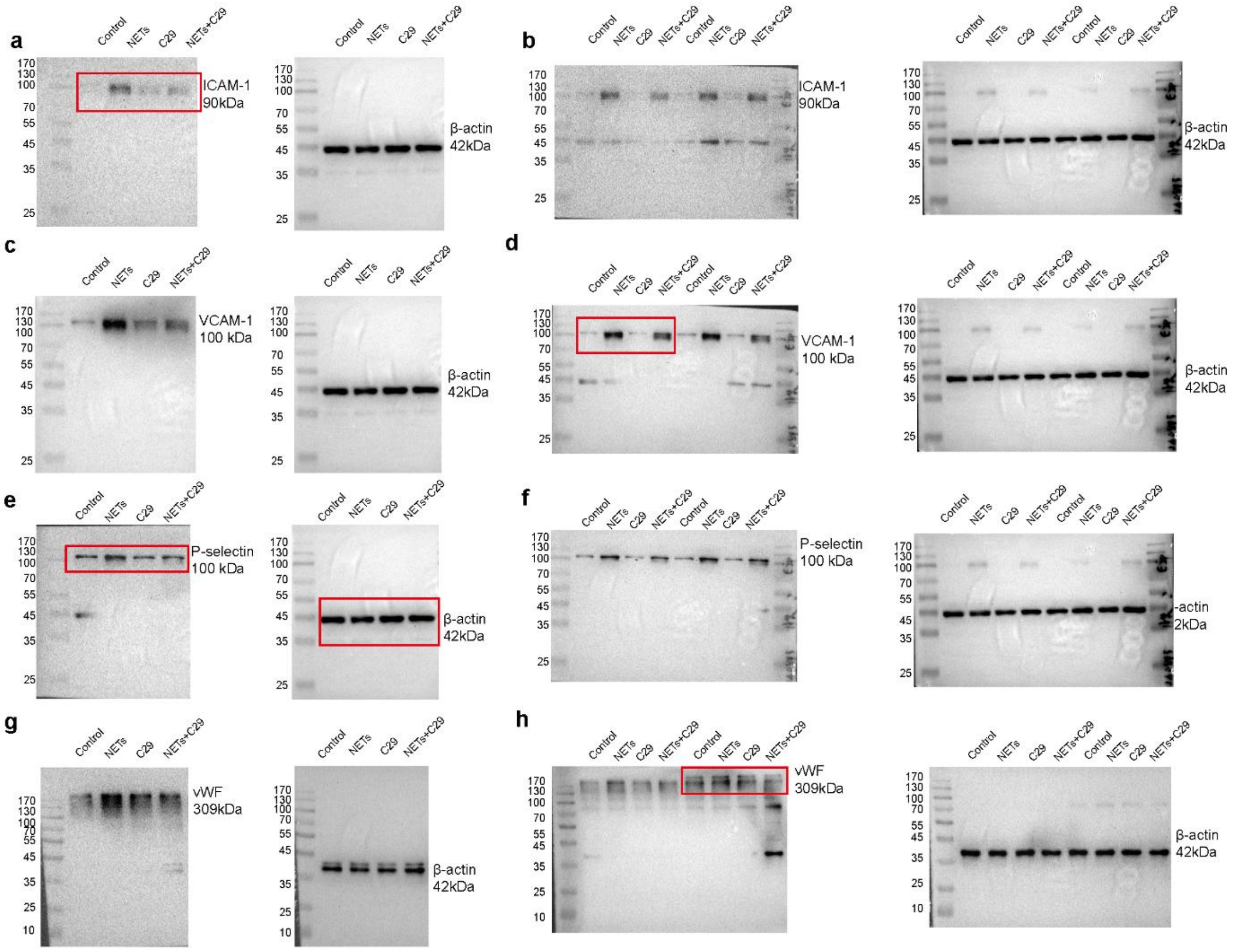

Data S5. Uncropped Western blot images corresponding to Supplemental Figure 8B.

TLR2 (a–b), MyD88 (c–d), p-JAK1 (e–f), JAK1 (g–h), p-JAK2 (i–j), JAK2 (k–l), p-STAT3 (m–n), and STAT3 (o–p) are shown on the left, with their corresponding β-actin loading controls on the right (n = 3). Panels f and h (p-JAK1 and JAK1), panels n and p (p-STAT3 and STAT3) were detected on the same membrane and therefore share a single β-actin loading control.

Note: The areas enclosed by the red boxes indicate the representative lanes presented in the main figures.

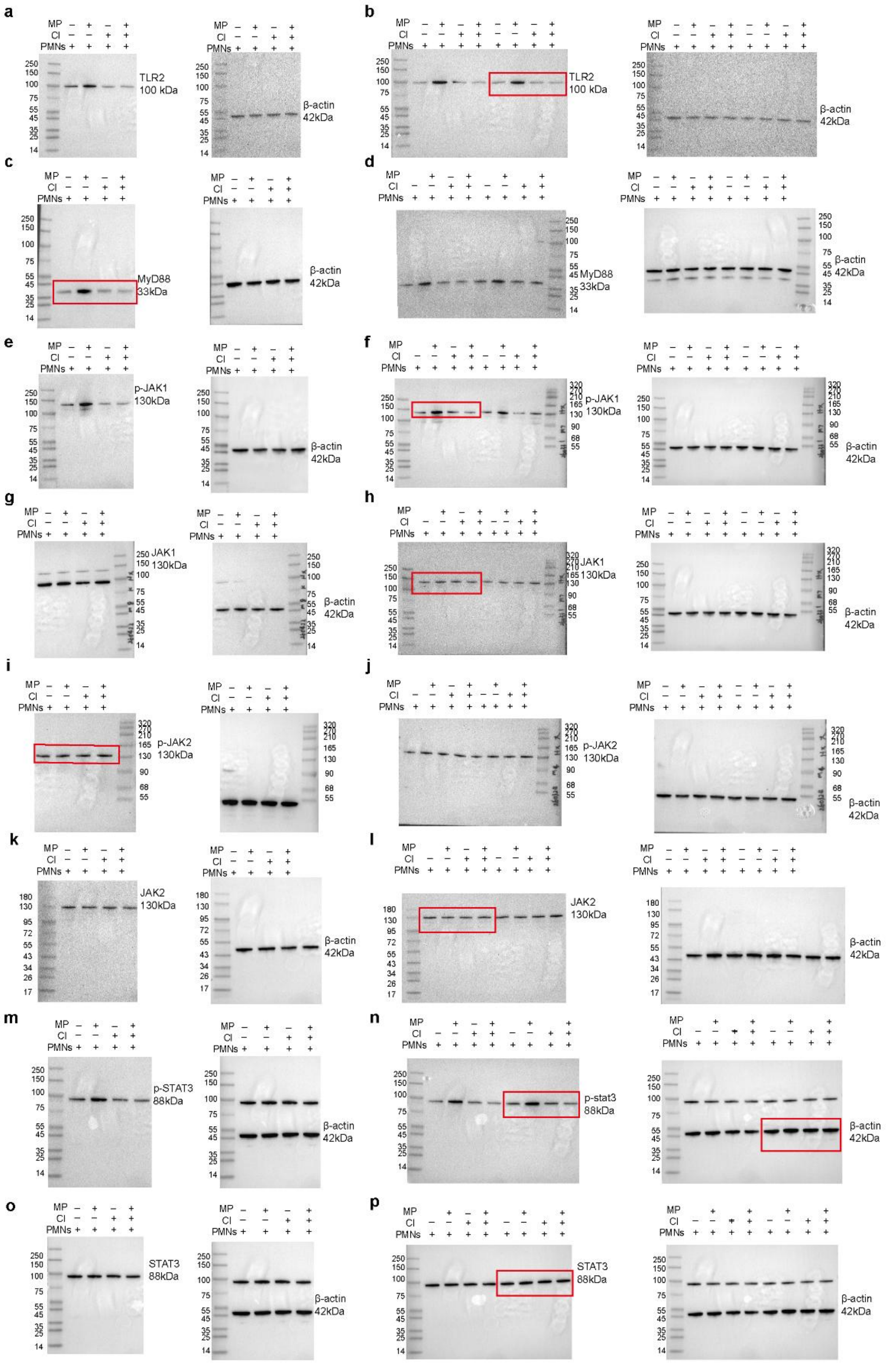

Data S6. Uncropped Western blot images corresponding to Supplemental Figure 8B.

For each panel, the target protein is shown on the left, with the corresponding β-actin loading control on the right (n = 3). ICAM-1 (a–b), VCAM-1 (c–d), P-selectin (e–f), and vWF (g–h) are presented. Panels d and f (VCAM-1 and P-selectin) were detected on the same membrane and therefore share a single β-actin loading control.

Note: The areas enclosed by the red boxes indicate the representative lanes presented in the main figures.

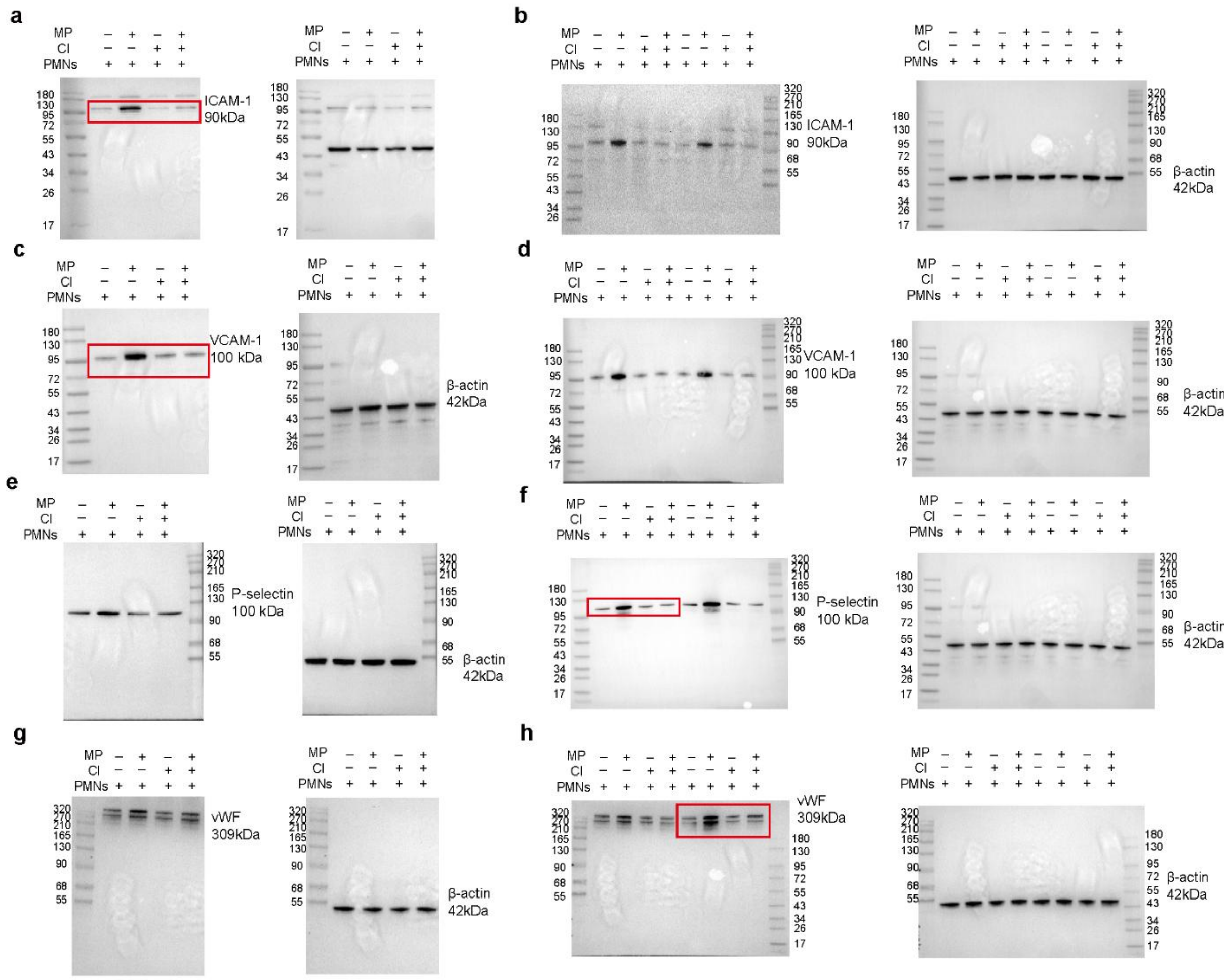

Data S7. Uncropped Western blot images corresponding to Supplemental Figure 8C.

p-JAK1 (a–b), JAK1 (c–d), p-STAT3 (e–f), and STAT3 (g–h) are shown on the left, with their corresponding β-actin loading controls on the right (n = 3). Panels b and d (p-JAK1 and JAK1) were detected on the same membrane and therefore share a single β-actin loading control.

Note: The areas enclosed by the red boxes indicate the representative lanes presented in the main figures.

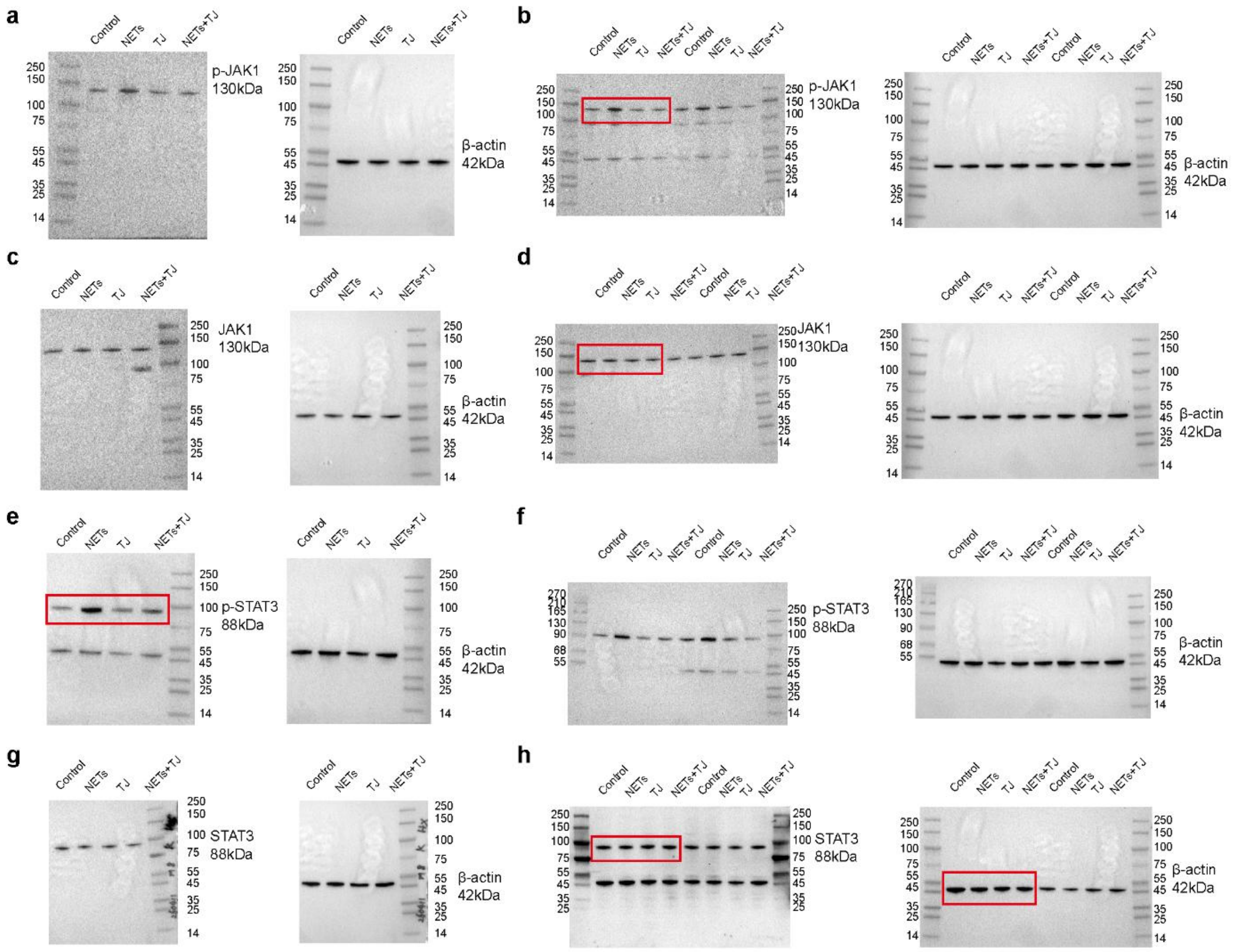

Data S8. **Uncropped Western blot images corresponding to Supplemental Figure 8D.**

p-STAT3 (a–b), STAT3 (c–d), ICAM-1 (e–f), VCAM-1 (g–h), P-selectin (i–k), and vWF (l–m) are shown on the left, with their corresponding β-actin loading controls on the right (n = 3). Panels c and g, as well as panels d and h (STAT3 and VCAM-1), were detected on the same membranes and therefore share the corresponding β-actin loading controls.

Note: The areas enclosed by the red boxes indicate the representative lanes presented in the main figures.

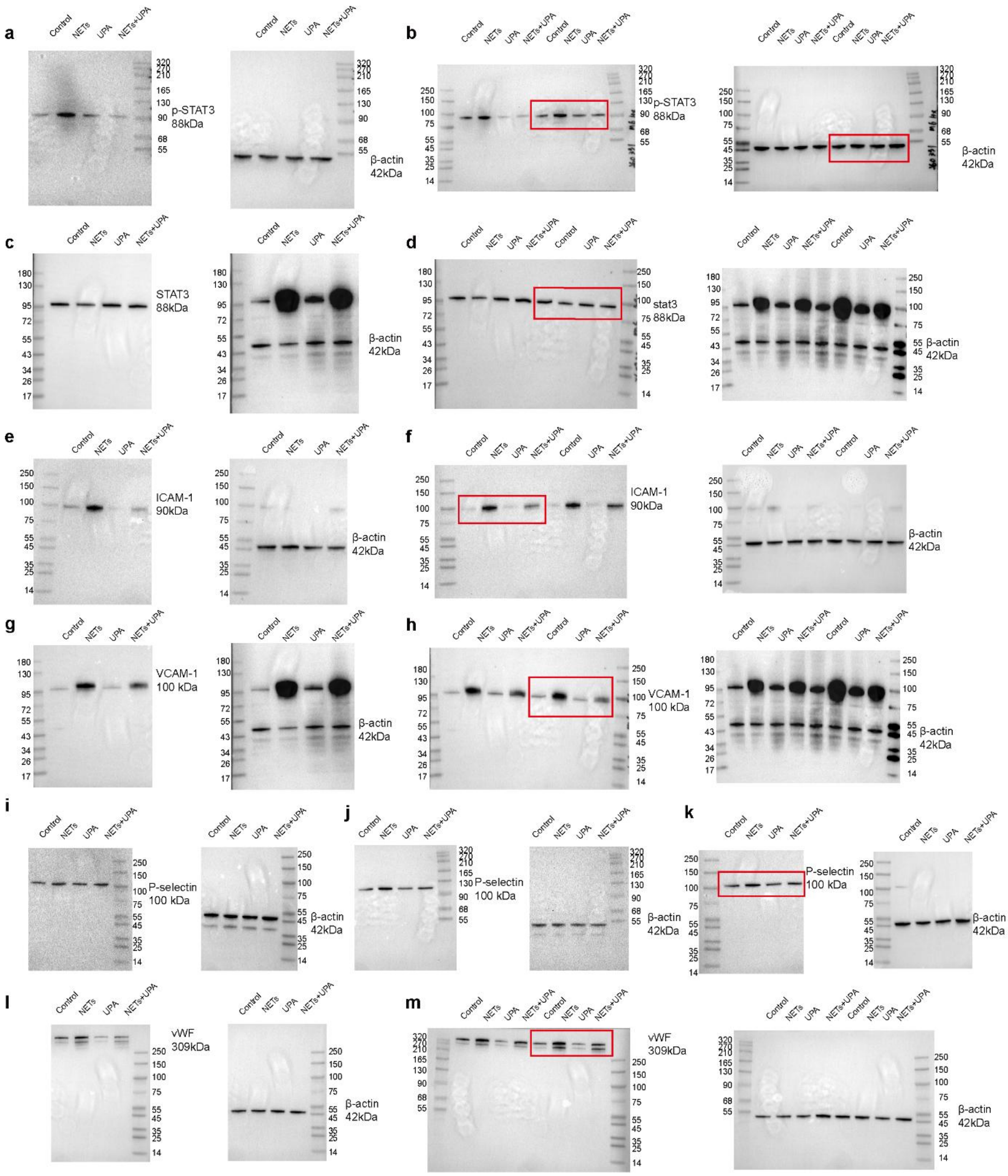

Data S9. **Uncropped Western blot images corresponding to Supplemental Figure 8E.** ICAM-1 (a), VCAM-1 (b), P-selectin (c), and vWF (d) are shown on the left, with their corresponding β-actin loading controls on the right (n = 3).

Note: The areas enclosed by the red boxes indicate the representative lanes presented in the main figures.

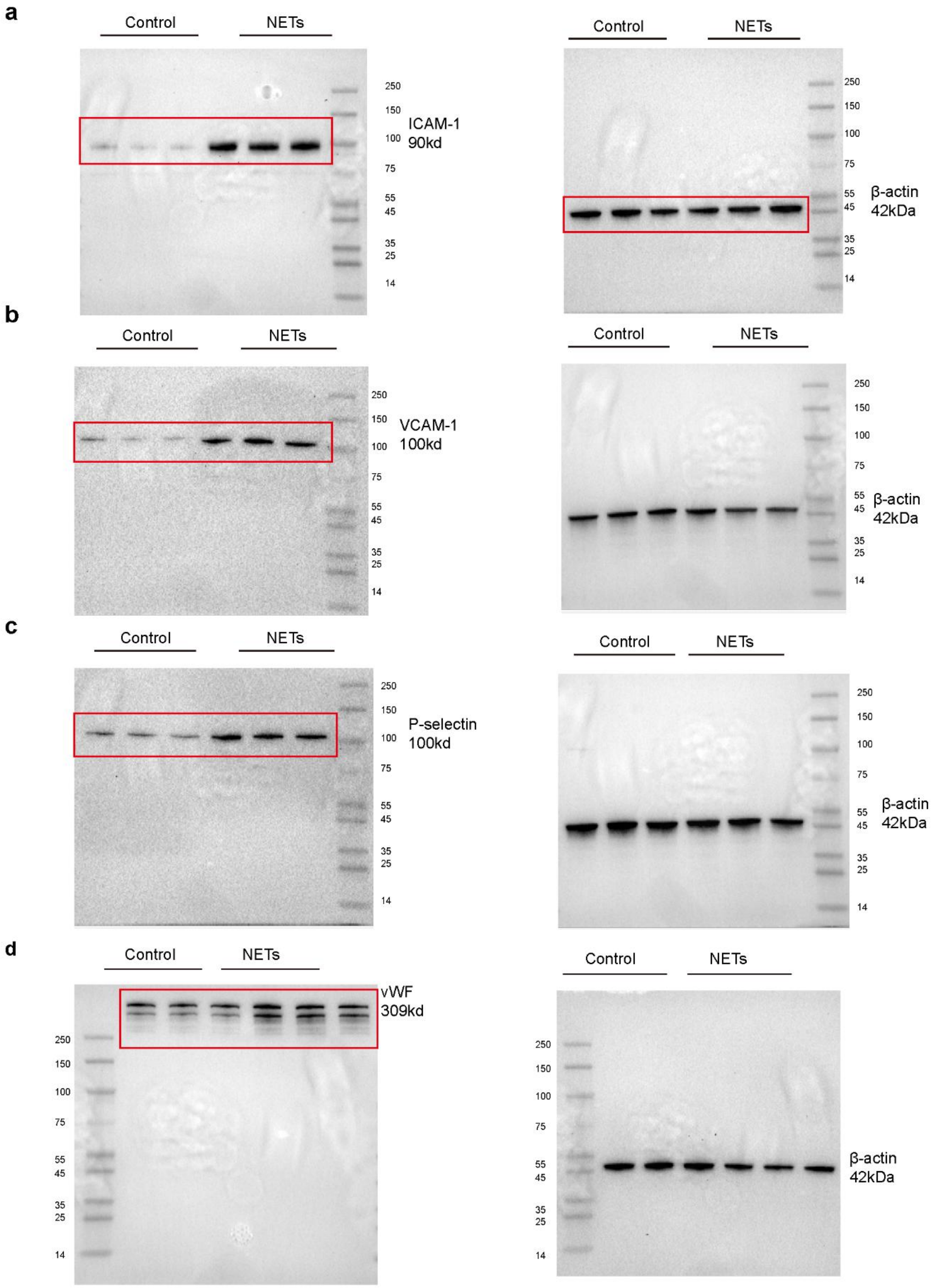

Data S10. Uncropped Western blot images corresponding to Supplemental Figure 8F.

ICAM-1 (a), VCAM-1 (b), P-selectin (c), and vWF (d) are shown on the left, with their corresponding β-actin loading controls on the right (n = 3).

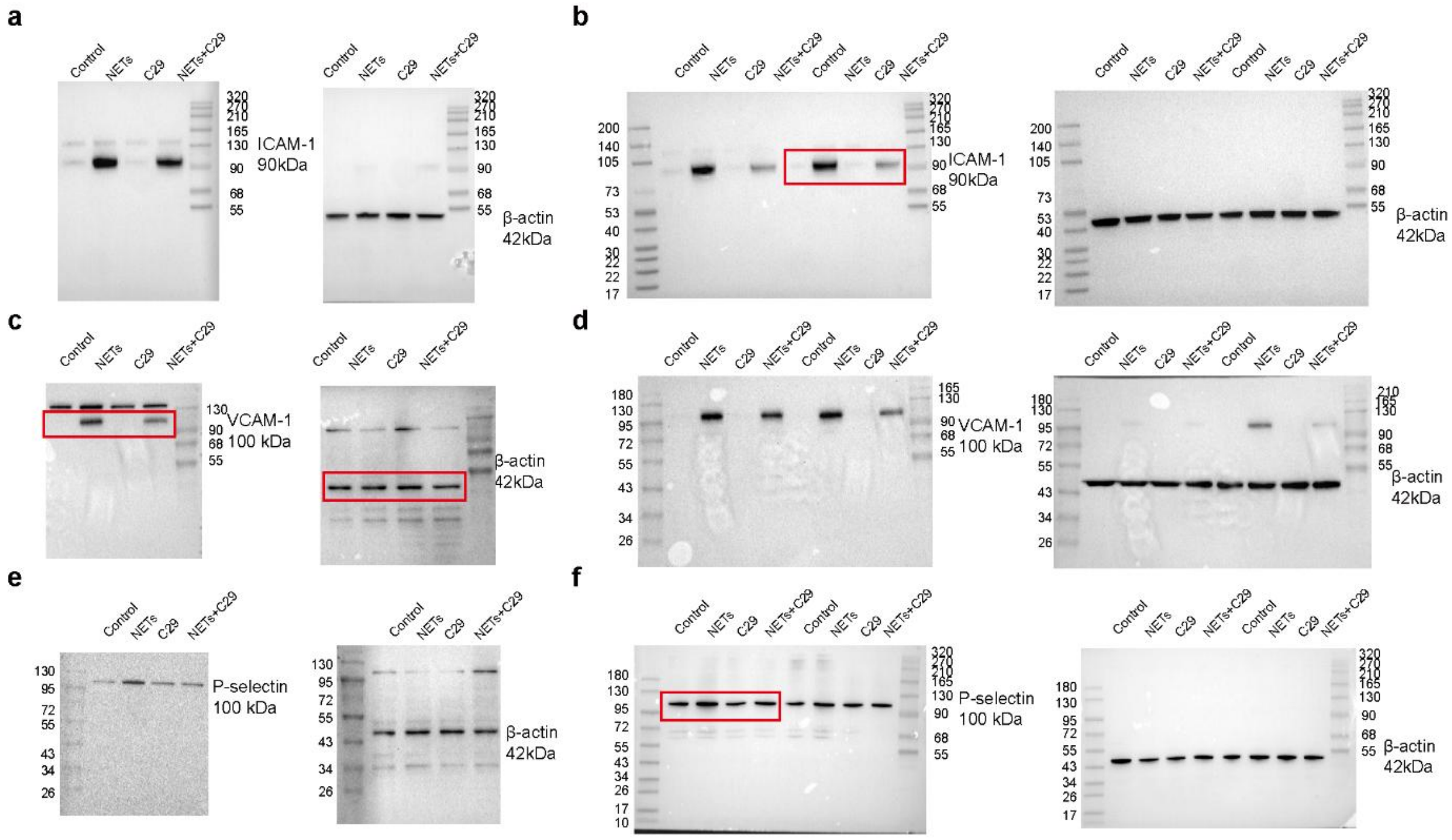

Supplement: Supplementary file 2 [file jciinsight-11-203856-s189.pdf]
